# Supplementary material for: Effectiveness of interactive voice response-call for life mHealth tool on adherence to anti-retroviral therapy among young people living with HIV: A randomized trial in Uganda
Source: PLoS One. 2024 Nov 19;19(11):e0308923. doi: 10.1371/journal.pone.0308923 (PMC11575762; doi:10.1371/journal.pone.0308923)
Supplement: S4 File — (PDF) [file pone.0308923.s005.pdf]

MAKERERE UNIVERSITY

COLLEGE OF HEALTH SCIENCES

ACCEPTABILITY, EFFECT AND COST OF MOBILE-HEALTH ON ART ADHERENCE AND ASSESSMENT OF COVID-19 KNOWLEDGE AMONG YOUTHS: A MIXED METHODS SEQUENTIAL STUDY IN KIRYANDONGO

NCT: 04718974

STUDENT: 2018/HD07/19451U

Dr. Agnes Bwanika Naggirinya

(MBChB, MSc, MMed)

SUPERVISORS

Dr. Rosalind Parkes-Ratanshi (MBBS, MAHons, MRCP, PhD)

Dr. David Meya (MBChB, MMed, CTropMed, PhD)

Dr. Peter Waiswa (MBChB, MPH, PhD)

Dr. Rujumba Joseph (BA. SWSA, MA SSPM, PhD)

A research proposal submitted to Makerere University Directorate of Research and Graduate Training in partial fulfilment for the award of a Doctor of Philosophy degree of Makerere University

July 2020

MAKERERE UNIVERSITY
SCHOOL OF MEDICINE
APPROVED
VALID UNTIL
10 FEB 2021
RESEARCH & ETHICS COMMITTEE
P.O.BOX 7072, KAMPALA

SUPERVISORS SIGNATURE PAGE

1. **Dr. Rosalind Parkes-Ratanshi (MBBS, MA Hons, MRCP, PhD)** ______________________

2. **Dr. David Meya (MBChB, MMed, C Trop Med, PhD)** ______________________

3. **Dr. Peter Waiswa (MBChB, MPH, PhD)** ______________________

4. **Dr. Joseph Rujumba (BA. SWSA, MA SSPM, PhD)** ______________________

MAKERERE UNIVERSITY
SCHOOL OF MEDICINE
APPROVED
VALID UNTIL
10 FEB 2021
RESEARCH & ETHICS COMMITTEE
P.O.BOX 7072, KAMPALA

Contents

OPERATIONAL DEFINITIONS 5
LIST OF ABBREVIATIONS 5
ABSTRACT 7
CHAPTER ONE: INTRODUCTION 9
1.1 Background 9
1.2 Problem Statement 14
1.3 Objectives 15
1.3.1 General Objectives 15
1.3.2 Specific Objectives 15
1.4 Study Justification 15
1.5 Conceptual Framework: 17
1.6 Theoretical Framework: 18
CHAPTER TWO: LITERATURE REVIEW 20
2.1. Youth and mobile phone use 20
2.2 Acceptability of mHealth in HIV positive population 21
2.3 mHealth and ART adherence virological outcomes 23
2.4 mHealth and retention in care 24
2.5 mHealth technologies costs 25
2.6 Hypotheses and Research Questions 26
CHAPTER THREE: METHODS 27
3.1 Study Design: Mixed Methods Sequential study 27
3.2 Study setting 27
3.3 Study population 27
3.4 Materials and Methods 27
Study Objectives: 27
Study Objective 2: 32
Study Objective 3: 44
3.5 Quality control 45
3.6 Ethical consideration 46

MAKERERE UNIVERSITY
SCHOOL OF MEDICINE
APPROVED
VALID UNTIL
10 FEB 2021
RESEARCH & ETHICS COMMITTEE
P.O.BOX 7072, KAMPALA

4. REFERENCES 51

5. List of possible manuscripts 58

6. Appendices 59

i. Budget: 59

ii. Timeframe/work plan 61

iii. Informed Consent Document 62

iv. Assent Document 68

v. CFL FGD Guide Youth Study 74

vii. Revised Stigma Scale 76

viii. REVISED STIGMA SCALE: EVALUATION FORM (Baseline & Study End) 77

ix. DEMOGRAPHIC, SOCIO-ECONOMIC AND MEDICAL EVALUATION FORM 80

x. KNOWLEDGE EVALUATION FORM 88

xi. SEXUAL BEHAVIOR QUESTIONNAIRE 94

MAKERERE UNIVERSITY
SCHOOL OF MEDICINE
APPROVED
VALID UNTIL
10 FEB 2021
RESEARCH & ETHICS COMMITTEE
P.O.BOX 7072, KAMPALA

OPERATIONAL DEFINITIONS

Adherence to ART: Self- reported adherence to ART as depicted on the mHealth dash board or HIV card

Loss to follow-up: Missing 2 visits consecutively, not able to ascertain vitals

mHealth: use of mobile technology e.g. mobile phones and other wireless technology for health-related activities

Retention in care: Continuous engagement in clinical care for 1 year

Virological suppression: Viral load less than 1000 copies/ml

Youth Living with HIV/AIDS: Persons living with human immunodeficiency virus/ acquired immunodeficiency syndrome aged 15 to 24yrs (United Nations General Assembly Resolution A/RES/50/81 ("World Programme of Action for Youth to the Year 200 and Beyond")

LIST OF ABBREVIATIONS

AIDS: Acquired Immuno Deficiency Syndrome

cART: Combined Antiretroviral Therapy

CD4 cell: Cluster Differentiated cells

cDOT: Community based Directly Observed Treatment

MAKERERE UNIVERSITY
SCHOOL OF MEDICINE
APPROVED
VALID UNTIL
10 FEB 2021
RESEARCH & ETHICS COMMITTEE
P.O.BOX 7072, KAMPALA

CFLU: Call for Life Uganda

CITI: Collaborative Institutional Training Initiative

COVID-19: CORONA VIRUS DISEASE 2019

GCP: Good Clinical Practice

HIT: Health Information Technology

HIV RNA: Human Immunodeficiency Virus Ribonucleic Acid

HSP: Human Subjects Protection

IAC: Intensive Adherence Counselling

IDI: Infectious Diseases Institute

IVR: Interactive Voice Response

MoH: Ministry of Health

PEPFAR: President's Emergency Plan for AIDS Relief

PHS: Physical Health Scores

PLWH: People living with HIV

SMS: Short Messaging Service

UBOS: Uganda Bureau of Statistics

UNAIDS: United Nations Programme on HIV and AIDS

UPHIA: Uganda Population-Based HIV Impact Assessment

VL: Viral load

YLWH: Youth living with HIV

MAKERERE UNIVERSITY
SCHOOL OF MEDICINE
APPROVED
VALID UNTIL
10 FEB 2021
RESEARCH & ETHICS COMMITTEE
P.O.BOX 7072, KAMPALA

ABSTRACT

Background:

An estimated 3.9 million[2.1–5.7 million] youth were living with HIV (YLWH) globally in 2017, most of whom live in sub-Saharan Africa (Avert.Org, 2019; UNAIDS, 2018b). Young people are the only population group in which AIDS-related deaths are increasing (UNAIDS, 2019a). AIDS is the leading cause of death among young people in sub-Saharan Africa (Avert.Org, 2019).

In Uganda, there were 160,000 YLWH in 2018 (UNAIDS, 2019b). An estimated 53,000 new HIV infections occurred among youth in 2018, accounting for 26% of HIV incidence in the country (Avert.Org, 2019).

Adherence to antiretroviral therapy (ART) is the principal determinant for achieving and sustaining viral suppression, which decreases progression to AIDS and reduces risk of mortality (CDC, 1998). Few studies have evaluated mHealth adherence tools among youths in resource-limited settings. This study aims to evaluate whether the CFLU mHealth tool improves ART adherence outcomes among youth receiving ART at a rural hospital in Western Uganda.

Corona virus disease (COVID-19) was first declared in December 2019, in Wuhan city, China. The virus spreads through droplet infection from person to person through sneezing or coughing and contact with contaminated surfaces. The outbreak was announced a Public Health Emergency of International concern on 30th Jan 2020 (https://www.who.int/emergencies/diseases/novel-coronavirus-2019/events); and declared a global pandemic by World Health Organization on 11th Mar, 2020. (https://www.health.go.ug/covid/about-corona-virus).

General objective:

To assess acceptability, effect and cost of the CFLU mHealth tool on ART adherence and knowledge of COVID-19among youth initiating and on ART at Kiryandongo Hospital.

Specific Objectives

1a: To assess barriers, enablers of adherence among youth living with HIV in Kiryandongo Hospital at baseline & study end.

MAKERERE UNIVERSITY
SCHOOL OF MEDICINE
APPROVED
VALID UNTIL
10 FEB 2021
RESEARCH & ETHICS COMMITTEE
P.O.BOX 7072, KAMPALA

1b: To assess acceptability of mHealth for HIV adherence support among YLWH in Kiryandongo Hospital at baseline and study end.

2: To assess and effect of the CFLU mHealth tool on ART adherence knowledge on COVID-19 among youths initiating or on ART at 6 and 12 months.

3: To evaluate the cost of the CFLU mHealth adherence tool in comparison to Standard of Care.

Methods

This is a mixed methods sequential explanatory study, with the qualitative study conducted first followed by a randomized control trial and healthcare cost evaluation. The first study is a qualitative study to assess barriers, enablers of adherence and acceptability of mHealth among youths receiving care at Kiryandongo Hospital. The sample size for this study will be a total of 8 focus group discussions (4 at the study start and 4 at study end) or until data saturation occurs, whichever comes first.

The second study is a randomized control trial of YLWH initiating ART to determine the effect of the CFLU mHealth tool on ART adherence. A total of 206 YLWH will be randomized to either Standard of Care (SoC) or CFLU plus SoC. Through the CFLU mHealth tool, participants will interact with a computer through IVR. Participants in the intervention arm will receive pill reminders, clinic visit reminders, health tips messages and functionality to support self-reported symptoms in addition to standard of care. During this second part of the research, youths will be assessed on knowledge of COVID-19 at baseline only.

The third study is nested within the randomized control trial and will assess the cost of the CFLU intervention when compared to SoC.

Conclusion

This project will determine acceptability, effectiveness, knowledge of COVID-19 and cost of delivering pill and clinic visit reminders, and messages on health tips to a population with suboptimal ART adherence. Results from this research will provide valuable insights into mHealth adherence interventions for YLWH and knowledge of COVID-19 in resource-limited settings.

MAKERERE UNIVERSITY
SCHOOL OF MEDICINE
APPROVED
VALID UNTIL
10 FEB 2021
RESEARCH & ETHICS COMMITTEE
P.O.BOX 7072, KAMPALA

CHAPTER ONE: INTRODUCTION

1.1 Background

Human Immunodeficiency Virus (HIV) continues to be a global public health threat. Globally, about 37.9 million [32.7-44.0 million] people were living with HIV (PLWH) in 2018 of whom 25.7 million were in sub-Saharan Africa (UNAIDS, 2018a). An estimated 3.9 million[2.1-5.7 million] youth were living with HIV (YLWH) globally in 2017, most of whom live in sub-Saharan Africa (Avert.Org, 2019; UNAIDS, 2018b). Every week in 2018, 6,200 young women aged 15-24 years became infected with HIV around the world (UNAIDS, 2019b). In sub-Saharan Africa, 80% of new HIV infections among young people aged 15-24, occur in girls. Young women (15-24 years) are twice as likely to be living with HIV as young men (AIDSInfo, 2019).Young people are the only population group in which AIDS-related deaths are increasing (UNAIDS, 2019a). AIDS is the leading cause of death among young people in sub-Saharan Africa (Avert.Org, 2019).

In Uganda, there were 160,000 YLWH in 2018 (UNAIDS, 2019b). An estimated 53,000 new HIV infections occurred among youth in 2018, accounting for 26% of HIV incidence in the country (Avert.Org, 2019). Uganda is implementing the Joint United Nations Programme on HIV and AIDS (UNAIDS) 90-90-90 targets, which aim to achieve 90% of PLWH knowing their status, 90% of those who know their status accessing antiretroviral therapy (ART) and 90% of people on ART achieving virologic suppression by 2020 (UNAIDS, 2014). With regard to attaining global 90-90-90 targets, this means that 81% of people with HIV globally receive ART and 73% of them are virally suppressed. In 2018 in Uganda, 84% of people with HIV knew their status, 72% of PLWH were on ART and 64% of PLWH were virally suppressed [(Avert.Org, 2019; UNAIDS, 2019b). Viral suppression rates were much lower among youth in Uganda in 2017: 44.9% for females and 32.5% in males (UPHIA, 2017b). Young people (15-19 years) in Uganda are more likely to drop out of HIV care than children or adults because of fear of disclosure, stigma and discrimination (Nabukeera-Barungi et al., 2015)

The Infectious Diseases Institute (IDI) is an implementing partner for the President's Emergency Plan for AIDS Relief (PEPFAR) in three of Uganda's seven regions, and provides care for approximately one-in-three of the 1,167,107 Ugandans receiving ART. In 2017, 18 districts supported by the IDI had viral suppression rates between 38% and 87%. Kiryandongo Hospital,

MAKERERE UNIVERSITY
SCHOOL OF MEDICINE
APPROVED
VALID UNTIL
10 FEB 2021
RESEARCH & ETHICS COMMITTEE
P.O.BOX 7072, KAMPALA

one of the HIV care facilities supported by the IDI, was chosen as the study site because it had a total of 1,322 youth (15-24 years) registered in care by June 2019. The number of YLWH accessing care at this hospital has increased over the years, from 1,107 in 2017 to 1,308 in 2018. In 2018, a total of 637 new youth were registered for care at the unit, and by Jun 2019, an additional 218 youths had been registered into care. Overall retention in care at the hospital was 76% in 2017 and improved to 79% in 2018. The viral suppression rate was 72% and 79% in 2017 and 2018, respectively, but this is still below the national and UNAIDS target of 90%.

The World Health Organization (WHO) defines mobile health (mHealth) as "medical or public health practice supported by mobile devices" including mobile phones (WHO, 2011). The WHO recommends mHealth interventions to support HIV adherence. Prior mHealth research studies have shown improved virological outcomes, decreased risk of non-adherence, good retention in care, strengthened health systems through ART adherence and clinic reminder support and improved self-reported adherence and viral load suppression among non-adherent youth (Angella Musiimenta et al., 2018) (Schnall, Bakken, Rojas, Travers, & Carballo-Dieguez, 2015) (A. R. Campbell et al., 2018b; J. E. Haberer et al., 2016) (Rebecca Schnall, M.R.,2010). The Call for Life Study (CFL) was an mHealth intervention trial which aimed to improve outcomes in HIV patients using mobile phone based interactive software support (ClinicalTrials.gov NCT 02953080). Between August 2016 and November 2018, 1,031 PLHIV were screened and 600 participants enrolled (300 per site). They were randomised in a 1:1 ratio, and followed at an urban site (Infectious Diseases Institute Clinic) and a peri-urban HIV clinic (Kasangati Health Centre) to either Standard of Care (SoC) face-to-face counsellor adherence support or SoC plus the Call for Life Uganda (CFLU) mHealth tool. CFLU used Interactive Voice Response (IVR) or text messages delivered to participants via MOTECH-based Connect for Life™ (Janssen, Global Public Health-J&J). The CFLU tool offered daily pill reminders, appointment visit reminders, symptom reporting and management and weekly health information tips. The primary objective of the study was to assess quality of life utilizing medical outcome study scores (MOS) and physical health scores (PHS) in people living with HIV (PLWHIV) accessing mobile phone-based support using the CFLU tool. Despite having no statistically significant observed difference in mean percentage scores of MOS-HIV, Mental Health Scores and PHS at baseline and 6 months between CFL and SoC arms, a 5-point improvement in PHS favoring the intervention group was noted

MAKERERE UNIVERSITY
SCHOOL OF MEDICINE
APPROVED
VALID UNTIL
10 FEB 2021
RESEARCH & ETHICS COMMITTEE
P.O.BOX 7072, KAMPALA

among participants starting first line ART or switching to second line (ANCOVA 4.01; p=0.048) (Rosalind M. Parkes-Ratanshi et al., 2019).

Adherence to ART is the principal determinant for achieving and sustaining viral suppression, which decreases progression to AIDS and reduces risk of mortality (CDC, 1998). Suboptimal adherence includes missed or late doses, sub-therapeutic dosing and treatment interruptions or discontinuations (Vreeman et al., 2014) (Hawkins et al., 2016). Sustaining adherence to ART among youth is challenging perhaps because of treatment fatigue (for those congenitally infected), side effects, drug interactions, drug toxicities, sub-optimal regimens, viral fitness, malabsorption, incomplete emotional and psychological development, lifestyle choices and lack of dedicated health services (Edward M Gardner, Burman, Steiner, Anderson, & Bangsberg, 2009). Sub-optimal adherence leads to sub-therapeutic plasma concentrations of antiretroviral drugs which may speed up the development of drug resistance, limit future drug options and permit transmission of resistant strains (Oyugi et al., 2007). These factors highlight the difficulty of maintaining high adherence among youth.

HIV services oriented toward adult care are perceived by youth as poor quality and intimidating which discourages retention in care (WHO, 2016). Thus, youth are more likely to drop out of HIV care than adults (Koirala et al., 2017) (Hudelson & Cluver, 2015). There is urgent need to develop interventions to improve ART adherence among youth. Few studies have evaluated mHealth adherence tools among youths in resource-limited settings. This study aims to evaluate whether the CFLU mHealth tool improves ART adherence outcomes among youth receiving ART at a rural hospital in Western Uganda.

When initiated on ART, a high level of sustained adherence is necessary to suppress viral replication and improve immunological and clinical outcomes, decrease risk of developing ARV drug resistance and reduce the risk of HIV transmission (Agwu et al., 2012). The proportion of youth with viral load suppression in Uganda is suboptimal; only 44.9% of females and 32.5% of males achieved viral suppression in 2017. Effective implementation of evidence-based interventions is needed if the last two 90s of the UNAIDS 90-90-90 targets are to be achieved.

Previous work has shown that mHealth interventions improve virological outcomes (Lawrence Mbuagbaw et al., 2011) (A. M. Ammassari, et al, 2011), decrease risk of non-adherence (Lester et al., 2010), improve retention in care (van der Kop ML, 2013), strengthen health systems through ART adherence and clinic reminder support (Natalie Leon, 2012) and

MAKERERE UNIVERSITY
SCHOOL OF MEDICINE
APPROVED
VALID UNTIL
10 FEB 2021
RESEARCH & ETHICS COMMITTEE
P.O.BOX 7072, KAMPALA

improve self-reported adherence and viral load suppression among non-adherent youth (Garofalo et al., 2016). The Call for Life Uganda mHealth tool was developed by Janssen Global Public Health Research and Development, in close collaboration with the Infectious Diseases Institute (IDI), and tailored it to the needs of PLHIV in Uganda. Call for Life Uganda (CFLU) is based on the CONNECT FOR LIFE™ technology and the MOTECH platform. CONNECT FOR LIFE™ is an m-health technology initially developed as a community information technology platform for health initiatives to improve care in patients in developing countries. The platform interacts with patients using basic mobile phone technology and with healthcare providers through a web-based interface. This Interactive Voice Response (IVR) software was first piloted by Janssen and the Grameen Foundation in India, and was called "Treatment advice by Mobile Alerts"(TAMA) to support HIV patients in India (http://motechsuite.org/index.php/implementations).

The Call for Life Uganda (CFLU) study randomized 600 PLHIV to receive enhanced adherence support with daily SMS or Interactive Voice Response (IVR) call reminders, standardized health education messages and facility based standard of care (intervention arm) or facility standard of care only (SOC) in a 1:1 ratio (ClinicalTrials.gov NCT 02953080). A total of 331 health education messages were designed and developed by IDI and Straight Talk Foundation-Uganda staff, using National HIV prevention guidelines. These messages were piloted with expert patients at the Infectious Diseases Clinic (IDC) at Mulago Hospital in Kampala, Uganda, translated into three local languages (Luganda, Kiswahili and Runyakitara) and relayed to patients using the platform once a week or on request during the daily pill reminder call. Patients could also call in using a toll-free number to request health education messages, or report any symptoms related to the disease, drug or any concomitant illness. The CFLU study participants were prospectively followed 6 monthly for 24 months. The study's main outcome was to assess quality of life utilizing Medical Outcome Study Scores-HIV (MOS-HIV), Mental Health Scores (MHS) and Physical Health Scores (PHS) in PLWHIV accessing mobile phone-based adherence using the CFLU tool. There was no statistically significant observed difference in mean percentage score of MOS-HIV, MHS and PHS at baseline and 6 months between CFLU and SoC arms. In those starting first line ART or switching to second line, there was a significant improvement in PHS (ANACOVA 4.01, p=0.048) of 5-points difference or greater. Qualitative data suggested high acceptance and positive experiences with the CFLU tool in PLHIV. Nearly all participants (98%) selected interactive voice response over text messages (Rosalind M. Parkes-Ratanshi et al., 2019).

MAKERERE UNIVERSITY
SCHOOL OF MEDICINE
APPROVED
VALID UNTIL
10 FEB 2021
RESEARCH & ETHICS COMMITTEE
P.O.BOX 7072, KAMPALA

IVR is a useful and flexible mHealth tool which can be used for computer-assisted screening of a population (Corkrey, Parkinson, Bates, Green, & Htun, 2005) or in-depth daily assessments of risk behaviours (Perrine, Mundt, Searles, & Lester, 1995; Schroder, Johnson, & Wiebe, 2007). It can also be used as an external aid to promote participant medication adherence (Hettema, Hosseinbor, & Ingersoll, 2012; Reidel, Tamblyn, Patel, & Huang, 2008) or used as a medium to relay health messages or advice intended to improve health behaviours(Corkrey et al., 2005). IVR may be programmed to receive incoming calls from participants, place automated outgoing calls, or a combination of both (Kerstin E. E. Schroder & Johnson, 2009). Using this strategy in CFLU, this mHealth tool is able to track study participants, allows investigators to receive data nearly in real-time and to intervene should a problem occur with the study participant, before it is too late. The Call for Life System has functionality to support individualised pill reminders, visit reminders, health message calls and self-reported symptoms and management. mHealth apps have been developed for asthma, chronic obstructive pulmonary disease, diabetes, malaria, tuberculosis, and HIV as well as for specific populations, e.g., mother and baby (Qudrat-Ullah & Tsasis, 2017). Call for Life System can be adopted for use in Ugandan YLWH to aid in ART and clinic adherence, knowledge sharing and symptom management.

Coronavirus disease (COVID-19), a viral infection with high infectivity and human-human transmissibility, is caused by severe acute respiratory syndrome coronavirus 2 (SARS-CoV-2). The SARS-CoV-2 is probably of zoonotic potential, due to the allegations that the virus was transferred to people at a food market that trades in wildlife in Wuhan, China (Yang et al., 2020).

The state of lock-down due to COVID-19 in many parts of the world has led to the halting of services (Roy et al., 2020)and Uganda health service provision has not been spared either. Uganda got first country wide lockdown on 22nd Mar 2020, this lockdown disrupted treatment continuity and globally the supply chains were halted and most patients in chronic care faced challenges (Ebrahim et al., 2020). There is moderate awareness and knowledge of COVID-19 among educated population in India(Roy et al., 2020), but little is document among youths in rural areas, literature serach revealed one documentation on knowledge, attitude and practices of HCW in Makerere University teaching hospital(Olum, Chekwech, Wekha, Nassozi, & Bongomin). A poor understanding of the disease among youths can result in delayed identification and treatment leading to rapid spread of infections.

MAKERERE UNIVERSITY
SCHOOL OF MEDICINE
APPROVED
VALID UNTIL
10 FEB 2021
RESEARCH & ETHICS COMMITTEE
P.O.BOX 7072, KAMPALA

1.2 Problem Statement

About 5 million[2.1–5.7 million] youth were living with HIV (YLWH) globally in 2018, most of whom live in sub-Saharan Africa (Avert.Org, 2019; UNAIDS, 2018b) (https://www.who.int>adolescents). AIDS is the leading cause of death in Africa (https:// www.avert.org). Globally, 30% of all new HIV infections occur among youth 15-24 years (UNAIDS).

Youths living with HIV (YLWH) have low viral load suppression rates due to poor ART adherence compared with older adults. According to the UPHIA 2017 report, a household-based national survey conducted in Uganda from August 2016 to March 2017, approximately 55% of young females and 68% of young males had unsuppressed HIV viral load (UPHIA, 2017a). Persistent viremia is associated with development of drug resistance, low immunity which increases the risk of opportunistic infections and death (Oyugi et al., 2007). AIDS-related illnesses remain the second leading cause of death for young women aged 15-24 years in Africa (UNAIDS, 2017).

Improved linkage and retention in care coupled with improved adherence for viral suppression are paramount in contributing to the UNAIDS second and third 90s and improving the HIV treatment continuum (Babalola Stella, 2017). Poor viral load suppression is mainly due to non-compliance to clinic visits, lack of knowledge on ART, inconsistent swallowing of pills and poor pill adherence (Lee-Ann Crystal, 2017). We hypothesize that early follow-up of missed appointments coupled with adherence support for the first 6 to 12 months of initiating ART could overcome the current problem faced by youth. Existing evidence indicates that Intensive Adherence Counselling (IAC) for those who are not suppressed may not be effective. A retrospective chart review demonstrated that suppression rates were low among ART-treated children with virological failure that completed the three recommended IAC sessions (Nasuuna et al., 2018).

Mobile health interventions have been effective in improving ART adherence and viral suppression is a proxy measure for the adherence. A study of SMS text messaging in Italy found that the proportion with undetectable HIV RNA viral load increased from 42.3% at baseline to 76.8%, 71.5%, 76.2% at months 3, 6, and 9, respectively (P=0.001) (A. Ammassari et al., 2011). In rural Uganda, text messaging was effective in increasing ART adherence and the authors recommended that it be explored in youth (J. E. Haberer et al., 2016; A. Musiimenta et al., 2018).

MAKERERE UNIVERSITY
SCHOOL OF MEDICINE
APPROVED
VALID UNTIL
10 FEB 2021
RESEARCH & ETHICS COMMITTEE
P.O.BOX 7072, KAMPALA

Poor ART adherence due to missed clinic visits and lack of drug refills, forgetfulness, stigma comorbid conditions and psychosocial issues are the leading causes of poor viral suppression in youth. Current use of Intensive Adherence Counselling for non-adherent, non-suppressed patients has not been shown to be effective, and there is still a gap in providing daily pill reminders, visit reminders, HIV and ART knowledge and adherence support. All this can be achieved through use of IVR, which this study will provide through the Call for Life tool.

1.3 Objectives

1.3.1 General Objectives

To assess acceptability, cost and effect of mHealth tool on ART adherence and knowledge of COVID-19 among youths initiating ART in Kiryandongo.

1.3.2 Specific Objectives

I. To assess barriers, enablers of adherence for ART among Youth in Kiryandongo at baseline and study end.

II. To assess acceptability of mHealth for ART adherence among Youth in Kiryandongo at baseline and study end

III. To evaluate effect of the mHealth CFLU tool on ART adherence at months 6 and 12

IV. To evaluate the cost of the mHealth CFLU tool in comparison to standard of care at 12 months

V. To assess knowledge of COVID-19 among youths in rural setting

1.4 Study Justification

Over 55% of YLWH in Uganda are not virologically suppressed. Patient-centered approaches exploiting mHealth have been shown to improve adherence to ART and viral suppression. Due to poor clinic attendance by youth, Intensive Adherence Counselling (IAC) alone cannot help suppress detectable viral load in this group. Evidence-based interventions are needed to complement standard of care adherence counseling. The 2013 WHO consolidated guidelines on the use of antiretroviral drugs for treating and preventing HIV infection, suggested use of automated Short Message System (SMS) reminders of scheduled clinic visit to improve clinic attendance (WHO, 2013). Experience with the recently concluded mHealth study (Call for Life

MAKERERE UNIVERSITY
SCHOOL OF MEDICINE
APPROVED
VALID UNTIL
10 FEB 2021
RESEARCH & ETHICS COMMITTEE
P.O.BOX 7072, KAMPALA

Study) at IDI shows that 98% of the study participants preferred voice calls instead of text messages. Participants cited confidentiality, voice tone and privacy related to voice calls as reasons for this preference. CFLU is an open source mobile-phone based software designed to send short messaging services (SMS) and Interactive Voice response (IVR) calls. Additional services include pill reminder support, adherence support, visit appointment reminders, symptom report management and health information tips (FIGURE1). However, this mHealth intervention has not been implemented among the YLWH in Uganda.

It is estimated that 53.2 % of Ugandans have access to mobile phones; 23.2 million Ugandans were mobile phone subscribers in 2016 (UBOS, 2017). According to the Pew Research Centre, Ugandan youth are more likely to use mobile phones than adults (Jacob 2015). This study found that 66% and 51% youth had ever used text messages or sent/viewed videos or pictures, respectively, highlighting their ease and comfort with cell phone use. In a systematic review of 25 mHealth programs targeting youth sexual reproductive health (SRH) found that most (67%) were conducted in Africa. Nearly all (87%) used mHealth as a health promotion tool to facilitate knowledge and behavior change. A minority (18%) used mHealth to link users to essential SRH services including family planning counselling services, medical abortion, post–abortion care and HIV treatment. Evidence is emerging that mobile phones are an effective way to reach youth and to achieve knowledge and behavior change (Ippoliti & L'Engle, 2017).

Few studies have evaluated mHealth adherence tools among youths in resource-limited settings. This study aims to evaluate whether the CFLU mHealth tool improves ART adherence outcomes among youth receiving ART at a rural hospital in Western Uganda.

This study will join Uganda in implementing the Joint United Nations Programme on HIV and AIDS (UNAIDS) 90-90-90 targets, which aim to achieve 90% of PLWH knowing their status, 90% of those who know their status accessing antiretroviral therapy (ART) and 90% of people on ART achieving virologic suppression by 2020 (UNAIDS, 2014).

MAKERERE UNIVERSITY
SCHOOL OF MEDICINE
APPROVED
VALID UNTIL
10 FEB 2021
RESEARCH & ETHICS COMMITTEE
P.O.BOX 7072, KAMPALA

1.5 Conceptual Framework:

FIGURE 2: CONCEPTUAL FRAME WORK

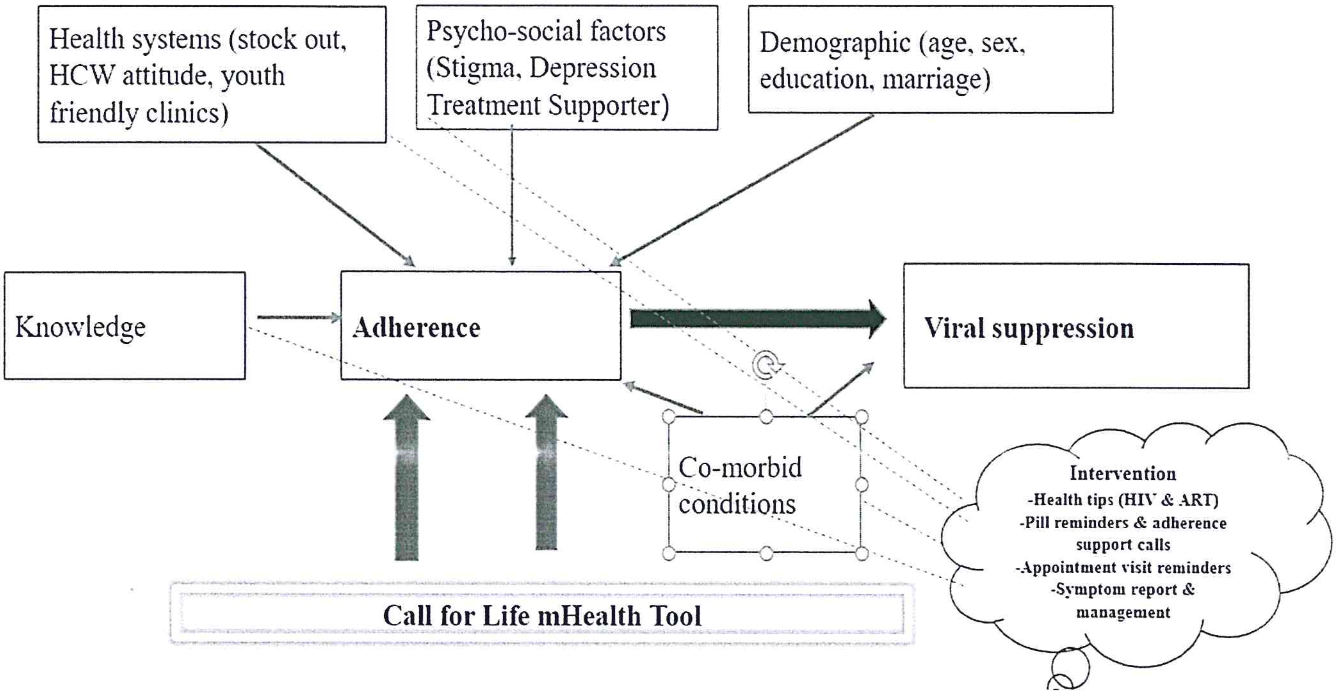

MAKERERE UNIVERSITY
SCHOOL OF MEDICINE
APPROVED
VALID UNTIL
10 FEB 2021
RESEARCH & ETHICS COMMITTEE
P.O.BOX 7072, KAMPALA

1.6 Theoretical Framework:

Theoretical framework based on the Information-Motivation-Behavioral Skills Model (J Fisher Fisher 1992, 2000)

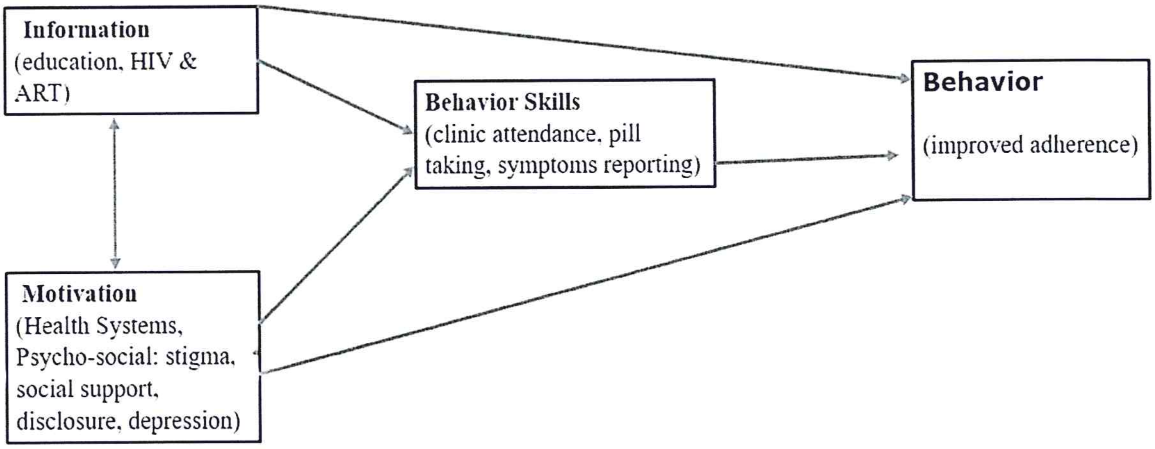

J. Fisher and Fisher (1992)
W. Fisher and Fisher (1993)

The IMB Model, (Fisher, Fisher and Harman, 2003) was developed to explain HIV risk and preventive behavior. The model recognizes three key constructs needed to engage in a given health behavior: information, motivation and behavioral skills. Information involves the provision of correct information and dismissing of incorrect information. Motivation refers to both personal and social motivation (perceived social support for engaging in certain behavior) and behavioral skills refer to perceived self-efficacy for carrying out a certain behavior (Fisher, Fisher, & Harman, 2003).

Based on the IMB model (Jeffrey D.Fisher, 1992), existing information affecting adherence will be elicited first, and this information may be around social-psychological factors, anthropological factors, stigma and HIV/ART knowledge among youth. Most of the information regarding adherence of ART in this group will be assessed at baseline and study end during the qualitative data collection, stigma will be prospectively collected under the second objective.

MAKERERE UNIVERSITY
SCHOOL OF MEDICINE
APPROVED
VALID UNTIL
10 FEB 2021
RESEARCH & ETHICS COMMITTEE
P.O.BOX 7072, KAMPALA

Secondly, the mHealth tool has been used previously for health promotion where it improved knowledge in sexual reproductive health and facilitated behavior change (Ippoliti & L'Engle, 2017). The IMB model posits that provision of information and motivation influences behaviours. We hypothesize that the CFLU tool will address existing knowledge gaps among YLWH through provision of health messages about HIV, ART and medication adherence. Daily pill reminders and visit reminders will motivate youth and will help to address forgetfulness and busy schedules, factors which were the main causes of medication non-adherence and missed visits in the Call for Life study. Addressing these factors will influence adherence behaviours.

Prior work suggests that patients with higher HIV knowledge are twice as likely to keep their appointments as those who are less knowledgeable. Knowledge of HIV coupled with a good client-provider was associated with increased CD4 count and a five-fold likelihood of achieving undetectable VL (Jones, Cook, Rodriguez, & Waldrop-Valverde, 2013). Lack of knowledge of correct dosing and time to take drugs was associated with non-adherence in those starting ART (Miller et al., 2003). HIV knowledge has also been shown to be associated with improved quality of life (Swindells et al., 1999).

Lastly, evaluation of the effect mHealth tool will include assessment of stigma scores at baseline and follow-up, HIV knowledge at baseline and follow-up, and adherence as measured by clinic attendance (visit adherence), phone interaction and viral suppression.

The IMB model was chosen because the intervention will provide information through health tip messages and motivation through daily calls. According to prior recipients of the CFLU mHealth intervention, voice calls and voice tones encouraged and motivated them to continue taking medication, which impacted their behaviours including honoring clinic visit appointments, taking medication on time and willingness to report any symptoms they may have had. These factors may have improved clinic attendance, ART adherence and viral suppression. This model is a better fit for the CFLU mHealth tool than similar behavior models e.g. the Capability-Opportunity-Motivation-Behavioral (COM-B) model.

MAKERERE UNIVERSITY
SCHOOL OF MEDICINE
APPROVED
VALID UNTIL
10 FEB 2021
RESEARCH & ETHICS COMMITTEE
P.O.BOX 7072, KAMPALA

CHAPTER TWO: LITERATURE REVIEW

2.1. Youth and mobile phone use

The use of mobile phones and other mobile technologies for improving health through research and practice is growing quickly, particularly in areas with difficult-to-reach populations or where the healthcare infrastructure is less developed (Qudrat-Ullah & Tsasis, 2017). In the Kampala Youth Survey 2014, it was noted that nearly half of the youth owned and used phones daily (Swahn , Braunstein, & Kasirye, 2014). The study assessed psychosocial characteristics based on phone ownership and use and found that those who initially owned and used mobile phones were educated, young, wealthy, male and living in urban areas (Swahn et al., 2014). More recently, cell phone use has expanded to include those living in rural areas, the elderly, and those with less resources to an estimated 23 million subscribers in Uganda (UBOs, 2018). In the prior Kampala Youth Survey (2011), 47% of youth reported owning a mobile phone. Ownership did not vary by sex but was more common among youth above 18 years of age. Mobile phone ownership was more common among those who reported taking care of themselves at night, who reported current drug use and who reported trading sex for money, food or other goods.

As the burden of HIV continues to grow among adolescents and youth, mHealth technology could address many of the healthcare needs of YLWH including adherence to HIV medications, retention in care and self-management (Steve Kanters, 2016), which are critical needs for this priority population. mHealth technology could bridge the divide in healthcare delivery in underserved minority groups. Since ownership of a mobile device is common among the youth, use of mHealth can help increase engagement in HIV care (Ippoliti & L'Engle, 2017). Due to the high incidence of HIV among minorities, adolescents and underserved youth, and their reliance on mobile technology, it is imperative to develop health information technology (HIT) tools tailored to the needs of this population (Schnall, Bakken, Brown Iii, Carballo-Dieguez, & Iribarren, 2016; R. Schnall et al., 2016) (Schnall et al., 2015).

Overall HIV incidence in Uganda has been declining since 2010. However, the new HIV infections are disproportionately concentrated among young people. Given high penetration of mobile phone technology, there is growing support for using mobile health (mHealth) programs to reach vulnerable populations (Chang et al., 2011; Chib & Harris). Given that nearly half of youth in

MAKERERE UNIVERSITY
SCHOOL OF MEDICINE
APPROVED
VALID UNTIL
10 FEB 2021
RESEARCH & ETHICS COMMITTEE
P.O.BOX 7072, KAMPALA

Uganda own or use mobile phones daily, new research is needed to determine next steps for mobile health (mHealth), including the feasibility of using mobile phones for data collection and interventions with this hard-to-reach population (Swahn et al, Aug 2014).

2.2 Acceptability of mHealth in HIV positive population

Several studies on the acceptability and feasibility of mHealth in PLWH have been done, but few of these were conducted among YLWH. One study in South Africa examined short message service (SMS) and/or community-based directly observed antiretroviral therapy (cDOT) as interventions to improve ART adherence for preventing mother- to-child HIV transmission. This study showed that SMS and or cDOT are feasible in this setting. However, safe HIV status disclosure to treatment supporters and confidentiality of text messaging content about HIV and ART were deemed to be crucial implementation factors (Nachega et al., 2016).

Few mHealth studies have been conducted among youths despite several studies of mHealth apps (Amith, Loubser, Chapman, Zoker, & Rabelo Ferreira, 2012; Luxton, June, & Chalker, 2015), usability of the devices (Doris Caliz), and negative effects of sexualized video apps (Burnay, Bushman, & Laroi, 2019). Brown et al, conducted 6 focus group discussions to explore adolescent use of mobile technology for meeting their health information needs in Columbia, when given smart phones with mHealth apps. The study assessed health seeking information behavior among adolescents and found that the main motivation for using mHealth app was their performance speed and information needs. Barriers for use of the app included use of non-phone app technology (i.e. phone, books), non-mobile resources (i.e. parents, teachers, siblings), and other health related entities not directly related to the usability of mHealth (Sheehan, Lee, Rodriguez, Tiase, & Schnall, 2012) (Sparkes, Valaitis, & McKibbon, 2012).

Breach of confidentiality may occur following receipt of text messages. In an mHealth study among diabetics in Ethiopia, willingness to receive text messages for appointment reminders was 70.5%, (Jemere et al., 2019). In North West Ethiopia, willingness to receive text message medication reminders among PLWH was only 50.9%, perhaps because use of SMS can't guarantee privacy and participants may have felt it was not safe to receive the mHealth support (Kebede, Zeleke, Asemahagn, & Fritz, 2015). In the rural United States, a survey carried out among 24 respondents, showed a demand for mHealth services, out of the 24 respondents, 63% were in good health, a total of 65% were willing to receive prerecorded messages for appointment reminders

MAKERERE UNIVERSITY
SCHOOL OF MEDICINE
APPROVED
VALID UNTIL
10 FEB 2021
RESEARCH & ETHICS COMMITTEE
P.O.BOX 7072, KAMPALA

from the doctor (Sankaranarayanan & Sallach, 2014). In rural India, willingness to receive mobile phone reminders for drug adherence was high (98%) among the 479 respondents. Of these, 424 (89%) preferred voice calls alone to other forms of communication (DeSouza, Rashmi, Vasanthi, Joseph, & Rodrigues, 2014).

In a rural Ugandan setting, a cross-sectional study found that over 60% of patients could potentially benefit from a mobile phone-based ART adherence support (Kim et al., 2015). Most of the mHealth studies conducted are SMS studies and only one used IVR. A qualitative study among youth assessed SMS based intervention to improve ART adherence in 2 clinics in Kampala, Uganda. Results suggested improved adherence could be achieved through reminders and social support. However, youth also suggested "intervention logistics related to content, frequency, timing and two-way messages" would be helpful to practitioners in the field (Rana et al., 2015). Another qualitative study in South–Western Uganda nested within sixty-two pilot intervention study participants found that SMS reminders and real-time adherence monitoring led to the habit of adherence, and real-time monitoring was interpreted as "being seen", "being cared about" which transformed their moods (Ware et al., 2016). In rural Uganda cohort of 276 participants on ART, more than half (64%) possessed a mobile phone and were willing to be contacted after a missed clinic appointment. In 79% of episodes of missed visits, patients presented for refills within a mean duration of 2.2 days (SD = 1.2 days) after mobile phone call reminder (Kunutsor et al., 2010). Another cross-sectional survey among PLWH in rural Uganda assessed the "acceptability of and preferences for text messaging, preferences for laboratory results notification, privacy and confidentiality and cell phone use and literacy". All the participants expressed interest in the service although they had challenges regarding "interpreting messages, discouragement upon learning bad news, and technical issues" (Siedner, Haberer, Bwana, Ware, & Bangsberg, 2012). In a study of the feasibility, validity, and acceptability of real-time adherence monitoring using a Wise pill wireless electronic adherence monitor (EAM) and self-reported missed doses via interactive voice response (IVR) and short message service (SMS)" among 49 adults and 46 children in Mbarara, Uganda, adherence was higher in children than adults (92.8% vs 89.5%) by EAM, and 99–100% for both adults and children by IVR/SMS self-report (Jessica E. Haberer et al., 2013).

MAKERERE UNIVERSITY
SCHOOL OF MEDICINE
APPROVED
VALID UNTIL
10 FEB 2021
RESEARCH & ETHICS COMMITTEE
P.O.BOX 7072, KAMPALA

2.3 mHealth and ART adherence virological outcomes

Poor adherence to antiretroviral treatment is a public health challenge associated with development of drug resistant strains necessitating initiation of costly second line therapy, and possibility of transmission of drug-resistant HIV by the non-adherent spouse (Chesney, Morin, & Sherr, 2000; De Costa et al., 2010; Paraskevis et al., 2017).

A quasi-experimental cohort study involving 150 HIV-infected individuals from Bangalore, India, showed that among participants on antiretroviral therapy (ART) between April and July 2010, optimal adherence increased from 85% to 91% patients during the intervention period, an effect that was maintained 6 months after the intervention was discontinued ($p=0.016$). Both, IVR calls and SMS reminders were considered nonintrusive and not a threat to privacy. A significantly higher proportion agreed that the IVR was helpful compared to the SMS ($p<0.001$) (Rodrigues et al., 2012).

A systematic review done by Ridgeway K et al, of the peer-reviewed and grey literature published between 2010 and 2015 to identify interventions designed to improve antiretroviral adherence among adults and adolescents in low and middle-income countries, found 43 interventions for adults, 6 involved both adults and adolescents and only 2 were among adolescents (Ridgeway et al., 2018). Two systematic reviews (Caroline Free, 2013; Free et al., 2013) found modest evidence for the benefits of mHealth technology. Both reviews recommended mHealth implementation, but argued that high quality (and adequately powered) clinical trials that measure clinical outcomes are essential (Tomlinson & Rotheram-Borus, 2013).

Garofalo *et al*, May 2016, conducted a randomized trial of a two-way, personalized daily text messaging intervention to improve adherence to antiretroviral therapy (ART) among 105 poorly adherent HIV-positive adolescents and youth, ages 16–29. The average effect estimate over the 6-month intervention period was significant for ≥ 90% adherence (OR=2.12, 95% CI=1.01–4.45, $p<.05$) and maintained at 12-months (6-months post-intervention) (Garofalo et al., 2016).

Patient-centered approaches exploiting mobile phone communications (mHealth) have been shown to improve adherence to cART and promote achievement of suppressed HIV plasma viral loads(A. R. Campbell et al., 2018a; J. I. Campbell & Haberer, 2015). A systematic review by H. Anglada-Martine et al, revealed that the use of text messages or mobile applications to enhance adherence to medication do seem to have been beneficial, as 65% of the studies evaluated had

MAKERERE UNIVERSITY
SCHOOL OF MEDICINE
APPROVED
VALID UNTIL
10 FEB 2021
RESEARCH & ETHICS COMMITTEE
P.O.BOX 7072, KAMPALA

positive outcomes. However, they recommended that more high-quality studies be conducted in order to demonstrate whether this type of technology reduces the considerable costs to the health system generated by non-adherence (Anglada-Mart et al., 2017).

During the WelTel Kenya 1 SMS trial, participants who received SMS support had significantly improved ART adherence and rates of viral suppression compared with control participants (Lester et al., 2010). In rural Uganda, text messaging was effective with regard to increasing ART adherence among adults, and this study aims to explore this effect among adolescents with poor virological suppression (A. Musiimenta et al., 2018).

Medication adherence is the "Plus" in the 90-90-90-Plus global challenge, emphasizing the importance of ART adherence in achieving viral suppression. Many gaps remain in meeting these goals, adherence self-efficacy, depression, stressful life events, and perceived stigma were significant predictors of medication adherence (Corless et al., 2017). Mobile phone applications that support treatment adherence address forgetfulness, knowledge gap; an important barrier to treatment adherence, and this needs to be explored among the youths in Uganda.

2.4 mHealth and retention in care

Predictors of poor retention in HIV care include younger age, male sex, racial or ethnic minority status, low socioeconomic status, no usual source of health care, less advanced HIV disease, fewer non-HIV-related comorbidities, and greater unmet psychosocial needs (Giordano, Hartman, Gifford, Backus, & Morgan, 2009). Thus, interventions to improve retention in care should incorporate informational, motivational, and behavioral skill components (Giordano, 2011).

Retaining individuals who have initiated ART in care is challenging. A review by Fox and Rosen 2010, estimated that 20% of patients in resource-limited settings are lost to follow-up within 12 months of initiating therapy (Fox & Rosen, 2010). Prior the era of "test and treat", the ART naïve "ART-ineligible" patients' clinic retention rate was estimate at 63% at month 12 (Kohler et al., 2011). There are many challenges for continuity of care, financial constraints, travel difficulties and stigma are factors that can prevent PLWH who start treatment from continuing in care (Mebal Namwabira, 2011).

Many people newly diagnosed with HIV are lost to follow-up shortly before or after timely initiation of antiretroviral therapy. One study in Kenya documented that people living with HIV

MAKERERE UNIVERSITY
SCHOOL OF MEDICINE
APPROVED
VALID UNTIL
10 FEB 2021
RESEARCH & ETHICS COMMITTEE
P.O.BOX 7072, KAMPALA

felt that increased communication via the text messaging intervention had the potential to enable early identification of problems, leading to timely problem solving that could improve retention and engagement in care during the first year after diagnosis. Engaging PLWH at all points along the cascade of care is essential for transforming the management of the disease from a fatal condition to a chronic one (Edward M. Gardner, McLees, Steiner, Del Rio, & Burman, 2011; Smillie et al., 2014).

In a recently published community-based individual randomized trial in Uganda, no statistically significant differences in retention were observed in the first 6 months between those randomized to either mobile-phone or physical contact tracing reminders. Retention was higher among those who received physical contact reminders at month 12 (91.5% versus 82.1%; p=X) (Kiwanuka et al., 2018). Although physical contact tracing reminders had higher retention at 12 and 18 months, mobile technology has several advantages which may be cost saving. The challenges of physical contact included structural barriers, time consuming, health care worker exhaustion and physical infrastructure challenges.

Various studies in Uganda have shown that patient-related factors resulting in loss to follow-up include transportation costs, distance to health centers, inconsistent income and lack of social support. Financing of transport improved retention, but this option is not cost effective nor sustainable in resource poor countries (Emenyonu et al., 2010; Kasigeire, 2014; Tuller et al., 2010).

Mobile health interventions may improve engagement in care. Mobile apps provide a convenient vehicle for reaching a large audience and offer the potential to create personalized and interactive interventions that can be used anonymously and discretely. In a study evaluating how youth use technology and mobile apps and how their use of technology could support their health and influence their engagement in care, participants suggested that a mobile health application should have the ability to connect to a community of other youth living with HIV, readily access healthcare providers, track personal data and information (such as laboratory data), and obtain health news and education (Dillingham et al., 2018).

2.5 mHealth technologies costs

Information systems, such as electronic health records (EHRs) and mobile phones and handheld computers (also called mHealth), can be of enormous value in providing health care in multiple settings. A review done in 2011 showed that with the exception of personal digital

MAKERERE UNIVERSITY
SCHOOL OF MEDICINE
APPROVED
VALID UNTIL
10 FEB 2021
RESEARCH & ETHICS COMMITTEE
P.O.BOX 7072, KAMPALA

assistant (PDA)-based data collection, there are still few scientifically rigorous data on the effectiveness and cost-effectiveness of e-health systems in developing countries (Blaya, Fraser, & Holt, 2010). The World Health Organization (WHO) published a manual on implementing EHRs for developing countries. The manual states that a thorough cost-benefit analysis should be conducted to compare the options against each other and the costs of any proposed system against the perceived benefits, so as to determine the value of the system to institution/ government (WHO, 2006).

A systematic review of peer-reviewed mHealth literature in Africa published between 2003 and 2013 identified 44 studies on mHealth and found that mHealth interventions were associated with positive health-related outcomes. Their success was based on the accessibility, acceptance and low-cost of the technology, effective adaptation to local contexts, strong stakeholder collaboration, and government involvement. In this review, it was clearly noted that threats such as dependency on funding, unclear healthcare system responsibilities, unreliable infrastructure and lack of evidence on cost-effectiveness challenge mHealth implementation (Blaya et al., 2010).

2.6 Hypotheses and Research Questions

1. What is the acceptability of mHealth among YLWH at Kiryandongo HIV Clinic, and what are the barriers, facilitators/enablers of ART and clinic visit adherence?

   Hypothesis: YLWH will have a high acceptability for mHealth with 70% accepting to use mHealth.

2. What is the effect of the mHealth-CFLU tool on ART adherence among YLWH?

   Hypothesis: More than 79% of YLWH using the mHealth-CFLU tool will be adherent to ART (using viral load suppression as a proxy measure for ART adherence we anticipate an increment of 15%).

3. What is the cost of the mHealth CFLU intervention compared to standard of care over a12 month period?

   Hypothesis: mHealth will be cost effective by increasing proportion in care and proportion with viral suppression compared to standard of care.

MAKERERE UNIVERSITY
SCHOOL OF MEDICINE
APPROVED
VALID UNTIL
10 FEB 2021
RESEARCH & ETHICS COMMITTEE
P.O.BOX 7072, KAMPALA

CHAPTER THREE: METHODS

3.1 Study Design: Mixed Methods Sequential study

The study design is a mixed method sequential, where the qualitative study will be done first, followed by the randomized controlled trial and thereafter the cost evaluation study.

3.2 Study setting

The study will be based at the HIV care and treatment clinic at Kiryandongo Hospital. Kiryandongo is located 225 km on the Kampala –Gulu highway. The hospital has a bed capacity of 109 and serves a population of 440,000 people. Kiryandongo hospital has been chosen as the study site, because it is an IDI implementing site with >1000 YLWH in care and a viral suppression rate of 79% below the UNAIDS target of 90% (Table 1). In 2018, a total of 637 youths were enrolled in care and retention in care was 79%. The current total of YLWH at the site is 1,322.

3.3 Study population

The study population will comprise of YLWH aged 15 -24 years who are ART naïve or on ART for ≤ 3months.

Target population:

Youth living with HIV registered to receive ART at Kiryandongo Hospital

Accessible population:

Youths consented/assented for CFLU, registered for care at Kiryandongo Hospital

3.4 Materials and Methods

Study Objectives:

1a: To assess barriers, enablers of adherence among youth living with HIV in Kiryandongo at baseline and study end.

1b: To assess acceptability of mHealth among youth living with HIV in Kiryandongo at baseline and study end.

MAKERERE UNIVERSITY
SCHOOL OF MEDICINE
APPROVED
VALID UNTIL
10 FEB 2021
RESEARCH & ETHICS COMMITTEE
P.O.BOX 7072, KAMPALA

Study design: Qualitative study with narrative approach

Study purpose: To identify the key behavioral constructs that enable or hinder ART adherence among the youths, and to assess acceptability of mHealth interventions for adherence support.

Study population: YLWH 15-24 years attending Kiryandongo Hospital HIV/ART clinic, ART naïve or on ART since Jun 2019.

Sampling: Participants will be purposively selected, each group will include 6-10 YWLH, and there will be a total of 3-5 groups ((Morgan, 1992). The participant groups will comprise of those who are naïve or initiating and on ART since Jun 2019. YWLH will be stratified by age group (≤18ys and >18yrs) and gender.

Study Procedure:

Selection criteria:

Inclusion: Youth living with HIV, with a documented HIV test result, aged 15-24 yrs, ART naïve or on ART since Jun 2019.

Exclusion Criteria: Youth unable to express themselves verbally, those unable to attend 2 sessions of the discussion

Selection of participants: We shall liaise with the clinic counsellor to refer YLWH aged 15-24 years with a documented HIV test result, patients who are ART naïve or initiating ART and have gone through routine counselling and testing. Stratification by age group and gender will be considered in selection of study participants. Eligible youth will be identified by the clinic counsellor and will be referred to study team which will be located at the HIV clinic. We shall sit one on one with the potential participant, give information about the purpose of the study, and request them to join the focus group discussion on the scheduled date if they agree to take part. This procedure will be repeated until the sample size of a particular group is reached.

Sample Size: Focus group discussions will be held until data saturation is achieved.

Data Collection:

MAKERERE UNIVERSITY
SCHOOL OF MEDICINE
APPROVED
VALID UNTIL
10 FEB 2021
RESEARCH & ETHICS COMMITTEE
P.O.BOX 7072, KAMPALA

Focus group discussions: On the day of the discussion, study information will be given to the participant including the purpose of the study, risks and benefits. We will address any questions they may have and obtain consent. An experienced person (Social Sciences group at the Academy) in conducting FGDs will facilitate the discussion assisted by two note takers (the PI and a research assistant). Ground rules will be set and choice of languages to use will be Runyoro, Kiswahili and English which are the dominant languages at the site. FGDs have been preferred and not in-depth interviews, because this study is generating information on collective views which will be used for later , and we want to generate a rich understanding of the youths experiences and beliefs (Gill, Stewart, Treasure, & Chadwick, 2008).

At baseline, we will administer an interview guide consisting of structured questions on the CFLU mHealth tool and ART adherence, as well as open-ended qualitative questions with probes, to allow exploration of YLWH views and opinions regarding these themes. The key issues to be explored are whether the youth would accept or refuse use of an mHealth tool, what they would/ wouldn't like the system to offer in terms of health education, frequency and timing of pill reminders and why. Regarding ART adherence, ART naïve participants will be asked what factors will likely facilitate / hinder their adherence to clinic attendance and ART. ART exposed participants will be asked what has facilitated or hindered their clinic attendance and ART adherence. The FGD will last for about 40 to 45 minutes. It will be audio recorded and refreshments (light snack and a soft drink) will be served. Themes to be assessed at baseline include acceptability of mHealth, assessing knowledge, usage, specifics needed, frequency timing.

At the end of the study, the groups will be subdivided according to the themes of acceptability and adherence. For acceptability, further segmentation will be based on those who have utilized the mHealth tool above or below 75% of times. For ART adherence, we shall subgroup according to viral suppression (VL <1000 or >1000 copies/mL), gender and age-group below or above 18 years. The FGD guide (appendix IV) will explore the different barriers and enablers for adherence, and the responses to mHealth intervention. The discussion will be captured on an audio recorder and handwritten notes.

Data collection for the FGD at study entry, before randomisation:

MAKERERE UNIVERSITY
SCHOOL OF MEDICINE
APPROVED
VALID UNTIL
10 FEB 2021
RESEARCH & ETHICS COMMITTEE
P.O.BOX 7072, KAMPALA

Themes	ART experienced (on ART 3months and above)		Less experienced (less than 3months) and ART Naive	
	Male		Female	
	≤18 yrs	>18yrs	≤18 yrs	> 18yrs
**Acceptability:**<br><br>Why accept or refuse mHealth<br><br>How can the system be used for adherence (probe on time, frequency)?<br><br>What they would like the system to do for them(Probe on pill reminders, Health tips/advice , symptom reporting and management and use of secret pin)				
**Adherence:**<br><br>Enablers/barriers:<br><br>(what has facilitated/hindered for those who are ART experienced)<br><br>(what will likely facilitate- for those who are naïve or less experienced)				

MAKERERE UNIVERSITY
SCHOOL OF MEDICINE
APPROVED
VALID UNTIL
10 FEB 2021
RESEARCH & ETHICS COMMITTEE
P.O.BOX 7072, KAMPALA

Data collection for the FGD at study end; groups will be divided according to how well they interacted with mHealth tool (> 50% ≤50%), age group (>18yrs ≤18yrs) and sex (male or female)

Themes	Groups			
	≤50% interaction/engagement with tool		(>50% interaction/engagement with tool)	
	Male		Female	
	≤18yrs	> 18yrs	≤18yrs	> 18yrs
**Acceptability:**<br>Comfort/ease using the tool<br>Following advice from Health tips<br>Likes/dislikes for tool<br>Improvements & recommendations				
**Adherence:**<br>Enablers (what has facilitated)<br>Barriers(What has hindered)				

MAKERERE UNIVERSITY
SCHOOL OF MEDICINE
APPROVED
VALID UNTIL
10 FEB 2021
RESEARCH & ETHICS COMMITTEE
P.O.BOX 7072, KAMPALA

Data Management and analysis:

The FGD audio-recordings will be translated and transcribed into English by a group of 4 qualitative research assistants from the Academy at Infectious Diseases Institute. Transcripts will be read, to identify themes and subthemes. Themes will be discussed, coded and the coded book with the transcripts will be imported to Nvivo for coding and content analysis. The analysis will be done by the qualitative social sciences Lead from the Academy at IDI. Audio files will be electronically stored in password protected files which will only be accessible to the study team. The analysis will be guided by the themes already contained in the interview guide, which will further be refined following multiple readings of interview scripts to better understand the data, identify sub-themes and to group the data according to themes for analysis and interpretation. Quotations reflecting the youths' views, opinions and experiences will be identified and will be used in the presentation of study findings.

Study Objective 2:

To assess effect of mHealth tool (CFLU) on ART adherence and knowledge of COVID-19 among YLWH naïve, initiating ART or on ART since Jun 2019.

Study design: Randomized control trial with 2 arms (Standard of Care or CFLU with Standard of Care)

Study purpose: To assess effect of CFLU on ART adherence among youth measured by interactive voice response to daily adherence calls mapped in the database and proportion with viral suppression of copies below 1000 copies/mL.

The intervention (CFLU) uses IVR calls or text messages delivered via MOTECH™ based Connect for Life technology™. The calls are delivered in 3 languages (Luganda, English and Runyakitara) and the participant has to make a choice of the preferred language during registration to the system. The system offers adherence pill reminders, health message tips, visit appointment reminders and receipt of self-reported symptoms.

A poor understanding of the disease among youths can result in delayed identification and treatment leading to rapid spread of infections among the community.

Study setting: Kiryandongo hospital HIV-ART clinic. Kiryandongo hospital has been chosen as the study site, because it has 1,322 YLWH and a viral suppression rate of 79% (Table 1). In 2018,

MAKERERE UNIVERSITY
SCHOOL OF MEDICINE
APPROVED
VALID UNTIL
10 FEB 2021
RESEARCH & ETHICS COMMITTEE
P.O.BOX 7072, KAMPALA

a total of 637 YLWH were enrolled in care. By Jun 2019 and additional 218 YLWH had been enrolled in care. The current total at the site for age between 15-24 is 1,322. Overall retention was 79%.

Study Population: Inclusion: YLWH (15-24 years), ART naïve or initiating ART since Jun 2019.

Exclusion: Hearing loss, severe illness likely to shorten life span, boarding school YLWH, Inability to use a basic mobile phone.

Sample size calculation:

Sample Size: X-Sectional, Cohort, & Randomized Clinical Trials (Kelsey et al., Methods in Observational Epidemiology 2nd Edition, Table 12-15; Fleiss, Statistical Methods for Rates and Proportions, formulas 3.18 &3.19)

The sample size formula for the method described in Kelsey et. al. is:

$$n_1 = \frac{\left(Z_{\alpha/2} + Z_{1-\beta}\right)^2 \bar{p}\bar{q}(r+1)}{r(p_1 - p_2)^2}$$

and

$n_2 = rn_1$

where

n_1 =number of cases in the intervention

MAKERERE UNIVERSITY
SCHOOL OF MEDICINE
APPROVED
VALID UNTIL
10 FEB 2021
RESEARCH & ETHICS COMMITTEE
P.O.BOX 7072, KAMPALA

n_2=number of controls

$Z_{\alpha/2}$ = standard normal deviate for two-tailed test based on alpha level (relates to the confidence interval level)

Z_{β} = standard normal deviate for one-tailed test based on beta level (relates to the power level)

r = ratio of controls to cases

p1 = proportion of cases with exposure and q1 = 1-p1

p2 = proportion of controls with exposure and q2 = 1-p2

$$\bar{p} = \frac{p_1 + rp_2}{r+1} \quad \text{and} \quad \bar{q} = 1 - \bar{p}$$

The sample size formula *without* the correction factor by Fleiss is:

MAKERERE UNIVERSITY
SCHOOL OF MEDICINE
APPROVED
VALID UNTIL
10 FEB 2021
RESEARCH & ETHICS COMMITTEE
P.O.BOX 7072, KAMPALA

$$n_{1cc} = \frac{n_1}{4}\left[1 + \sqrt{1 + \frac{2(r+1)}{n_1 r |p_2 - p_1|}}\right]$$

$$n_{2cc} = r\, n_{1cc}$$

When the input is provided as an odds ratio (OR) rather than the proportion of cases exposed, the proportion of cases exposed is calculated as:

$$p_1 = \frac{p_2 OR}{1 + p_2(OR - 1)}$$

Sample Size: X-Sectional, Cohort, & Randomized Clinical Trials (OpenEpi, 2019) (https://www.openepi.com/SampleSize/SSCohort.htm)

Two-sided significance level (1- α):	95
Power (1-β, % chance of detecting):	80
Ratio of sample size, Unexposed/Exposed:	1
Percent of Unexposed with Outcome:	79
Percent of Exposed with Outcome:	94(Jessica E. Haberer et al., 2013)
Odds Ratio:	4.2
Prevalence Ratio:	1.2
Prevalence difference:	15

	**Kelsey**	**Fleiss**	**Fleiss with CC**

Sample size- Exposed(intervention):	81	80	93
Sample Size-Nonexposed (Control):	81	80	93
Total Sample Size:	162	160	**186**

CC= continuity correction. Results are rounded up to the nearest integer.

Calculation output:

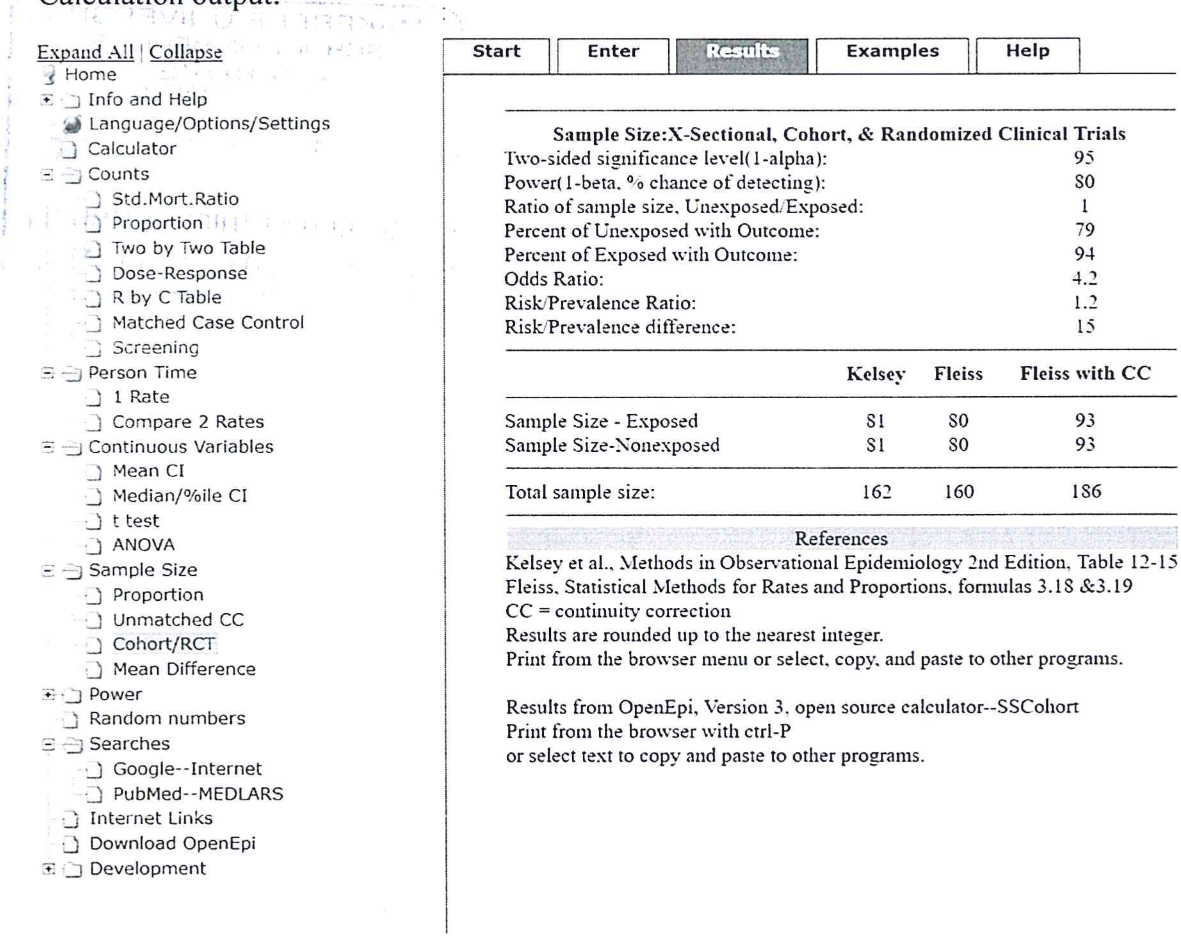

Sample Size:X-Sectional, Cohort, & Randomized Clinical Trials

Two-sided significance level(1-alpha):	95
Power(1-beta, % chance of detecting):	80
Ratio of sample size, Unexposed/Exposed:	1
Percent of Unexposed with Outcome:	79
Percent of Exposed with Outcome:	94
Odds Ratio:	4.2
Risk/Prevalence Ratio:	1.2
Risk/Prevalence difference:	15

	Kelsey	Fleiss	Fleiss with CC
Sample Size - Exposed	81	80	93
Sample Size-Nonexposed	81	80	93
Total sample size:	162	160	186

References

Kelsey et al., Methods in Observational Epidemiology 2nd Edition, Table 12-15
Fleiss, Statistical Methods for Rates and Proportions, formulas 3.18 &3.19
CC = continuity correction
Results are rounded up to the nearest integer.
Print from the browser menu or select, copy, and paste to other programs.

Results from OpenEpi, Version 3, open source calculator--SSCohort
Print from the browser with ctrl-P
or select text to copy and paste to other programs.

Factoring 10% loss to follow-up, the total sample size will be 206(103 for each arm)

Data collection procedures:

The intervention is mobile phone based IVR. However, the CLFU trial found that, it was observed that a good number of participants (50 of 256) at the urban site were not enrolled due to absence of a basic mobile phone, or had malfunctioning phone keyboards (for Poster Exhibition at the 20th International Conference on AIDS and STIs in Africa (ICASA 2019). Therefore, we

MAKERERE UNIVERSITY
SCHOOL OF MEDICINE
APPROVED
VALID UNTIL
10 FEB 2021
RESEARCH & ETHICS COMMITTEE
P.O.BOX 7072, KAMPALA

shall provide a basic mobile phone to everyone in the study (SoC and intervention arm). To avoid stigma associated with a particular phone, we shall get 4 or more types of basic phones. Issues regarding simcard registration will be dealt with by the individuals.

Four study questionnaires will be administered at baseline and 6-monthly until the 12-month exit visit.

The questionnaires will capture:

- Sociodemographic and medical history
- Stigma scores
- Knowledge assessment
- Sexual behavior assessment
- Knowledge of COVID-19 (only at baseline)

MAKERERE UNIVERSITY
SCHOOL OF MEDICINE
APPROVED
VALID UNTIL
10 FEB 2021
RESEARCH & ETHICS COMMITTEE
P.O.BOX 7072, KAMPALA

The data will be entered using REDCap. Blood for viral load will be collected at baseline and at the 6 and 12-month visit.

Intervention: mHealth CFLU tool

The tool capitalises on a basic mobile phone's core utilities of voice and short messaging services. The system design, development, testing and evaluation was done by Janssen and the Grameen Foundation (http://motechsuite.org/index.php/implementations). In 2015, Janssen Global Public Health Research and Development, in close collaboration with the Infectious Diseases Institute Kampala (IDI), developed Call for Life Uganda (CFLU) tailored to the needs of PLHIV in Uganda. The CFLU system follows the Health Insurance Portability and Accountability Act (HIPAA) Privacy and Security rules. While the Privacy rules deals with electronic Protected Health Information, the security rule covers administrative, physical and technical safeguards to ensure confidentiality, integrity and security of electronic protected health information. Data transaction between patient and system is encrypted when it comes to human subjects. The system is password protected, and interaction of system and patient is personalised with a secret code. The system interacts with the patient through a basic mobile phone via a keypad and with the health worker through a web-based interface. The system has options to either use interactive voice response or short message service and the user has to make a choice, get a secret pin code which ensures privacy to enduser.

Screening-Enrolment procedures:

All potential participants will be written on the screening log, and a screening form completed, reasons for screen failure will be documented on the screening log. Following successful screening, the participant will be referred to the Research Assistant (RA) to receive a thorough explanation of the study so and informed consent is obtained. The RA then administers the stigma questionnaire along with the sociodemographic and medical information, , HIV knowledge and sexual behaviour questionnaires. A client locater form will be completed which will help in physical contact tracing, and the participant will be registered on the enrolment log.

Randomisation will follow (see details of randomisation procedure below) thereafter and the result in the sealed envelope will dictate whether the patient will be registered onto the Call for Life System for the intervention or continue with standard of care as per MoH HIV Management guidelines.

Registration onto the CFLU system: Refer to Standard operating procedures on registration. However, all patients will be registered onto the electronic and hard copy of the enrolment log and a next appointment date is given to them. Finally, the lab request forms are filled and the patient sent for a blood draw. The patient returns from the lab, receives their transport refund of UGX 20,000, with a basic mobile phone (only at baseline visit, to all participants) and is allowed to go home.

No intervention: Standard of care arm

The Ministry of Health developed a minimum healthcare services package for PLHIV to standardize the programming, implementation and delivery of integrated HIV services in Uganda

The standard of care arm is based on the Apr 2018, consolidated guidelines for prevention and treatment of HIV in Uganda (MoH, 2018), and will also follow the healthcare services package for PLHIV (U. MoH, September 2014).

The SOC arem will follow the services below:

- Care and support for people living with HIV, and the first line ART regimen is TDF/3TC/DTG
- First-Line Regimen for Adults and Adolescents aged above 10 Initiating ART:

MAKERERE UNIVERSITY
SCHOOL OF MEDICINE
APPROVED
VALID UNTIL
10 FEB 2021
RESEARCH & ETHICS COMMITTEE
P.O.BOX 7072, KAMPALA

Preferred First-Line Regimen: TDF+3TC+DTG All HIV-infected adults and adolescents aged 10 years and above should be initiated on tenofovir, lamivudine and dolutegravir (TDF+3TC+DTG) as a once-daily fixed dose combination. When to use TDF+3TC+EFV: Adults and adolescents aged 10 years and above should only be initiated on TDF+3TC+EFV if their weight does not allow for use of the currently available DTG formulations (containing 50mg). Note: DTG 50mg should only be given to adults and adolescents weighing 40kg and above. When the 10mg and 25mg formulations become available, DTG will be administered to children and adolescents weighing less than 40kg.

- When to use ABC+3TC+DTG:
  Adults and adolescents aged 10 years and above should only be initiated on ABC+3TC+DTG if TDF is contraindicated, including the following conditions:
  1. Kidney disease and estimated glomerular filtration rate (GFR) below 60 ml/min
  2. Adolescents below 35 kg of weight.
- Adherence Preparation, Monitoring, And Support:
  The study team will use the 5 As principles for chronic care as a guide to offer pre-ART adherence counselling and psychosocial support. These are Assess, Advise, Agree, Assist and Arrange. The youths will be followed-up every 6 months as per MoH guidelines.

The Adult Care and Treatment Package includes the following services:(U. MoH, September 2014)

1. HIV counseling and testing
2. HIV prevention services
3. Nutritional Counseling and support
4. Opportunistic infection screening and management
5. Screening and management of non-communicable diseases
6. Diagnostics and laboratory monitoring
7. Sexual and reproductive health
8. Counseling and Psychosocial support
9. ARV preparation, initiation and monitoring

MAKERERE UNIVERSITY
SCHOOL OF MEDICINE
APPROVED
VALID UNTIL
10 FEB 2021
RESEARCH & ETHICS COMMITTEE
P.O.BOX 7072, KAMPALA

10. Adherence and retention into care

11. Palliative care

12. Mental health

Study Schema

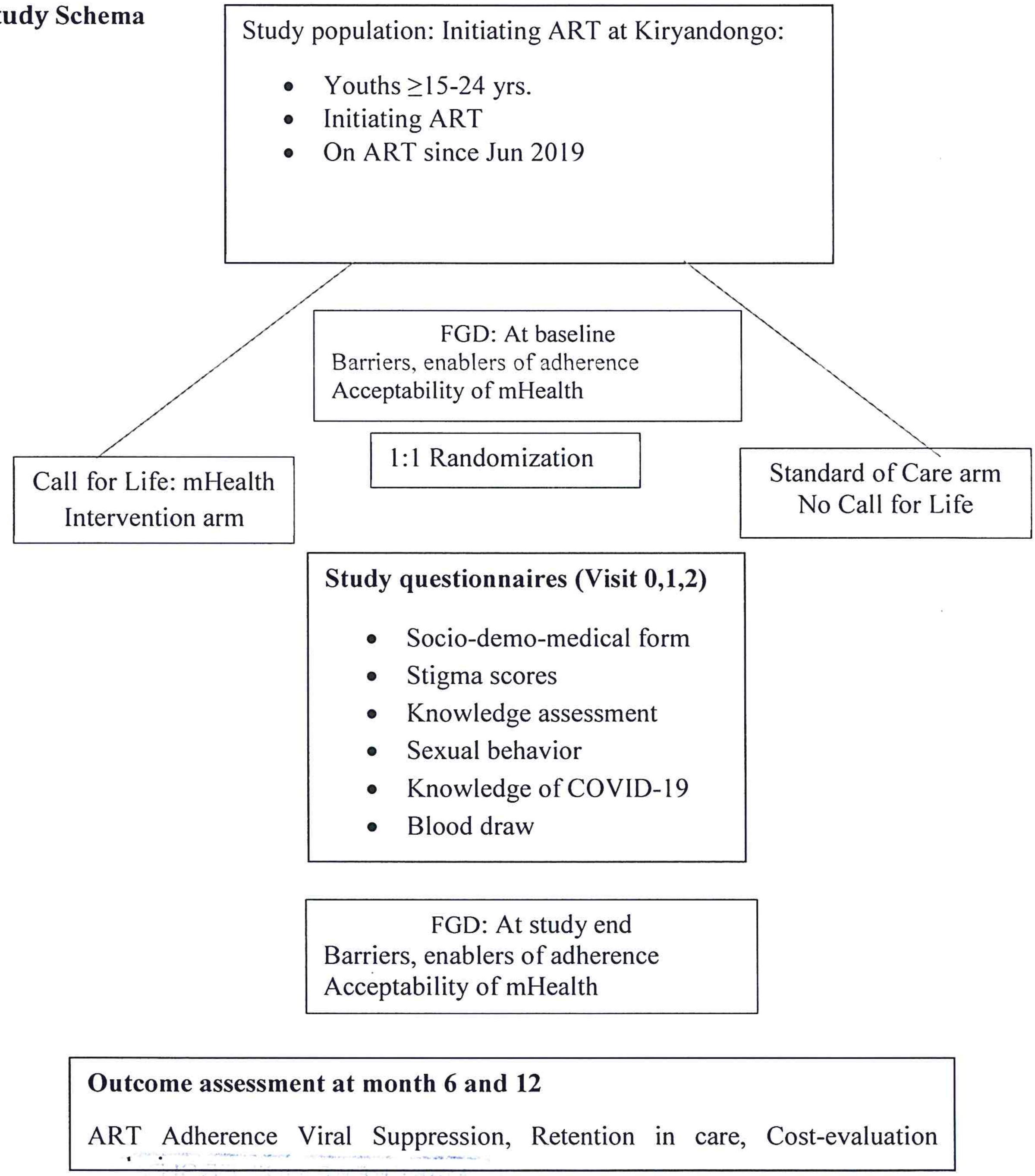

MAKERERE UNIVERSITY
SCHOOL OF MEDICINE
APPROVED
VALID UNTIL
10 FEB 2021
RESEARCH & ETHICS COMMITTEE
P.O.BOX 7072, KAMPALA

The outcome variables include:

- ART adherence scores at 6 and 12 months, as assessed by HIV Care Card for those on SoC arm and Self- reported adherence to ART as depicted on the mHealth dash board.
- Viral load suppression at 6 and 12 months, as test results from blood collected at months 6 and 12 with time to viral suppression.
- Retention rates at 6 and 12 months with time to loss to follow-up
- Stigma scores at study start and end

Data Management Analysis Plan

Data collected on paper CRF will be entered using Research Electronic Data Capture software (REDCap) which is a safe, secure, HIPAA-compliant, web-based application and facilitates offline and online data entry. Data will be exported to STATA 14.0, for analysis. Data will then be backed up and archived in both soft and hard copy to avoid losses. The data collected will be stored securely under lock and key to maintain patient confidentiality.

Data regarding call details in the system will be extracted from CFL tool database, exported into Stata for analysis.

Visit dates will be extracted from database and proportions lost to follow-up drawn and time to loss to follow-up estimated. Viral load results will be extracted and time to viral suppression with survival analysis curves drawn for both arms.

Data from the MoH HIV card regarding the ART regimen and start dates will be transcribed to the data collection tool and later entered on the REDCap system.

General data analysis

Data will be analyzed at three levels, first at the univariate level, then at the bivariate level and lastly at the multivariate level. A consort diagram will be drawn to show the screening, enrolment, randomization, follow-up and analysis flow of the study.

Univariate Analysis

MAKERERE UNIVERSITY
SCHOOL OF MEDICINE
APPROVED
VALID UNTIL
10 FEB 2021
RESEARCH & ETHICS COMMITTEE
P.O.BOX 7072, KAMPALA

Descriptive analysis will be done for the factors that influence adherence, virological suppression , stigma and retention in care. In addition, the distribution of the independent variables will be done with categorical data summarized using frequencies and percentages. Numerical data will be summarized using means and standard deviations for normally distributed data, and median and inter-quartile range for data that is not normally distributed.

Bivariate Analysis

The relationship between independent variables (age, gender, ART duration, marital status, education status, occupation and religion) and adherence and virological suppression will be analyzed using bivariate analysis made using cross tabular analysis and associations investigated using the Pearson Chi-square test.

The Chi-square test is based on the computational formulae below:

$$\chi^2 = \sum_{i=1}^{r}\sum_{j=1}^{c}\frac{\left(O_{ij}-E_{ij}\right)^2}{E_{ij}}$$

MAKERERE UNIVERSITY
SCHOOL OF MEDICINE
APPROVED
VALID UNTIL
10 FEB 2021
RESEARCH & ETHICS COMMITTEE
P.O.BOX 7072, KAMPALA

Where

r is the number of categories of the ith explanatory variable

c is the number of categories of the dependent variable

O_{ij} is the observed number of observations in the ith explanatory

and jth dependent variable

E_{ij} is the expected number of observations in the ith explanatory?

and j[th] dependent variable

Further, odds ratios shall be used to analyze the relationship between the dependent variable and the independent variables. Variables found to be significant at p value of $p \leq 0.05$ will considered for the multivariable analysis.

Multivariate Analysis

Multivariable logistic regression will be used to determine the factors that influence adherence or virological suppression and retention in care. Variables found to be statistically significant at $p \leq 0.05$ in bivariate analysis and those considered to have a scientific explanation will be entered into the multivariable model.

The multivariate logistic regression adopted is based on the formulae below:

$$\ln\left[\frac{p_i}{1 - p_i}\right] = \beta_0 + \beta_1 X_1 + \ldots + \beta_k X_k$$

Where;

X_i = independent variables

p_i = Probability of being adherent and viralogically suppression

$1 - p_i$ = Probability of not non-adherence or not suppressed virologically

β_0 = constant ; βi = coefficient of the determinant

The study findings will be reported as odds ratios, confidence interval and independent variables found to have p value ≤ 0.05 at 95% confidence interval will be considered to be statistically significant in the final model.

After the final model, significant factors for ART adherence/ viral suppression will be computed.

Data Monitoring Committee:

The DMC will consist of 3 independent members with expertise in relevant clinical specialties, their responsibility is to ensure that the safety of study subjects is protected while the scientific goals of the study are being met. The previous CFL-RCT at IDI, had no major safety issues and neither were there any gender-based violence, so the DMC will suffice and will be involved in:

- Review the research protocol and plan for data safety and monitoring.

MAKERERE UNIVERSITY
SCHOOL OF MEDICINE
APPROVED
VALID UNTIL
10 FEB 2021
RESEARCH & ETHICS COMMITTEE
P.O.BOX 7072, KAMPALA

- Evaluate the progress of the trial, including periodic assessments of data quality and timeliness, participant recruitment, accrual and retention, participant risk versus benefit, performance of the trial site, and other factors that can affect study outcome.

- Make recommendations to the PI about continuation, termination, or other modifications of the trial based on the observed beneficial or adverse effects of the intervention under study.

- If appropriate, conduct interim analysis of efficacy in accordance

- Recommend solutions to address problems with study conduct, enrolment, and sample size and/or data collection.

Study visits (scheduled and unscheduled visit codes)

The total duration of the study is 18 months; follow up period is up to 12 months. The participant has a baseline visit (00.0) and is followed up at month 6 (168 days ± 14 days) visit code 01.0, last visit is at month 12 (336 days ±14 days) visit code 02.0.

The participant might return at the clinic due to an illness, or repeat blood draw, this interim visit codes will be numbered sequentially from the previous scheduled visit. (00.01, 00.02 if its next to the baseline, if next to second visit, the code will be 01.01, 01.02 etc., if its next to last visit it will be coded 02.1, 02.2, etc.).

The study visit window is 2 weeks (14 days), beyond this period a visit code denoting an interim visit is given; e.g. Visit code 01.1 first contact with patient past visit 01.0 and 01.2 denotes second contact with patient past visit 01.0.

During enrolment, it is important to obtain the names and contact information for several individuals closely related to the participant (e.g., next of kin, friends, etc.). Such individuals can be contacted in the event that a participant does not return for follow-up visits. Physical contact tracing will happen if the patient takes 2 weeks past the scheduled visit.

Missed visit:

When the appointment is not honoured and the 14-day window passes, this is a missed visit. If a participant misses a visit (Visit window inclusive) a missed visit form is completed documenting the reason for missing (if known) and the attempts made to contact the participant, through mobile

MAKERERE UNIVERSITY
SCHOOL OF MEDICINE
APPROVED
VALID UNTIL
10 FEB 2021
RESEARCH & ETHICS COMMITTEE
P.O.BOX 7072, KAMPALA

phone or physical tracing. Several attempts to contact the participants must be made to ensure good retention. The attempts may include: Checking the General clinic schedule to sync the appointment visit, Calling the participant; Calling a relative who was disclosed to, or Visiting the participant's home: To minimise missed visits, through the web-interface on CFLU system, any dropped calls monitored on the dashboard, will trigger active follow-up and tracing of the participants.

Loss to follow-up:
If a participant misses 2 consecutive visits and unable to ascertain the vital status, this patient will be declared lost to follow-up, alternatively, if 3 months consecutively elapse, without acknowledging the patient's status, the patient will be labelled lost to follow-up. If a participant's where about is not known for 3 months and the contact tracing is uneventful after 3 documented attempts, this participant is deemed lost to follow-up and a withdraw form is completed. In case the participant re-surfaces to the clinic when the study is still active/ongoing, the withdraw form is cancelled out and patient re-joins the study and a study visit is conducted.

Study Objective 3:
To evaluate the cost of mHealth in comparison to Standard of Care

Study design: Cost evaluation analysis

Study Purpose: To assess cost of mHealth intervention in comparison to standard of care

Primary outcome measure: Costs spent on the intervention participants in comparison to standard of care participants
A cost analysis of the intervention from a governmental perspective will be performed. The principle of marginal costing will be used to cost the resources utilised in the alternative strategies. All costs of a historical nature, the sunk costs such as resources invested in buildings, equipment, and vehicles will be ignored. Only costs related to the future, from the onset of the study, will be recorded.

Sources of cost information: The following costs will be assessed and classified under direct or indirect cost: Sources of cost data will be; program/ project documents, financial records.

Capital costs:

I. System Costs:

MAKERERE UNIVERSITY
SCHOOL OF MEDICINE
APPROVED
VALID UNTIL
10 FEB 2021
RESEARCH & ETHICS COMMITTEE
P.O.BOX 7072, KAMPALA

- Software monthly hosting (internet, cloud hosting, call bills,)
- Support maintenance
- Hard ware
    - Computers
    - Internet modem

Recurrent costs:

I. Supplies (blood collection tubes, vacutainer holders, needle, cotton swabs)

II. Transport to fro (clinic visit):

III. Staff/ personnel salaries:

Induced costs:

I. Transport costs

II. Number of sick visits attended outside the clinic costs/fees incurred

III. Airtime fees

Health outcomes:

The number of patients with viral suppression (<1000 copies) will listed per study arm at month 6 and 12, and these will be compared across arms.

Analysis:

Primary outcome measure will be Cost for mHealth Intervention.

3.5 Quality control

All data collection tools will be checked for completeness and validated prior to entry on REDCap. Research assistant will follow the research protocol, will have prior protocol, Human subject protection (HSP) Good Clinical practice (GCP) course training, the consented youth randomized to CFL will be registered on a standalone CFL-PhD clinic for this study. The viral load results entered on the CFL tool will be cross-checked and verified by the PI, as the second data entrant. All patients consented for the study will get a basic phone to eliminate issues regarding phone technologies such as some buttons not functioning well. The study will be over seen by the DMC based at infectious Diseases Institute.

MAKERERE UNIVERSITY
SCHOOL OF MEDICINE
APPROVED
VALID UNTIL
10 FEB 2021
RESEARCH & ETHICS COMMITTEE
P.O.BOX 7072, KAMPALA

Data Security: All study data will be double encrypted, maintaining the confidentiality and safety of all data communications. The communication between the call for life browser and the server is encrypted using 128-bit SSL. System servers are hosted by Amazon Web Services (AWS) and are secured by Amazon VPC and AWS WAF firewalls to allow private networks and prevent unauthorized access, while data is protected from virus threats using BitDefender anti-virus technology. The YLWH database will be housed on the IDI server.

3.6 Ethical consideration

Permission to carry out the study will be sought from the Department of Medicine, School of Medicine, College of Health Sciences and ethical approval from the Higher Degrees Ethics and Research Committee of School of Medicine, Makerere University College of Health Sciences and research clearance from Uganda National Council for Science Technology. Clearance will also be sought from Kiryandongo Hospital administration and Kiryandongo District Health Office. The PI is Good Clinical Practice (GCP) compliant with a CITI certificate in GCP and Human Research Subject Protection (HSP). Informed consent and assent will be obtained and documented from all participants. Confidentiality will be maintained at all times. All data collected will be secured under lock and key. The CFLU is password protected and data is encrypted once extracted from database.

The National IT Authority policy will be followed and adhered to in relation to data protection, safety and confidentiality for patients.

Study limitations:

The intervention involves information technology and requirs reliable electricity supply, stable mobile phone network, and internet connectivity. Any disruption will lead to the CFLU system not operating as planned.

For the end user lack of experience with use of pin codes, multiple sim lines and failure to register simcard may lead to system failure Because entry of a wrong pin code will lead to blockage from use of the system. Use of multiple sim cards may lead to sim swapping and a call reminder may be received when the registered/authorised sim card number is not available because the other line is in use. Finally, unregistered simcards may be blocked by the Uganda Communications Commission.

Another limitation may be parental restrictions in phone use for YLWH enrolled in the study. The

MAKERERE UNIVERSITY
SCHOOL OF MEDICINE
APPROVED
VALID UNTIL
10 FEB 2021
RESEARCH & ETHICS COMMITTEE
P.O.BOX 7072, KAMPALA

study participant's response to daily pill reminders calls assumes that self-reporting is accurate. The Call for life health messages are in 4 main languages, due to lack of funds, the Luo language is missing and this may affect many potential participants.

MAKERERE UNIVERSITY
SCHOOL OF MEDICINE
APPROVED
VALID UNTIL
10 FEB 2021
RESEARCH & ETHICS COMMITTEE
P.O.BOX 7072, KAMPALA

UGANDA POPULATION-BASED HIV IMPACT ASSESSMENT (UPHIA 2016–2017)

VIRAL LOAD SUPPRESSION AMONG HIV-POSITIVE ADULTS, BY REGION

Among HIV-positive adults aged 15 to 64, prevalence of VLS varies geographically across the country, ranging from 48.8% in the East-Central region to 70.0% in the North-East region.

Region	VLS Prevalence (%)	95% CI
Central 1	64.2	55.7-72.6
Central 2	56.9	49.1-64.5
Kampala	62.1	53.4-70.7
East-Central	48.8	42.3-55.2
Mid-East	52.9	44.4-61.4
North-East	70.0	60.4-79.5
West Nile	60.5	50.5-70.5
Mid-North	54.6	45.4-63.7
Mid-West	55.3	46.1-64.4
South-West	68.0	60.2-75.7
Overall	59.6	56.8-62.5

The table can be found on: https://phia.icap.columbia.edu/wp-content/uploads/2019/07/UPHIA_Final_Report_Revise_07.11.2019_Final_for-web.pdf

MAKERERE UNIVERSITY
SCHOOL OF MEDICINE
APPROVED
VALID UNTIL
10 FEB 2021
RESEARCH & ETHICS COMMITTEE
P.O.BOX 7072, KAMPALA

TABLE 1: Viral suppresion among YLWH enrolled at Kiryandongo Hospital

Currently in Care

	TX_CURR Dec 2017			TX_CURR Dec18			TX_CURR Jun19		
	15-19Yrs	20-24Yrs	TOTAL	15-19Yrs	20-24Yrs	TOTAL	15-19Yrs	20-24Yrs	TOTAL
Total	413	761	1174	451	857	1308	506	816	1322

		New in Care							
	TX_NEW Jan to Dec 17			TX_NEW Jan to Dec 18			TX_NEW Jan to June 19		
	15-19Yrs	20-24Yrs	TOTAL	15-19Yrs	20-24Yrs	TOTAL	15-19Yrs	20-24Yrs	TOTAL
Total	64	232	296	148	489	637	49	169	218

Viral Suppression

Period	Valid Results	Suppressed Results	Non Suppressed Results	Suppression Rate (%)
Dec-17	414	298	116	72%
Dec-18	466	368	98	79%
Jun-19	260	208	52	80%

Retention over all at the site

	Dec-17			Oct to Dec 2018				
	12 Months			12 Months				
**Organisation unit**	**Net Current Cohort**	**%age retained**		**Net Current Cohort**	**%age retained**			
TOTAL	282	76%		266	79%			

MAKERERE UNIVERSITY
SCHOOL OF MEDICINE
APPROVED
VALID UNTIL
10 FEB 2021
RESEARCH & ETHICS COMMITTEE
P.O.BOX 7072, KAMPALA

FIGURE1: CALL FOR LIFE UGANDA- FUNCTIONALITY MODULES (INTERVENTION TO BE USED)

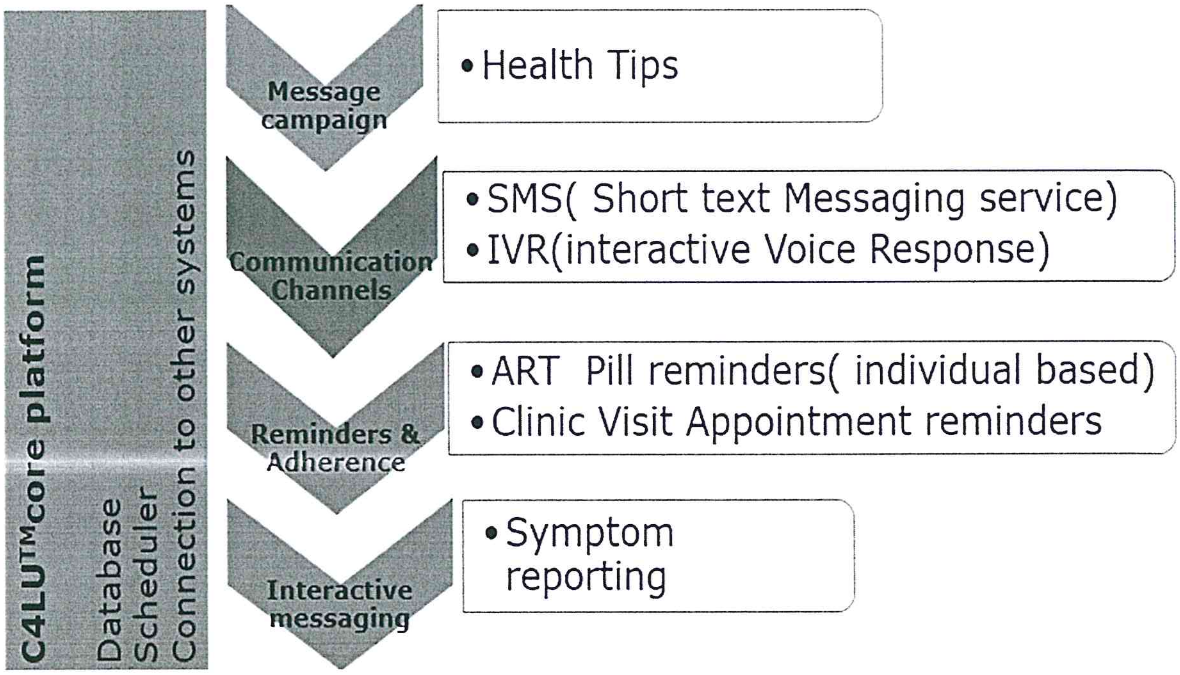

MAKERERE UNIVERSITY
SCHOOL OF MEDICINE
APPROVED
VALID UNTIL
10 FEB 2021
RESEARCH & ETHICS COMMITTEE
P.O.BOX 7072, KAMPALA

4. REFERENCES

https://www.hiv.gov/hiv-basics/overview/data-and-trends/global-statistics

Agwu, A. L., Bethel, J., Hightow-Weidman, L. B., Sleasman, J. W., Wilson, C. M., Rudy, B., . . . the Adolescent Medicine Trials Network for, H. I. V. A. I. (2012). Substantial multiclass transmitted drug resistance and drug-relevant polymorphisms among treatment-naïve behaviorally HIV-infected youth. *AIDS Patient Care STDS, 26*(4), 193-196. doi: 10.1089/apc.2011.0420

AIDSInfo. (2019). Young People (15-24yrs) living with HIV-by sex. 2019, from https://aidsinfo.unaids.org/

Amith, M., Loubser, P. G., Chapman, J., Zoker, K. C., & Rabelo Ferreira, F. E. R. (2012). Optimization of an EHR mobile application using the UFuRT conceptual framework. *AMIA ... Annual Symposium proceedings. AMIA Symposium, 2012*, 209-217.

Ammassari, A., Trotta, M. P., Shalev, N., Tettoni, M. C., Maschi, S., Di Sora, F., . . . Antinori, A. (2011). Timed Short Messaging Service Improves Adherence and Virological Outcomes in HIV-1–Infected Patients With Suboptimal Adherence to Antiretroviral Therapy. *JAIDS Journal of Acquired Immune Deficiency Syndromes, 58*(4), e113-e115. doi: 10.1097/QAI.0b013e3182359d2a

Ammassari, A. M., et al. (2011). Timed Short Messaging Service Improves Adherence and Virological Outcomes in HIV-1–Infected Patients With Suboptimal Adherence to Antiretroviral Therapy. *JAIDS, 58*(4), e113–e115. doi: 10.1097/QAI.0b013e3182359d2a

Anglada-Mart, H., #237, nez, Martin-Conde, M., Rovira-Illamola, M., Sotoca-Momblona, J. M., . . . #233. (2017). An Interactive Mobile Phone---Website Platform to Facilitate Real-Time Management of Medication in Chronically ill Patients. *J. Med. Syst., 41*(8), 1-8. doi: 10.1007/s10916-017-0767-7

Avert.Org. (2019, 21 Aug 2018). Young People HIV and AIDS. *Global information and education on HIV and AIDS.* Retrieved 7 Oct, 2019

Babalola Stella, e. a. (2017). A Framework for Health Communication Across the

HIV Treatment Continuum *Journal of Acquired Immune Deficiency Syndromes, 74*, S5-S14.

Blaya, J. A., Fraser, H. S., & Holt, B. (2010). E-health technologies show promise in developing countries. *Health Aff (Millwood), 29*(2), 244-251. doi: 10.1377/hlthaff.2009.0894

Burnay, J., Bushman, B. J., & Laroi, F. (2019). Effects of sexualized video games on online sexual harassment. *Aggress Behav, 45*(2), 214-223. doi: 10.1002/ab.21811

Campbell, A. R., Kinvig, K., Côté, H. C., Lester, R. T., Qiu, A. Q., Maan, E. J., . . . Murray, M. C. (2018a). Health Care Provider Utilization and Cost of an mHealth Intervention in Vulnerable People Living With HIV in Vancouver, Canada: Prospective Study. *JMIR mHealth and uHealth, 6*(7).

Campbell, A. R., Kinvig, K., Côté, H. C., Lester, R. T., Qiu, A. Q., Maan, E. J., . . . Murray, M. C. (2018b). Health Care Provider Utilization and Cost of an mHealth Intervention in Vulnerable People Living With HIV in Vancouver, Canada: Prospective Study. *JMIR mHealth and uHealth, 6*(7), e152.

Campbell, J. I., & Haberer, J. E. (2015). Cell Phone-Based and Adherence Device Technologies for HIV Care and Treatment in Resource-Limited Settings: Recent Advances. *Curr HIV/AIDS Rep, 12*(4), 523-531. doi: 10.1007/s11904-015-0282-8

Caroline Free, G. P., Louise Watson, et al. (2013). The effectiveness of mobile health technologies to improve health care service delivery processes. A systematic review and meta analysis. *PLoS Med, 10*(1), e1001363. doi: 10.1371/journal.pmed.1001363

CDC. (1998). Report of the NIH Panel to Define Principles of Therapy of HIV Infection and Guidelines for the Use of Antiretroviral Agents in HIV-Infected Adults and Adolescents (U. S. D. O. H. A. H. SERVICES & C. f. D. C. a. P. (CDC), Trans.) (Vol. 47): U.S. DEPARTMENT OF HEALTH AND HUMAN SERVICES

Centers for Disease Control and Prevention (CDC).

Chang, L. W., Kagaayi, J., Arem, H., Nakigozi, G., Ssempijja, V., Serwadda, D., . . . Reynolds, S. J. (2011). Impact of a mHealth intervention for peer health workers on AIDS care in rural Uganda: a mixed

MAKERERE UNIVERSITY
SCHOOL OF MEDICINE
APPROVED
VALID UNTIL
10 FEB 2021
RESEARCH & ETHICS COMMITTEE
P.O.BOX 7072, KAMPALA

methods evaluation of a cluster-randomized trial. *AIDS Behav, 15*(8), 1776-1784. doi: 10.1007/s10461-011-9995-x

Chesney, M. A., Morin, M., & Sherr, L. (2000). Adherence to HIV combination therapy. *Soc Sci Med, 50*(11), 1599-1605.

Chib, A., & Harris, R. *Linking Research to Practice: Strengthening ICT for Development Research Capacity in Asia*: ISEAS- Yusof Ishak Institute.

Corkrey, R., Parkinson, L., Bates, L., Green, S., & Htun, A. T. (2005). Pilot of a novel cervical screening intervention: interactive voice response. *Australian and New Zealand Journal of Public Health, 29*(3), 261-264. doi: 10.1111/j.1467-842X.2005.tb00765.x

Corless, I. B., Hoyt, A. J., Tyer-Viola, L., Sefcik, E., Kemppainen, J., Holzemer, W. L., . . . Nicholas, P. K. (2017). 90-90-90-Plus: Maintaining Adherence to Antiretroviral Therapies. *AIDS Patient Care STDS, 31*(5), 227-236. doi: 10.1089/apc.2017.0009

De Costa, A., Shet, A., Kumarasamy, N., Ashorn, P., Eriksson, B., Bogg, L., . . . team, H. s. (2010). Design of a randomized trial to evaluate the influence of mobile phone reminders on adherence to first line antiretroviral treatment in South India--the HIVIND study protocol. *BMC Med Res Methodol, 10*, 25. doi: 10.1186/1471-2288-10-25

DeSouza, S. I., Rashmi, M. R., Vasanthi, A. P., Joseph, S. M., & Rodrigues, R. (2014). Mobile phones: the next step towards healthcare delivery in rural India? *PloS one, 9*(8), e104895. doi: 10.1371/journal.pone.0104895

Dillingham, R., Ingersoll, K., Flickinger, T. E., Waldman, A. L., Grabowski, M., Laurence, C., . . . Cohn, W. F. (2018). PositiveLinks: A Mobile Health Intervention for Retention in HIV Care and Clinical Outcomes with 12-Month Follow-Up. *AIDS Patient Care STDS, 32*(6), 241-250. doi: 10.1089/apc.2017.0303

Doris Caliz, X. A. Usability Evaluation Method for Mobile Applications for the Elderly: A Methodologica Proposal.

Emenyonu, N., Muyindike, W., Habyarimana, J., Pops-Eleches, C., Thirumurthy, H., Ragland, K., & Bangsberg, D. (2010). *Cash transfers to cover clinic transportation costs improve adherence and retention in care in a HIV treatment program in rural Uganda.* Paper presented at the 17th Conference on retroviruses and opportunistic infections.

Fisher, W. A., Fisher, J. D., & Harman, J. (2003). The Information-Motivation-Behavioral Skills Model: A General Social Psychological Approach to Understanding and Promoting Health Behavior. 82-106. doi: 10.1002/9780470753552.ch4

Fox, M. P., & Rosen, S. (2010). Patient retention in antiretroviral therapy programs up to three years on treatment in sub-Saharan Africa, 2007-2009: systematic review. *Trop Med Int Health, 15 Suppl 1*, 1-15. doi: 10.1111/j.1365-3156.2010.02508.x

Free, C., Phillips, G., Galli, L., Watson, L., Felix, L., Edwards, P., . . . Haines, A. (2013). The effectiveness of mobile-health technology-based health behaviour change or disease management interventions for health care consumers: a systematic review. *PLoS Med, 10*(1), e1001362. doi: 10.1371/journal.pmed.1001362

Gardner, E. M., Burman, W. J., Steiner, J. F., Anderson, P. L., & Bangsberg, D. R. (2009). Antiretroviral medication adherence and the development of class-specific antiretroviral resistance. *AIDS (London, England), 23*(9), 1035.

Gardner, E. M., McLees, M. P., Steiner, J. F., Del Rio, C., & Burman, W. J. (2011). The spectrum of engagement in HIV care and its relevance to test-and-treat strategies for prevention of HIV infection. *Clin Infect Dis, 52*(6), 793-800. doi: 10.1093/cid/ciq243

Garofalo, R., Kuhns, L. M., Hotton, A., Johnson, A., Muldoon, A., & Rice, D. (2016). A Randomized Controlled Trial of Personalized Text Message Reminders to Promote Medication Adherence Among HIV-Positive Adolescents and Young Adults. *AIDS Behav, 20*(5), 1049-1059. doi: 10.1007/s10461-015-1192-x

MAKERERE UNIVERSITY
SCHOOL OF MEDICINE
APPROVED
VALID UNTIL
10 FEB 2021
RESEARCH & ETHICS COMMITTEE
P.O.BOX 7072, KAMPALA

Gill, P., Stewart, K., Treasure, E., & Chadwick, B. (2008). Methods of data collection in qualitative research: interviews and focus groups. *Br Dent J, 204*(6), 291-295. doi: 10.1038/bdj.2008.192

Giordano, T. P. (2011). Retention in HIV care: what the clinician needs to know. *Top Antivir Med, 19*(1), 12-16.

Giordano, T. P., Hartman, C., Gifford, A. L., Backus, L. I., & Morgan, R. O. (2009). Predictors of retention in HIV care among a national cohort of US veterans. *HIV Clin Trials, 10*(5), 299-305. doi: 10.1310/hct1005-299

Haberer, J. E., Kiwanuka, J., Nansera, D., Muzoora, C., Hunt, P. W., So, J., . . . Bangsberg, D. R. (2013). Realtime adherence monitoring of antiretroviral therapy among HIV-infected adults and children in rural Uganda. *AIDS (London, England), 27*(13), 2166-2168. doi: 10.1097/QAD.0b013e328363b53f

Haberer, J. E., Musiimenta, A., Atukunda, E. C., Musinguzi, N., Wyatt, M. A., Ware, N. C., & Bangsberg, D. R. (2016). Short message service (SMS) reminders and real-time adherence monitoring improve antiretroviral therapy adherence in rural Uganda. *AIDS, 30*(8), 1295-1300. doi: 10.1097/QAD.0000000000001021

Hawkins, A., Evangeli, M., Sturgeon, K., Le Prevost, M., Judd, A., & Committee, A. S. (2016). Episodic medication adherence in adolescents and young adults with perinatally acquired HIV: a within-participants approach. *AIDS Care, 28*(sup1), 68-75.

Hettema, J. E., Hosseinbor, S., & Ingersoll, K. S. (2012). Feasibility and Reliability of Interactive Voice Response Assessment of HIV Medication Adherence: Research and Clinical Implications. *HIV Clin Trials, 13*(5), 271-277. doi: 10.1310/hct1305-271

Hudelson, C., & Cluver, L. (2015). Factors associated with adherence to antiretroviral therapy among adolescents living with HIV/AIDS in low-and middle-income countries: a systematic review. *AIDS Care, 27*(7), 805-816.

Ippoliti, N. B., & L'Engle, K. (2017). Meet us on the phone: mobile phone programs for adolescent sexual and reproductive health in low-to-middle income countries. *Reproductive health, 14*(1), 11-11. doi: 10.1186/s12978-016-0276-z

Jacob , P. R., Oates. (2015). Cell-Phone in Africa: Communication LifelineAL-April-15-2015. *Pew-Research-Center-Africa.*

Jeffrey D.Fisher, W. A. F. (1992). <Changing AIDS-Risk Behavior>. *Psychological Bulletin, 111*(3), 455-474.

Jemere, A. T., Yeneneh, Y. E., Tilahun, B., Fritz, F., Alemu, S., & Kebede, M. (2019). Access to mobile phone and willingness to receive mHealth services among patients with diabetes in Northwest Ethiopia: a cross-sectional study. *BMJ Open, 9*(1), e021766-e021766. doi: 10.1136/bmjopen-2018-021766

Jones, D., Cook, R., Rodriguez, A., & Waldrop-Valverde, D. (2013). Personal HIV knowledge, appointment adherence and HIV outcomes. *AIDS Behav, 17*(1), 242-249. doi: 10.1007/s10461-012-0367-y

Kasigeire, S. (2014). Socio-Economic Conditions and retention of clients in HIV Care and Treatment at Kagadi Hospital, Uganda. *manuscript.*

Kebede, M., Zeleke, A., Asemahagn, M., & Fritz, F. (2015). Willingness to receive text message medication reminders among patients on antiretroviral treatment in North West Ethiopia: A cross-sectional study. *BMC Med Inform Decis Mak, 15*, 65-65. doi: 10.1186/s12911-015-0193-z

Kerstin E. E. Schroder, & Johnson, C. J. (2009). Interactive Voice Response Technology to Measure HIV-related Behavior. *Behavioral Aspects of HIV Management*, 210- 216.

Kim, J., Zhang, W., Nyonyitono, M., Lourenco, L., Nanfuka, M., Okoboi, S., . . . Moore, D. M. (2015). Feasibility and acceptability of mobile phone short message service as a support for patients receiving antiretroviral therapy in rural Uganda: a cross-sectional study. *Journal of the International AIDS Society, 18*(1), 20311-20311. doi: 10.7448/IAS.18.1.20311

Kiwanuka, N., Mpendo, J., Asiimwe, S., Ssempiira, J., Nalutaaya, A., Nambuusi, B., . . . Ssetaala, A. (2018). A randomized trial to assess retention rates using mobile phone reminders versus physical contact

MAKERERE UNIVERSITY
SCHOOL OF MEDICINE
APPROVED
VALID UNTIL
10 FEB 2021
RESEARCH & ETHICS COMMITTEE
P.O.BOX 7072, KAMPALA

tracing in a potential HIV vaccine efficacy population of fishing communities around Lake Victoria, Uganda. *BMC Infect Dis, 18*(1), 591. doi: 10.1186/s12879-018-3475-0

Kohler, P. K., Chung, M. H., McGrath, C. J., Benki-Nugent, S. F., Thiga, J. W., & John-Stewart, G. C. (2011). Implementation of free cotrimoxazole prophylaxis improves clinic retention among antiretroviral therapy-ineligible clients in Kenya. *AIDS (London, England), 25*(13), 1657-1661. doi: 10.1097/QAD.0b013e32834957fd

Koirala, S., Deuba, K., Nampaisan, O., Marrone, G., Ekström, A. M., & group, C.-S. (2017). Facilitators and barriers for retention in HIV care between testing and treatment in Asia—a study in Bangladesh, Indonesia, Lao, Nepal, Pakistan, Philippines and Vietnam. *PloS one, 12*(5), e0176914.

Kunutsor, S., Walley, J., Katabira, E., Muchuro, S., Balidawa, H., Namagala, E., & Ikoona, E. (2010). Using Mobile Phones to Improve Clinic Attendance Amongst an Antiretroviral Treatment Cohort in Rural Uganda: A Cross-sectional and Prospective Study. *AIDS Behav, 14*(6), 1347-1352. doi: 10.1007/s10461-010-9780-2

Lawrence Mbuagbaw, Lahana Thabane, Pierre Ongolo-Zogo, Richard T Lester, Edward Mills, Jimmy Volmink, . . . Ondoa, H. A. (2011). The cameroon mobile phone sms (CAMPS) trial: a protocol for a randomized controlled trial of mobile phone text messaging versus usual care for improving adherence to highly active anti-retroviral therapy *Trials, 12*(5).

Lee-Ann Crystal, D. (2017). *Exploration of adherence to antiretroviral treatment amongst adolescents in a low socio-economic urban setting in Cape Town, South Africa.* (Master of Public Health), University of the Western Cape.

Lester, R. T., Ritvo, P., Mills, E. J., Kariri, A., Karanja, S., Chung, M. H., . . . Plummer, F. A. (2010). Effects of a mobile phone short message service on antiretroviral treatment adherence in Kenya (WelTel Kenya1): a randomised trial. *The Lancet, 376*(9755), 1838-1845. doi: 10.1016/s0140-6736(10)61997-6

Luxton, D. D., June, J. D., & Chalker, S. A. (2015). Mobile Health Technologies for Suicide Prevention: Feature Review and Recommendations for Use in Clinical Care. *Current Treatment Options in Psychiatry, 2*(4), 349-362. doi: 10.1007/s40501-015-0057-2

Mebal Namwabira, E. K. N., Nigel Livesley, Alex R Ario,. (2011). Improving Retention of patients in HIV Care in Uganda. *Poster, internattional Conference*.

Miller, L. G., Liu, H., Hays, R. D., Golin, C. E., Ye, Z., Beck, C. K., . . . Wenger, N. S. (2003). Knowledge of antiretroviral regimen dosing and adherence: a longitudinal study. *Clin Infect Dis, 36*(4), 514-518. doi: 10.1086/367857

MoH. (2018). *CONSOLIDATED GUIDELINES FOR PREVENTION AND TREATMENT OF HIV IN UGANDA*.

MoH, U. (September 2014). *Intergrated Health Care Services Package for HIV Prevention, Treatment and Care Servicea for Uganda*.

Morgan, D. L. (1992). Designing focus group research *Tools for primary care research.* (pp. 177-193). Thousand Oaks, CA, US: Sage Publications, Inc.

Musiimenta, A., Atukunda, E. C., Tumuhimbise, W., Pisarski, E. E., Tam, M., Wyatt, M. A., . . . Haberer, J. E. (2018). Acceptability and Feasibility of Real-Time Antiretroviral Therapy Adherence Interventions in Rural Uganda: Mixed-Method Pilot Randomized Controlled Trial. *JMIR Mhealth Uhealth, 6*(5), e122. doi: 10.2196/mhealth.9031

Musiimenta, A., Atukunda, E. C., Tumuhimbise, W., Pisarski, E. E., Tam, M., Wyatt, M. A., . . . Haberer, J. E. (2018). Acceptability and feasibility of real-time antiretroviral therapy adherence interventions in rural Uganda: Mixed-method pilot randomized controlled trial. *JMIR mHealth and uHealth, 6*(5), e122.

Nabukeera-Barungi, N., Elyanu, P., Asire, B., Katureebe, C., Lukabwe, I., Namusoke, E., . . . Tumwesigye, N. (2015). Adherence to antiretroviral therapy and retention in care for adolescents living with HIV from 10 districts in Uganda. *BMC Infectious Diseases, 15*(1), 520. doi: 10.1186/s12879-015-1265-5

MAKERERE UNIVERSITY
SCHOOL OF MEDICINE
APPROVED
VALID UNTIL
10 FEB 2021
RESEARCH & ETHICS COMMITTEE
P.O.BOX 7072, KAMPALA

Nachega, J. B., Adetokunboh, O., Uthman, O. A., Knowlton, A. W., Altice, F. L., Schechter, M., . . . Mills, E. J. (2016). Community-Based Interventions to Improve and Sustain Antiretroviral Therapy Adherence, Retention in HIV Care and Clinical Outcomes in Low- and Middle-Income Countries for Achieving the UNAIDS 90-90-90 Targets. *Current HIV/AIDS reports, 13*(5), 241-255. doi: 10.1007/s11904-016-0325-9

Nasuuna, E., Kigozi, J., Babirye, L., Muganzi, A., Sewankambo, N. K., & Nakanjako, D. (2018). Low HIV viral suppression rates following the intensive adherence counseling (IAC) program for children and adolescents with viral failure in public health facilities in Uganda. *BMC Public Health, 18*(1), 1048. doi: 10.1186/s12889-018-5964-x

Natalie Leon, H. S. a. E. D. (2012). Applying a framework for assessing the health system challenges to scaling up mHealth in South Africa. *BMC Medical Informatics and Decision Making, 12*(123). doi: 10.1186/1472-6947-12-123

Olum, R., Chekwech, G., Wekha, G., Nassozi, D. R., & Bongomin, F. Coronavirus Disease-2019: Knowledge, Attitude, and Practices of Health Care Workers at Makerere University Teaching Hospitals, Uganda. (2296-2565 (Print)).

OpenEpi. (2019). Sample Size for Cross-Sectional, Cohort, & Randomized Clinical Trial Studies. Retrieved Oct 2, 2019, from https://www.openepi.com/SampleSize/SSCohort.htm

Oyugi, J. H., Byakika-Tusiime, J., Ragland, K., Laeyendecker, O., Mugerwa, R., Kityo, C., . . . Bangsberg, D. R. (2007). Treatment interruptions predict resistance in HIV-positive individuals purchasing fixed-dose combination antiretroviral therapy in Kampala, Uganda. *AIDS, 21*(8), 965-971. doi: 10.1097/QAD.0b013e32802e6bfa

Paraskevis, D., Kostaki, E., Gargalianos, P., Xylomenos, G., Lazanas, M., Chini, M., . . . Hatzakis, A. (2017). Transmission Dynamics of HIV-1 Drug Resistance among Treatment-Naive Individuals in Greece: The Added Value of Molecular Epidemiology to Public Health. *Genes (Basel), 8*(11). doi: 10.3390/genes8110322

Perrine, M. W., Mundt, J. C., Searles, J. S., & Lester, L. S. (1995). Validation of daily self-reported alcohol consumption using interactive voice response (IVR) technology. *Journal of Studies on Alcohol, 56*(5), 487-490. doi: 10.15288/jsa.1995.56.487

Qudrat-Ullah, H., & Tsasis, P. (2017). Innovative healthcare systems for the 21st century. from http://public.eblib.com/choice/publicfullrecord.aspx?p=5576718

Rana, Y., Haberer, J., Huang, H., Kambugu, A., Mukasa, B., Thirumurthy, H., . . . Linnemayr, S. (2015). Short Message Service (SMS)-Based Intervention to Improve Treatment Adherence among HIV-Positive Youth in Uganda: Focus Group Findings. *PloS one, 10*(4), e0125187. doi: 10.1371/journal.pone.0125187

Reidel, K., Tamblyn, R., Patel, V., & Huang, A. (2008). Pilot study of an interactive voice response system to improve medication refill compliance. *BMC Med Inform Decis Mak, 8*(1), 46. doi: 10.1186/1472-6947-8-46

Ridgeway, K., Dulli, L. S., Murray, K. R., Silverstein, H., Dal Santo, L., Olsen, P., . . . McCarraher, D. R. (2018). Interventions to improve antiretroviral therapy adherence among adolescents in low- and middle-income countries: A systematic review of the literature. *PloS one, 13*(1), e0189770. doi: 10.1371/journal.pone.0189770

Rodrigues, R., Shet, A., Antony, J., Sidney, K., Arumugam, K., Krishnamurthy, S., . . . DeCosta, A. (2012). Supporting adherence to antiretroviral therapy with mobile phone reminders: results from a cohort in South India. *PloS one, 7*(8), e40723. doi: 10.1371/journal.pone.0040723

Rosalind M. Parkes-Ratanshi, Maria S. Nabaggala, Agnes N. Bwanika, Mohammed Lamorde, Rachel King, Noela Owarwo, . . . Kiragga, A. (2019). *CALL FOR LIFE UGANDA TM: An RCT Using Interactive Voice Response for PLHIV on ART. Abstract 1037.* . Paper presented at the Conference on Retroviruses and Opportunistic Infections,, Seattle.

MAKERERE UNIVERSITY
SCHOOL OF MEDICINE
APPROVED
VALID UNTIL
10 FEB 2021
RESEARCH & ETHICS COMMITTEE
P.O.BOX 7072, KAMPALA

Roy, D., Tripathy, S., Kar, S. K., Sharma, N., Verma, S. K., & Kaushal, V. (2020). Study of knowledge, attitude, anxiety & perceived mental healthcare need in Indian population during COVID-19 pandemic. *Asian Journal of Psychiatry, 51*, 102083. doi: https://doi.org/10.1016/j.ajp.2020.102083

Sankaranarayanan, J., & Sallach, R. E. (2014). Rural patients' access to mobile phones and willingness to receive mobile phone-based pharmacy and other health technology services: a pilot study. *Telemed J E Health, 20*(2), 182-185. doi: 10.1089/tmj.2013.0150

Schnall, R., Bakken, S., Brown Iii, W., Carballo-Dieguez, A., & Iribarren, S. (2016). Usabilty Evaluation of a Prototype Mobile App for Health Management for Persons Living with HIV. *Studies in health technology and informatics, 225*, 481-485.

Schnall, R., Bakken, S., Rojas, M., Travers, J., & Carballo-Dieguez, A. (2015). mHealth Technology as a Persuasive Tool for Treatment, Care and Management of Persons Living with HIV. *AIDS Behav, 19 Suppl 2*(0 2), 81-89. doi: 10.1007/s10461-014-0984-8

Schnall, R., Rojas, M., Bakken, S., Brown, W., Carballo-Dieguez, A., Carry, M., . . . Travers, J. (2016). A user-centered model for designing consumer mobile health (mHealth) applications (apps). *J Biomed Inform, 60*, 243-251. doi: 10.1016/j.jbi.2016.02.002

Schroder, K. E., Johnson, C. J., & Wiebe, J. S. (2007). Interactive Voice Response Technology applied to sexual behavior self-reports: a comparison of three methods. *AIDS Behav, 11*(2), 313-323. doi: 10.1007/s10461-006-9145-z

Sheehan, B., Lee, Y., Rodriguez, M., Tiase, V., & Schnall, R. (2012). A comparison of usability factors of four mobile devices for accessing healthcare information by adolescents. *Applied clinical informatics, 3*(04), 356-366.

Siedner, M. J., Haberer, J. E., Bwana, M. B., Ware, N. C., & Bangsberg, D. R. (2012). High acceptability for cell phone text messages to improve communication of laboratory results with HIV-infected patients in rural Uganda: a cross-sectional survey study. *BMC Med Inform Decis Mak, 12*(1), 56. doi: 10.1186/1472-6947-12-56

Smillie, K., Van Borek, N., van der Kop, M. L., Lukhwaro, A., Li, N., Karanja, S., . . . Lester, R. T. (2014). Mobile health for early retention in HIV care: a qualitative study in Kenya (WelTel Retain). *African journal of AIDS research : AJAR, 13*(4), 331-338. doi: 10.2989/16085906.2014.961939

Sparkes, J., Valaitis, R., & McKibbon, A. (2012). A usability study of patients setting up a cardiac event loop recorder and BlackBerry gateway for remote monitoring at home. *Telemedicine and e-Health, 18*(6), 484-490.

Steve Kanters, J. J. H. P., Keith Chan, Maria Eugenia Socias, Nathan Ford, Jamie I Forrest, Kristian Thorlund, Jean B Nachega, Edward J Mills. (2016). Interventions to improve adherence. *www.theLancet.com/HIV*. doi: 10.1016/S2352-3018(16)30206-5

Swahn , M. H., Braunstein, S., & Kasirye, R. (2014). Demographic and Psychosocial Characteristics of Mobile Phone Ownership and Usage among Youth Living in the Slums of Kampala, Uganda. *Western Journal of Emergency Medicine*. doi: 10.5811/westjem.2014.4.20879

Swindells, S., Mohr, J., Justis, J. C., Berman, S., Squier, C., Wagener, M. M., & Singh, N. (1999). Quality of life in patients with human immunodeficiency virus infection: impact of social support, coping style and hopelessness. *Int J STD AIDS, 10*(6), 383-391.

Tomlinson, M., & Rotheram-Borus, e. a. (2013). Scaling up mHealth: where is the evidence? *PLoS Med, 10*(2), e1001382. doi: 10.1371/journal.pmed.1001382

Tuller, D. M., Bangsberg, D. R., Senkungu, J., Ware, N. C., Emenyonu, N., & Weiser, S. D. (2010). Transportation costs impede sustained adherence and access to HAART in a clinic population in southwestern Uganda: a qualitative study. *AIDS Behav, 14*(4), 778-784. doi: 10.1007/s10461-009-9533-2

UBOS. (2017). UGANDA BUREAU OF STATISTICS : 2017 Statistical_Abstract: Communication Statistics.

UBOs. (2018). Statistical Report

UNAIDS. (2014). 90-90-90 An ambitious treatment target to help end the AIDS epidemic.

MAKERERE UNIVERSITY
SCHOOL OF MEDICINE
APPROVED
VALID UNTIL
10 FEB 2021
RESEARCH & ETHICS COMMITTEE
P.O.BOX 7072, KAMPALA

UNAIDS. (2017). STATE OF THE AIDS EPIDEMIC-UNAIDS DATA 2017.

UNAIDS. (2018a). UNAIDS Data FactSheet.

UNAIDS. (2018b). Youth and HIV.

UNAIDS. (2019a, 2019). Ending the AIDS epidemic among children, adolescents and young women. Retrieved 27 Apr, 2018, from https://www.unaids.org/en/resources/presscentre/featurestories/2018/april/ending-aids-epidemic-among-children-adolescents-young-women

UNAIDS. (2019b). Global AIDS Update 2019.

UPHIA. (2017a). UGANDA POPULATION-BASED HIV IMPACT ASSESSMENT UPHIA 2016–2017.

UPHIA. (2017b). UGANDA POPULATION-BASEDHIV IMPACT ASSESSMENT.

van der Kop ML, O. D., Patel A, et al. (2013). The effect of weekly short message service communication on patient retention in care in the first year after HIV diagnosis: study protocol for a randomised controlled trial (WelTel Retain). *BMJ*. doi: 10.1136/bmjopen-2013003155

10.1136/bmjopen-2013-003155

Vreeman, R. C., Nyandiko, W. M., Liu, H., Tu, W., Scanlon, M. L., Slaven, J. E., . . . Inui, T. S. (2014). Measuring adherence to antiretroviral therapy in children and adolescents in western Kenya. *Journal of the International AIDS Society, 17*(1), 19227.

Ware, N. C., Pisarski, E. E., Tam, M., Wyatt, M. A., Atukunda, E., Musiimenta, A., . . . Haberer, J. E. (2016). The Meanings in the messages: how SMS reminders and real-time adherence monitoring improve antiretroviral therapy adherence in rural Uganda. *AIDS (London, England), 30*(8), 1287-1294. doi: 10.1097/QAD.0000000000001035

WHO. (2006). *Electronic Health Records Manual For Developing Countries*.

WHO. (2011). mHealth: New horizons for health through mobile technologies *Global Observatory for eHealth series* (Vol. 3).

WHO. (2013). Consolidated guidelines on the use of antiretroviral drugs for treating and preventing HIV infection

WHO. (2016). Guidelines on HIV self-testing and partner notification; Suppliment to consolidated guidelines on HIV testing services. .

Amith, M.; Loubser, PG.; Chapman, J.; Zoker, KC.; Rabelo Ferreira, FE. Optimization of an EHR mobile application using the UFuRT conceptual framework.. AMIA Annual Symposium proceedings / AMIA Symposium AMIA Symposium; 2012; p. 209-17.

Luxton DD, Mishkind MC, Crumpton RM, Ayers TD, Mysliwiec V. Usability and feasibility of smartphone video capabilities for telehealth care in the U.S. military. Telemedicine journal and ehealth : the official journal of the American Telemedicine Association. 2012; 18(6):409–12. [PubMed: 22650351]

Burnay E, Cruz-Correia R, Jacinto T, Sousa AS, Fonseca J. Challenges of a mobile application for asthma and allergic rhinitis patient enablement-interface and synchronization. Telemedicine journal and e-health: the official journal of the American Telemedicine Association. 2013; 19(1): 13–8. [PubMed: 23215639]

Sheehan B, Lee YJ, Rodriguez M, Tiase V, Schnall R. A Comparison of Usability Factors of Four Mobile Devices for Accessing Healthcare Information by Adolescents. Applied clinical informatics. 2012; 3(4):356–66. [PubMed: 23227134]

Sparkes J, Valaitis R, McKibbon A. A usability study of patients setting up a cardiac event loop recorder and BlackBerry gateway for remote monitoring at home. Telemedicine journal and ehealth : the official journal of the American Telemedicine Association. 2012; 18(6):484–90. [PubMed: 22676379

MAKERERE UNIVERSITY
SCHOOL OF MEDICINE
APPROVED
VALID UNTIL
10 FEB 2021
RESEARCH & ETHICS COMMITTEE
P.O.BOX 7072, KAMPALA

Kim J et al. 2015) Journal of the International AIDS Society 2015, 18:20311 http://www.jiasociety.org/index.php/jias/article/ view/20311| http://dx.doi.org/10.7448/IAS.18.1.20311)

Swahn et al, 2014. Mobile Phone Ownership and Usage among Youth. Western Journal of Emergency Medicine, Volume XV, NO. 5 : August 2014

H. Anglada-Martinez, G. Riu-Viladoms, M. Martin-Conde, et al. 2015. Does mHealth increase adherence to medication? Results of a systematic review. Int J Clin Pract, January 2015, 69, 1, 9–32. doi: 10.1111/ijcp.12582

MAKERERE UNIVERSITY
SCHOOL OF MEDICINE
APPROVED
VALID UNTIL
10 FEB 2021
RESEARCH & ETHICS COMMITTEE
P.O.BOX 7072, KAMPALA

5. List of possible manuscripts

I. Acceptability of mHealth Call for life tool among Youths initiating ART in Kiryandongo

II. Baseline qualitative paper on Self-reported stigma, current barriers and enablers of ART among Youth initiating ART

III. Effect of mHealth on ART adherence, retention in care and viral load suppression among YLHIV

IV. Cost of mHealth on retention in care of youths in comparison to standard of Care

6. Appendices

i. Budget:

MAKERERE UNIVERSITY
SCHOOL OF MEDICINE
APPROVED
VALID UNTIL
10 FEB 2021
RESEARCH & ETHICS COMMITTEE
P.O.BOX 7072, KAMPALA

Study Personnel			%	1.07		34.77%	
		TYPE	EFFORT	Base	Salary		Total Amount
	ROLE ON	APPT.	ON	Salary	Requested	Fringe Benefit	
NAME	PROJECT	(months)	PROJECT			0	
Agnes Bwanika Naggirinya	PI	12	0%	$ 1,965	$ -	-	-
NURSE COUNSELLOR	Research Assistant	18	100%	$ 571	$ 10,285.71	3,576	13,862
NURSE COUNSELLOR	Research Assistant	12	100%	$ 571	$ 6,857.14	2,384	9,241
	Sub total						$ 23,103
TRAVEL		Number of Uni	Rate	Frequency			
Local travel							-
Qualitative surveys		4	$25	1			100
Patient reimbursement for study visit (Estimate of $7 per patient)	Estimated cost per patient	200	$7	4			5,600
Local transport aggregated	Estimates for Kiryandongo	2	$200	12			4,800
Accomodation	Estimated cost	4	$15	10			600
	Sub total						$ 11,100
SUPPLIES&CONSUMABLES							
Office stationery(filing cabins, files)	Assorted	1	$500	1			500
Modems		2	$50	1			100
Data for Modems	subscription/ month	2	$10	12			240
transcription of qualitative work		1	$150	2			300
	Sub total						$ 1,140
							-
SUBAWARDS & SUBCONTRACTS							-
Aspect-voxeo							-
SSL rate		1	$0	12			-
Cost for outbound calls per month	Monthly Minimum advance	12.00	0	$3,000			-
Toll free forwarding.com							-
Registration	@0.61 per pt	1	0.61	100			-
Symptom reporting/ tips	@0.61 per pt per call	12	0.61	100			-
Red Cap set-up	4 questionnaires @100	1	$100	4			400
Red Cap IT-Support		1	$500	1			500
	Sub total						900
EQUIPMENT (Itemize)							-
Cell phones		200	$10	1			2,000
2 laptop		2	$1,200		$ -		2,400
	Sub total						$ 4,400
OTHER COSTS							-
Sensitization and Protocol training		2	$100	1			200
Stakeholder / DSMB meetings		3	$43	10			1,286
IDI Support Costs			$300	12			-
Printing	Average usage per month	1	$20	12			240
2 staff airtime	per month	1	$15	12			180
Phone calls-		1	$20	12			240
	Sub total						$ 2,146
Laboratory tests							-
Vacutainers, gloves and swabs, vacutainer needle & holder		1	$55	4			220
Storage of samples		200	$4	1			800
Resistance testing (5% predicted VL detectable)		10	$150	1			1,500
	Sub total						$ 2,520
IRB fees		1	$1,500	0	$ -	$ -	$ -
Sub Total					$ -		-
TOTAL COSTS							45,309
IDI Overhead 11.04%%							5,002
TOTAL COSTS FOR BUDGET PERIOD							60,311

MAKERERE UNIVERSITY
SCHOOL OF MEDICINE
APPROVED
VALID UNTIL
10 FEB 2021
RESEARCH & ETHICS COMMITTEE
P.O.BOX 7072, KAMPALA

**PhD Study Time Lines**	Jan – June 2018	July-sept 2018	Oct-Dec 2018	Jan-March 2019	April-June 2019	July-Sept 2019	Oct-Dec 2019	Jan-March 2020	April-June 2020	July-Sept 2020	Oct-Dec 2020	Jan-March 2021	April-June 2021
Concept Development presentation to Dept. of Medicine													
Concept Presentation to HDREC													
Concept responses to comments and Approval, DRGT partial admission													
Clearance from Kiryandongo Hospital Kiryandongo DHO, Systematic Review													
Proposal Development IRB applications approvals													
IDI-SRC proposal submission and clearance													
Recruit research assistants													
SOM-HDREC- proposal presentation													
SOM-HDREC final approval													
Submission of Full proposal to School of Graduate Studies													
UNCST application and clearance													
Protocol training, set of systems													
Recruitment Collection of baseline data – questionnaires qualitative													
Follow up of YLWH(Qualitative) data collection for Quantitative arm													
Interim data analysis- baseline qualitative													
Final data analysis primary outcome													
Continuing IRB Review													
Analysis of qualitative data													
Manuscript write-up first paper													
Manuscript write-up second paper													
Manuscript write-up third paper													

ii. Timeframe/work plan

MAKERERE UNIVERSITY
SCHOOL OF MEDICINE
APPROVED
VALID UNTIL
10 FEB 2021
RESEARCH & ETHICS COMMITTEE
P.O.BOX 7072, KAMPALA

MAKERERE UNIVER
SCHOOL OF MEDICINE
APPROVED
VALID UNTIL
10 FEB 2021
RESEARCH & ETHICS COMMITTEE
P.O.BOX 7072, KAMPALA

iii.

Informed Consent Document

Participant Information

Study Title: Acceptability, effect and cost of mHealth on ART Adherence and knowledge of COVID-19 Among Youths Initiating ART or on ART since Jun 2019 at Kiryandongo: A Mixed Methods Sequential Study

Invitation:

You are being invited to take part in this research project. Before you decide to do so, it is important you understand why the research is being conducted and what it will involve. Please take time to read the following information carefully. Ask us if there is anything that you are not clear of or if you would like more information.

Who we are:

We are researchers from Makerere University, working on the Youth Call for life Study, which is focusing on youth. The Youth Call for life study is being sponsored by Ugandan Academy for Health Innovations Impact at Infectious Diseases Institute, which is under college of Health Sciences, Makerere University.

What is the purpose of the study?

Our main reason for conducting this study is to gather evidence that will enable us improve the youth' pill adherence, clinic attendance, retention in clinical care as a means to improve on viral load suppression. This study will help us to better understand the things that make it difficult for youths to: disclose their status; attend clinic; take their ARVs and retain in care. A total of two hundred and six youths will be recruited for this study. We shall aslo assess knowledge of COVID-19 and to demystify poor understanding of the disease which can result in delayed identification and to rapid spread of infections if you don't seek help early.

What will happen if I decide to take part in this study

If you agree to take part in the study, we will ask you to share your views on use of mobile health to help youths, clinic attendance and retention in care. You will be seen at the beginning of the study and every 6 months up to 1 year. Some blood around 8 mls will be taken off at each of these visits. You might be requested to participate in group discussions to share experiences, views and discuss about certain topics.

If you agree to take part in this research study, you will be asked to read, sign and date the consent form attached to this information sheet. This will happen after the study has been verbally explained to you by your doctor and/or his designee, and all your questions have been answered. A copy of the signed, completed consent form would be given to you for your information and

records. If you permit, your doctor will inform your primary care doctor of your participation in this trial.

You may already be taking ART medication at the time of enrollment, or will start ART at the same time as the study. The choice of the medication and the decision to start or change medication is not related to this study. The HIV medication is not being made available as a part of this study. HIV medication is provided free of charge as part of your routine care in the hospital HIV clinic.

There are two groups of patients in this study. One group will be registered on the Call for Life system immediately and the second group will not be registered on the system. When you sign to join this study, you or your clinician will not be able to determine what group you join. You will be asked to pick an envelope containing a paper that will determine what group you will join.

After you are consented for the study, the envelope will dictate whether you register on Call for Life· you will receive daily interactive voice response (IVR) mobile phone support (calls from Call for Life Uganda). This support will include adherence reminder calls and health tips (HIV general topic, ART adherence and sexuality) plus any health education regarding HIV requested during the focus group discussions. If you request or the study team feels it is important you may receive the messages more than once per week, but this will be discussed and agreed upon with you. If you are unwell or have any symptoms the Call for Life Uganda system can be called and report your symptoms, which will be responded to within 24hrs on a toll free number that will be provided to those on Call for life study arm.

If you decide to be part of this study, you (your child) will come to the clinic monthly to pick up ART and other medications as per standard of care. These visits are routine and not related to this study. The study visits will be every 6 months for a period of 1 year and will coincide with blood draws.

Do I have to take part in this study?

Your participation is voluntary. You may withdraw from the study at any time and without giving a reason. Your decision will not affect your clinical care at the hospital. You may choose to discuss some topics if you decide to participate in group discussions.

Do I need to decide now?

No, you don't need to decide about participating in the study now. If you need more time to consider your decision, we will give you up to five days to do so. You will be given this information leaflet to take with you to help understand better. You may call the researchers on the contact details at the bottom to seek more clarification or discuss any concerns you may have about the study.

How will the information I tell, be kept confidential?

MAKERERE UNIVERSITY
SCHOOL OF MEDICINE
APPROVED
VALID UNTIL
10 FEB 2021
RESEARCH & ETHICS COMMITTEE
P.O.BOX 7072, KAMPALA

The study staff will be the only ones to know your personal information. Your personal records will be kept in a secure and confidential location. You will be given a code that will be used instead of your name.

The results of the tests and your comments may be used in a scientific publication, but your names will not appear in any publication of the study results. The study staff will use a computer that will include all the study data details. All the computers containing the study data base will be password protected and the study data base will not be accessible by personnel not associated with the study. The results of the study will be kept strictly confidential and used only for research purposes. No information concerning the study or the data generated from the study will be released to any unauthorized third party without prior written approval of the sponsor and the subject. Your identity will be kept confidential in so far as the study allows. All information will be kept on coded forms. Your names or other information that could be used to identify you will not appear on any notes or any interview notes if you participate in the group discussions.

What are the risks and benefits in taking part?

There were no major risks identified from this study. The risk may include temporarily not being able to access Call for Life Uganda sservices due to technical breakdown. It is also possible that some participants may not find Call for Life Uganda services easy to access or to understand or that the services do not help in adhering to their medication or accessing advise to manage any medical symptoms or ART related side effects. It is possible that some participants may get some violence from spouses related to frequent phone calls, this will be assessed frequently and counselling will be accorded for those affected. You must remember to contact your clinic directly if the mobile based Call for Life Uganda service is not providing you the help you need to manage your condition, getting a phone may lead to community associating it to participating in this study and may cause stigma. Efforts to get various models have been made to reduce this risk.

In order to make sure that your HIV status is not disclosed to anyone, when you receive your phone call you will hear some music until you enter a code that is for you only, and then the system will start to talk to you. IF someone doesn't know your code they cannot access the information. We shall use only voice calls and we do not intend to give text messages in this study, however if you agree to text messages, then there is a risk of another person reading your information if they have access to your phone. If you would like text messages, we would advise that you protect the confidentiality of your phone by adding a passcode as security.

In this study you may receive mobile phone call support that can be beneficial for your health. A study in Kenya showed that weekly text messages helped people to adhere to their, clinic visits, medication and improve on virological outcomes. You might also get health tips that may help you to manage and improve your knowledge on HIV and other conditions, and may be interesting to you. You also may get some personal satisfaction from being part of research on HIV. From the blood sample that we will collect, we may be able to get additional information about your response to ART and use it to benefit your health. Also, you or others may benefit in the future from what is learned in this study.

MAKERERE UNIVERSITY
SCHOOL OF MEDICINE
APPROVED
VALID UNTIL
10 FEB 2021
RESEARCH & ETHICS COMMITTEE
P.O.BOX 7072, KAMPALA

What if something goes wrong?

If you have any complaints about the study, you can contact any member of the research team (details below). If you feel your complaint has not been handled to your satisfaction, you can contact any of the Chair Ethics Committee or Kiryandongo district health Office that have cleared the study.

What are the costs incurred while taking part in this study?

There are no known direct costs to you for taking part in this study. We shall **NOT** reimburse 20,000/= UGX as travel refund and time spent with us per scheduled visit since it will coincide with the routine standard clinic visits, and might interfere with one of the study's objective; but those who will attend the group discussion will receive this reimbursement. All enrolled participants will receive a basic mobile phone and a water bottle.

What will happen to the results of the study?

Results of the study will be disseminated to the study participants, hospital staff and will be published too, as a requirement for doctoral studies. You will not be identified in any report or publication. If you wish to be given a copy of any reports resulting from the study, please ask us to put you on the circulation list.

Who has ethically reviewed this study?

This study has been ethically cleared and approved by the School of Medicines Higher Degrees Research Ethics Committee Makerere College of Health Sciences, and Research clearance obtained from Uganda National Council of Science Technology.

What will I do if I have questions about the study?

If you have any questions or what you would like to understand about this study, first talk to the study staff, ask Dr. Agnes Bwanika N (Infectious Diseases Institute, P.O. Box 22418, Kampala Uganda) or call telephone number +256-312-307000/ +256 752 521 570.

In case you want to ask something about your rights as a study participant contact the Chairman of the Higher Degrees Ethics Committee of Makerere University College of Health Sciences and Department of Medicine-Mulago Hospital Prof. Ponsiano Ocama, on telephone number +256772421190.

MAKERERE UNIVERSITY
SCHOOL OF MEDICINE
APPROVED
VALID UNTIL
10 FEB 2021
RESEARCH & ETHICS COMMITTEE
P.O.BOX 7072, KAMPALA

Study procedures	Baseline<br><br>Month 00 + 2 weeks	Month 6± 2 weeks	Month 12± 2 weeks
Screening/ Barriers, enablers of adherence and acceptability for all potential participants	√		
Informed Consent	√		
COVID-19 Knowledge Assessment	√		
Stigma Scores	√		√
Demographic and socio-economic assessment, Knowledge questionnaire, Sexual behavior questionnaire	√	√	√
Randomization	√		
Laboratory			
Viral load ±CD4*	√*	√	√
Resistance testing(If funds allow)			√**
Plasma storage **	√	√	√
Qualitative assessment	√		√

* For those who are initiating or are ART naïve at study entry

** If funds allow

MAKERERE UNIVERSITY
SCHOOL OF MEDICINE
APPROVED
VALID UNTIL
10 FEB 2021
RESEARCH & ETHICS COMMITTEE
P.O.BOX 7072, KAMPALA

Consent Form

By signing this form, I agree that (tick the appropriate box):

I have received the participant information; the study has been explained to me in the language that I understand. All the questions I had about the study have been answered. I understand what will happen during the study and what is expected of me. **Yes** ☐ **No** ☐

I have been informed that is my right to refuse to take part in the study, and if I choose not to, I don't have to give a reason. **Yes** ☐ **No** ☐

I'm happy to take part in this study. **Yes** ☐ **No** ☐

Statement of Consent

I have received enough explanations concerning the benefits and details concerning participation in this study. I have been informed that I have rights not to participate in this study anytime I choose, or not to respond to any uncomfortable questions. I will be given a copy of this consent form to keep if I want. I have agreed to participate in the study.

Participant Name | Participant signature or thumb print | Date dd-mon-year

Witness' name signature | Date dd-mon-year

Name or signature of person obtaining consent | Date dd-mon-year

MAKERERE UNIVERSITY
SCHOOL OF MEDICINE
APPROVED
VALID UNTIL
10 FEB 2021
RESEARCH & ETHICS COMMITTEE

MAKERERE UNIVERSITY
SCHOOL OF MEDICINE
APPROVED
VALID UNTIL
10 FEB 2021
RESEARCH & ETHICS COMMITTEE
P.O.BOX 7072, KAMPALA

iv. Assent Document

Participant Information:

Study Title: Acceptability, effect and cost of mHealth on ART Adherence and knowledge of COVID-19 Among Youths Initiating ART or on ART since Jun 2019 at Kiryandongo: A Mixed Methods Sequential Study

Invitation:

Your child is being invited to take part in this research project. Before you decide to do so, it is important you understand why the research is being conducted and what it will involve. Please take time to read the following information carefully. Ask us if there is anything that you are not clear of or if you would like more information.

Who we are:

We are researchers from Makerere University, working on the Youth Call for life Study, which is focusing on youth. The Youth Call for life study is being sponsored by the Academy for Health Innovations at Infectious Diseases Institute, which is under the College of Health Sciences, Makerere University.

What is the purpose of the study?

Our main reason for conducting this study is to gather evidence that will enable us improve the youth' pill adherence, clinic attendance, retention in clinical care as a means to improve on viral load suppression. This study will help us to better understand the things that make it difficult for youths to: disclose their status; attend clinic; take their ARVs and retain in care. A total of two hundred and six youths will be recruited for this study. We shall aslo assess knowledge of COVID-19 and to demystify poor understanding of the disease which can result in delayed identification and to rapid spread of infections if you don't seek help early.

What will happen if when you decide to take part in this study:

If you agree on behalf of your child to take part in the study, we will ask your child to share views on use of mobile health to help youths, clinic attendance and retention in care. The child will be seen at the beginning of the study and every 6 months up to 1 year. Some blood around 8 mls will be taken off at each of these visits. Your child might be requested to participate in group discussions to share experiences, views and discuss about certain topics.

If you agree on behalf of your child to take part in this research study, you will be asked to read, sign and date this Assent form attached to this information sheet. This will happen after the study has been verbally explained to you by your doctor and/or the designee, and all your questions have been answered. A copy of the signed, completed Assent form would be given to you for your

information and records. If you permit, the study clinician will inform your child's primary care doctor of the participation in this trial.

Your child may already be taking ART medication at the time of enrollment, or will start ART at the same time as the study. The choice of the medication and the decision to start or change medication is not related to this study. The HIV medication is not being made available as a part of this study. HIV medication is provided free of charge as part of routine care in the hospital HIV clinic.

There are two groups of patients in this study. One group will be registered on the Call for Life system immediately and the second group will not be registered on the system. When you sign on behalf of your child to join this study, you or your clinician will not be able to determine what group your child will join. Your child will be asked to pick an envelope containing a paper that will determine what group to will join.

After you are consented on behalf of your child for the study, the envelope will dictate whether your child register on Call for Life, the child will receive daily interactive voice response (IVR) mobile phone support (calls from Call for Life Uganda). This support will include adherence reminder calls and health tips (HIV general topic, ART adherence and sexuality) plus any health education regarding HIV requested during the focus group discussions. If you request or the study team feels it is important your child may receive the messages more than once per week, but this will be discussed and agreed upon with you. If your child is unwell or have any symptoms the Call for Life Uganda system can be called and report symptoms, which will be responded to within 24hrs on a toll free number that will be provided to only those on the Call for life study arm.

If you decide on behalf of your child to be part of this study, your child will come to the clinic monthly to pick up ART and other medications as per standard of care. These visits are routine and not related to this study. The study visits will be every 6 months for a period of 1 year and will coincide with blood draws.

Do I have to take part in this study?

Your child's participation is voluntary. The child may withdraw from the study at any time and without giving a reason. Your decision will not affect the child's clinical care at the hospital. Your child may choose to discuss some topics if decides to participate in group discussions.

Do I need to decide now?

No, you don't need to decide about participating in the study now. If you need more time to consider your decision, we will give you up to five days to do so. You will be given this information leaflet to take with you to help understand better. You may call the researchers on the contact details at the bottom to seek more clarification or discuss any concerns you may have about the study.

How will the information I tell, be kept confidential?

MAKERERE UNIVERSITY
SCHOOL OF MEDICINE
APPROVED
VALID UNTIL
10 FEB 2021
RESEARCH & ETHICS COMMITTEE
P.O.BOX 7072, KAMPALA

The study staff will be the only ones to know the child's personal information. All personal records will be kept in a secure and confidential location. Your child will be given a code that will be used instead of the name.

The results of the tests and your child's comments may be used in a scientific publication, but your child's names will not appear in any publication of the study results. The study staff will use a computer that will include all the study data details. All the computers containing the study data base will be password protected and the study data base will not be accessible by personnel not associated with the study. The results of the study will be kept strictly confidential and used only for research purposes. No information concerning the study or the data generated from the study will be released to any unauthorized third party without prior written approval of the sponsor and the subject. Your child's identity will be kept confidential in so far as the study allows. All information will be kept on coded forms. Your child's names or other information that could be used to identify the child will not appear on any notes or any interview notes if your child participate in the group discussions.

What are the risks and benefits in taking part?

There are no major risks identified in this study. The risk may include temporarily not being able to access Call for Life Uganda services due to technical breakdown. It is also possible that some participants may not find Call for Life Uganda services easy to access or to understand or that the services do not help in adhering to their medication or accessing advice to manage any medical symptoms or ART related side effects. It is possible that some participants may get some violence from spouses related to frequent phone calls, this will be assessed frequently and counselling will be accorded for those affected. You must remember to contact your clinic directly if the mobile based Call for Life Uganda service is not providing you the help you need to manage your condition, getting a phone may lead to community associating it to participating in this study and may cause stigma. Efforts to get various models have been made to reduce this risk.

In order to make sure that your HIV status is not disclosed to anyone, when you receive your phone call you will hear some music until you enter a code that is for you only, and then the system will start to talk to you. IF someone doesn't know your code they cannot access the information. We shall use only voice calls and we do not intend to give text messages in this study, however if you agree to text messages, then there is a risk of another person reading your information if they have access to your phone. If you would like text messages, we would advise that you protect the confidentiality of your phone by adding a passcode as security.

In this study you may receive mobile phone call support that can be beneficial for your health. A study in Kenya showed that weekly text messages helped people to adhere to their, clinic visits, medication and improve on virological outcomes. You might also get health tips that may help you to manage and improve your knowledge on HIV and other conditions, and may be interesting to you. You also may get some personal satisfaction from being part of research on HIV. From the blood sample that we will collect, we may be able to get additional information about your

MAKERERE UNIVERSITY
SCHOOL OF MEDICINE
APPROVED
VALID UNTIL
10 FEB 2021
RESEARCH & ETHICS COMMITTEE
P.O.BOX 7072, KAMPALA

response to ART and use it to benefit your health. Also, you or others may benefit in the future from what is learned in this study.

What if something goes wrong?

If you have any complaints about the study, you can contact any member of the research team (details below). If you feel your complaint has not been handled to your satisfaction, you can contact any of the Chair Ethics Committee or Kiryandongo district health Office that have cleared the study. All participants will receive a basic mobile phone and a water bottle.

What are the costs incurred while taking part in this study?

There are no known direct costs to you for taking part in this study. We shall NOT reimburse 20,000/= UGX as travel refund and time spent with us per scheduled visit since it will coincide with the routine standard clinic visits, and might interfere with one of the study's objective but those who will attend the group discussion will receive this reimbursement.

What will happen to the results of the study?

Results of the study will be disseminated to the study participants, hospital staff and will be published too, as a requirement for doctoral studies. You will not be identified in any report or publication. If you wish to be given a copy of any reports resulting from the study, please ask us to put you on the circulation list.

Who has ethically reviewed this study?

This study has been ethically cleared and approved by the School of Medicines Higher Degrees Research Ethics Committee Makerere College of Health Sciences, and Research clearance obtained from Uganda National Council of Science Technology.

What will I do if I have questions about the study?

If you have any questions or what you would like to understand about this study, first talk to the study staff, ask Dr. Agnes Bwanika N (Infectious Diseases Institute, P.O. Box 22418, Kampala Uganda) or call telephone number +256-312-307000/ +256 752 521 570.

In case you want to ask something about your rights as a study participant contact the Chairman of the Higher Degrees Ethics Committee of Makerere University College of Health Sciences and Department of Medicine-Mulago Hospital Prof. Ponsiano Ocama, on telephone number +256772421190.

MAKERERE UNIVERSITY
SCHOOL OF MEDICINE
APPROVED
VALID UNTIL
10 FEB 2021
RESEARCH & ETHICS COMMITTEE
P.O.BOX 7072, KAMPALA

Study procedures	Baseline<br><br>Month 00 + 2 weeks	Month 6± 2 weeks	Month 12± 2 weeks
Screening/ Barriers, enablers of adherence and acceptability for all potential participants	√		√
Informed Consent	√		
COVID-19 Knowledge assessment	√		
Stigma Scores	√		√
Demographic and socio-economic assessment, Knowledge questionnaire, Sexual behavior questionnaire	√	√	√
Randomization	√		
Laboratory			
Viral load ±CD4	√*	√	√
Resistance testing(If funds allow)			√**
Plasma storage **	√	√	√
Qualitative assessment	√		√

* For those who are initiating or are ART naïve at study entry

** If funds allow

MAKERERE UNIVERSITY
SCHOOL OF MEDICINE
APPROVED
VALID UNTIL
10 FEB 2021
RESEARCH & ETHICS COMMITTEE
P.O.BOX 7072, KAMPALA

Assent Form

By signing this form, I agree on behalf of my child that (tick the appropriate box)

I have received the participant information; the study has been explained to me in the language that I understand. All the questions I had about the study have been answered. I understand what will happen to my child during the study and what is expected of me. Yes ☐ No ☐

I have been informed that is my right to refuse my child to take part in the study, and if I choose not to, I don't have to give a reason. Yes ☐ No ☐

I'm happy for my child to take part in this study. Yes ☐ No ☐

Statement of Consent

I have received enough explanations concerning the benefits and details concerning participation in this study. I have been informed that I have rights not to participate in this study anytime I choose, or not to respond to any uncomfortable questions. I will be given a copy of this consent form to keep if I want. I have agreed for my child to participate in the study.

Participant Name	Guardian's signature or thumb print	Date (dd-mon-year)

Guardian's names	Date (dd-mon-year)

Name or signature of person obtaining consent	Date (dd-mon-year)

MAKERERE UNIVERSITY
SCHOOL OF MEDICINE
APPROVED
VALID UNTIL
10 FEB 2021
RESEARCH & ETHICS COMMITTEE
P.O.BOX 7072, KAMPALA

v. CFL FGD Guide Youth Study

Call for Life Youth Study
Focus Group Discussion Guide

Introduction:

Hi, my name is _____________ and I want to thank you for joining us today. I am helping to coordinate this study on CFLU system here in Kiryandongo. We are conducting this research to identify barriers and enablers of ART adherence among youths in Kiryandongo District and get views about mHealth interventions; what you like, what you don't like and reasons for liking and not liking the system. I would like to say that there are no right or wrong answers in our discussion. We will simply be discussing your views, opinions and experiences on a range of topics, so please feel comfortable to say what you honestly feel. I would like to tape record the whole session. Please do not be concerned about this, all measures will be taken to maintain confidentiality of the interviews and discussions. Information you tell us will ONLY be used for this research project. As we are tape recording the interview, we ask that you refrain from using names or identifying information of yourself or your partners. If at any time during the interview you feel uncomfortable you can ask for a break, refuse to answer any question, and are always free to leave. Do you have any questions before we start?

Create rapport:

How have you been?

mHealth acceptability among youth

Now that you have received information about mHealth /CFLU, tell us what you know about mHealth/CFLU

1. As a youth, would you accept or refuse to use mHealth CFLU tool?
2. Tell us why you would accept or refuse to use mHealth interventions/ CFLU
3. Can you name the specific elements you would like to help in ART adherence? (Probe on pill reminders, health tips, symptom reporting, use of secret pin)
4. How can the system be used? (Probe on timing - during what hours; frequency)
5. What do you ***not*** like about the system? Please describe.
    a. Probe on: reminders, health tips, timing, voice, etc
    b. Probe on why

Barriers, enablers of ART adherence among youths (To phrase questions 4&5 based on ART experience)

MAKERERE UNIVERSITY
SCHOOL OF MEDICINE
APPROVED
VALID UNTIL
10 FEB 2021
RESEARCH & ETHICS COMMITTEE
P.O.BOX 7072, KAMPALA

1. Tell me everything you know about ART adherence.
2. Who explained to you how to be adherent?
3. Can you think of reasons why youths can't take their medication as they are supposed to do?
4. What is likely to facilitate/ hinder ART adherence/ clinic attendance (ART naives)
5. What facilitated /hindered your adherence to ART/ clinic attendance (ART experienced)

Probe on:

- i. Characteristic of individual his/her environment
- ii. Treatment regimen
- iii. Healthcare providers
- iv. Client-provider relationship
- v. Health Care setting
- vi. Psychosocial issues
- vii. Education level
- viii. knowledge of HIV ART

MAKERERE UNIVERSITY
SCHOOL OF MEDICINE
APPROVED
VALID UNTIL
10 FEB 2021
RESEARCH & ETHICS COMMITTEE
P.O.BOX 7072, KAMPALA

Question Guide below 1- 10 to be used only at study end line

1. If you had to rate your comfort level using the system on a scale from 1 to 5 (5 being most comfortable), how would you rate the experience in general?
2. Can you talk about the reasons you gave the rating? what makes you feel comfortable using and not using the system?
3. How do you think that this system can help patients? Probe on specifics.
4. (if relevant) Can you describe how this system may have helped you take your drugs?
5. Let's talk about the health tips.
   1. How often do you follow the advice of the health tips?
   2. Which ones? Why and why not?
   3. Are there areas you think are missing or you would like more information?
   4. Are there areas you think are not helpful or make you feel uncomfortable?
6. Let's talk about the appointment reminders
   - i. How have they been helpful/not helpful? Please explain.
   - ii. Have you missed an appointment whilst being in the study? Why?
7. Let's talk about the symptom reporting
   - How have you used or not used this aspect of the system. If yes, please describe. In your view, what has worked well and what hasn't worked well? What about the waiting? How did you feel while waiting for a call? Is the wait for a call too long or acceptable? Do you like talking to a doctor/nurse? How would you feel about an automated (would need to explain) system?
8. Is there anything you have learned by using the system? Please describe and please be specific.
9. Suggestions: Are there any other things you would like the system to do or not do?

vii. *Revised Stigma Scale*

Revised stigma scale: items of subscales

Items (number from original scale)	Subscale	Alpha	Correlation with original subscale
24. I have been hurt by how people reacted to learning I have HIV.	Personalized stigma	.75	.90, $p < .01$
35. I have stopped socializing with some people because of their reactions of my having HIV.			
36. I have lost friends by telling them I have HIV.			
17. I am very careful who I tell that I have HIV.	Disclosure	.73	.74, $p < .01$
25. I worry that people who know I have HIV will tell others.			
7. I feel that I am not as good a person as others because I have HIV.	Negative self-image	.84	.85, $p < .01$
12. Having HIV makes me feel unclean.			
15. Having HIV makes me feel that I'm a bad person.			
14. Most people think that a person with HIV is disgusting.	Public attitudes	.72	.71, $p < .01$
16. Most people with HIV are rejected when others find out.			

MAKERERE UNIVERSITY
SCHOOL OF MEDICINE
APPROVED
VALID UNTIL
10 FEB 2021
RESEARCH & ETHICS COMMITTEE
P.O.BOX 7072, KAMPALA

CFL YOUTH STUDY

viii. REVISED STIGMA SCALE: EVALUATION FORM (Baseline & Study End)

Section A: Patient Information	
1. Patient clinic # ☐☐☐☐☐	2. Study Number ☐☐☐☐☐
3. Date of Visit __/__/____ DD/MM/YYYY	4. Visit code ☐☐.☐

Section B: Demographics		
1.Patient initials	2.Residential Address	3. Phone contact
4. Next of Kin	5. Relationship with Patient (list relationship)	6. Phone contact
7. Date of Birth __/__/____ DD/MM/YYYY	8. Gender ☐ Male ☐ Female	9. Ethnicity (Tribe) (list) ________

Section C: Stigma assessment Subscale: Disclosure

1. I am very careful who I tell that I have HIV

☐ Yes
☐ No
☐ N/A

2. I worry that people who know I have HIV will tell others

☐ Yes
☐ No
☐ N/A

MAKERERE UNIVERSITY
SCHOOL OF MEDICINE
APPROVED
VALID UNTIL
10 FEB 2021
RESEARCH & ETHICS COMMITTEE
P.O.BOX 7072, KAMPALA

Section D: Subscale: Personalized Stigma
3.I have been hurt by how people reacted to learning I have HIV<br>☐Yes<br>☐No<br>☐Not applicable
4. I have stopped socializing with some people because of their reactions of my having HIV<br>☐Yes<br>☐No<br>☐Declined to answer<br>☐Not applicable
5. I have lost friends by telling them I have HIV<br>☐Yes<br>☐No<br>☐Not applicable<br>☐Declined to answer

Section E: Subscale: Negative Self-Image
**6.** I feel that I am not as good a person as others because I have HIV<br>☐ Yes<br>☐ No<br>☐ Dedlined to answer<br>☐ Not applicable
**7.** Having HIV makes me feel unclean<br>☐ Yes<br>☐ No<br>☐ Declined to answer
**8.** Having HIV makes me feel that I'm a bad person<br>☐Yes<br>☐No<br>☐Declined to answer

MAKERERE UNIVERSITY
SCHOOL OF MEDICINE
APPROVED
VALID UNTIL
10 FEB 2021
RESEARCH & ETHICS COMMITTEE
P.O.BOX 7072, KAMPALA

Section G: Sub-scale: Public attitudes

9. Most people think that a person with HIV is disgusting

☐ Yes

☐ No

☐ Declined to answer

8. Most people with HIV are rejected when others find out

☐ Yes

☐ No

☐ Declined to answer

**Study Staff**<br>Name	Signature	Date

**Return appointment**	**Return Appointment**
study visit Date: ___/___/______ DD/MM/YYYY	(Non-Study Visit: ___/___/______ DD/MM/YYYY
**Adapted fromStigma Scale Revised: Reliability and Validity of a Brief Measure of Stigma for HIV_ Youth. Ref:** ***K. Wright et al.*** **Journal of Adolescent Health**	

MAKERERE UNIVERSITY
SCHOOL OF MEDICINE
APPROVED
VALID UNTIL
10 FEB 2021
RESEARCH & ETHICS COMMITTEE
P.O.BOX 7072, KAMPALA

CFL YOUTH STUDY

ix. DEMOGRAPHIC, SOCIO-ECONOMIC AND MEDICAL EVALUATION FORM

Section A: Patient Information	
3. Patient clinic # ☐☐☐☐☐	4. Study Number ☐☐☐☐☐
3. Date Study Registration<br>__/__/____<br>DD/MM/YYYY	4. Visit Code ☐☐.<br>5. Enrolled in other studies?<br>☐ Yes ☐ No<br>If Yes, Note here ______________

Section B: Demographics			
1.Patient initials<br>☐☐☐	2.Residential Address<br>☐☐☐☐☐☐☐☐		3. Phone contact<br>☐☐☐☐☐☐☐☐
4. Next of Kin<br>a. ----------------------<br>b. ----------------------	5. Relationship with Patient<br>a. --------------------<br>b. --------------------		6. Phone contact<br>a. ------------------<br>b. ------------------
7. Date of Birth<br>__/__/____<br>DD/MM/YYYY	8. Gender<br>☐ Male<br>☐ Female	9. Ethnicity<br>(Tribe)	

MAKERERE UNIVERSITY SCHOOL OF MEDICINE APPROVED VALID UNTIL 10 FEB 2021 RESEARCH & ETHICS COMMITTEE P.O.BOX 7072, KAMPALA

Section C: Social Assessment

11.Known HIV test results?

☐ Positive
☐ Negative.

12. Date of HIV test ___/___ /______

DD/MM/YYYY

12a) Previous VL test results (N/A if initiating ART)

12b) Date of test results

13. Do you have a partner?

☐ No(if no, go to 14)
☐ Yes

13 a. If Yes, for how long have you been with your partner

☐☐ (Yrs., Months)

11a. If positive are you receiving ART treatment?

☐ Yes
☐ No

11b. If yes, list ART start dates

___/___/______

DD/MM/YYYY

11c: ART Regimen: (Use MoH Codes)

14. Have you shared your test results with any one? ☐ Yes ☐ No

14. If yes, with who? List relationship

a) ----------------------

b) ----------------------

MAKERERE UNIVERSITY
SCHOOL OF MEDICINE
APPROVED
VALID UNTIL
10 FEB 2021
RESEARCH & ETHICS COMMITTEE
P.O.BOX 7072, KAMPALA

Section D: Education Living Standards

1. Highest level of Schooling Completed	2. Are you currently working?
☐ No Formal Education.<br>☐ P1-P4<br>☐ P5-P7<br>☐ S1-S4.<br>☐ S5-S6<br>☐ Technical/ University	☐ Yes ☐ No<br><br>2a. If yes what is your current occupation?<br><br>…………………………………………
3. What is your household income /month?<br><br>*(Consider ALL sources of income, self or guardian)*<br><br>☐ Less than 50,000<br>☐ 50,000/= and 100,000<br>☐ 100,001/= and 500,000<br>☐ 500,001/= and 1,000,000<br>☐ Above 1,000,000<br>☐ Declined to answer	4. Number of people supported on income?<br><br>☐☐
5. Number of Children ☐ <18 In household (# each age)<br><br>1. 0-4 Years ☐<br>2. 5-9 Years ☐<br>3. 10-14 Years ☐<br>4. 15 –18year ☐	6. Housing Description<br><br>☐ Mud floor walls with no ventilation<br>☐ Mud floor brick walls with one small window<br>☐ Brick house with enough windows<br>☐ Brick house with enough glass windows and well ventilated<br>☐ Other(Camp, Unipot etc)

MAKERERE UNIVERSITY
SCHOOL OF MEDICINE
APPROVED
VALID UNTIL
10 FEB 2021
RESEARCH & ETHICS COMMITTEE
P.O.BOX 7072, KAMPALA

Section E: Medical Drug Use Assessment

Medical history

1a. Do you have any complaint today (Record any Symptoms reported and duration)?

__________ __________ __________ __________ __________

1b)Have you visited a health facility in the past 3 months? Yes ☐ No☐(if no go to 2)

1c)How much money have you spent on health related care during this period? State amount in UGX

☐ ☐ ☐ ☐ ☐

2. Ever Smoked Tobacco/Marijuana	3. Ever used Alcohol Yes☐ ☐No
☐ Yes ☐Present ☐ Past Duration☐☐(months)	If yes, specify period, and duration
☐No	☐Present ☐ Past
	☐☐ Duration(months)

5-a. Prior diagnosis of TB ☐No ☐Yes (If yes, complete below)

☐ Pulmonary ☐ suspected (+Hx, +PE)

☐ Extra Pulmonary ☐ presumptive (+Tx, +CXR)

☐ confirmed (+AFB Smear, +CXR)

5-b. If yes to #5, TB Rx start stop dates?

Start ___/___/______ DD/MM/YYYY Stop ___/___/______ DD/MM/YYYY

Start ___/___/______ DD/MM/YYYY Stop ___/___/______ DD/MM/YYYY

MAKERERE UNIVERSITY
SCHOOL OF MEDICINE
APPROVED
VALID UNTIL
10 FEB 2021
RESEARCH & ETHICS COMMITTEE
P.O.BOX 7072, KAMPALA

SECTION F: ART HISTORY USE MOH CODES

1. ARV Regimen	start date	end date	change /stop reason<br><br>toxicity/ drug interactions	ART-source<br><br>center

2. TB TREATMENT HISTORY

Anti TB regimen	Start date	End date	Change /Stop reason (Toxicity/ completed/ side effects)	TB treatment<br><br>center

MAKERERE UNIVERSITY
SCHOOL OF MEDICINE
APPROVED
VALID UNTIL
10 FEB 2021
RESEARCH & ETHICS COMMITTEE
P.O.BOX 7072, KAMPALA

3. How often have you missed your ARV medication for the following reasons(since past visit: on follow-up) Tick all that apply

**Reason**	**Never**	**Rarely**	**Sometimes**	**Often**
Away from home				
Simply forgot				
Had too many pills to take				
Wanted to avoid side-effects				

Did not want people to notice you taking them				
Felt sick, depressed or overwhelmed				
Ran out of drugs				
Felt healthy				
Had a change in daily routine				

**Section G: Current Clinical Status/Therapy**	
1. Current medications<br>☐ Cotrimoxazole ☐ Corticosteroids<br>☐ Antibiotics ___________<br>☐ Other _____________	☐ Antidiabethes<br>☐ Anti Hypertension<br>☐ Chemotherapy<br>☐ Oral/injectable contraception
2. In the last weeks have you taken herbal or traditional medications?<br>☐ Yes ☐ No	2-a. If yes to # 2, why?<br>Increase strength, ☐ Yes ☐ No<br>Increase appetite, ☐ Yes ☐ No<br>Make more blood (hematinic) ☐ Yes ☐ No<br>Increase immunity ☐ Yes ☐ No<br>Other ---------------- ☐ Yes ☐ No

**Section H. Laboratory tests**		
Viral load	☐ Yes	☐ No
Blood storage	☐ Yes	☐ No
DBS	☐ Yes	☐ No

MAKERERE UNIVERSITY
SCHOOL OF MEDICINE
APPROVED
VALID UNTIL
10 FEB 2021
RESEARCH & ETHICS COMMITTEE
P.O.BOX 7072, KAMPALA

**Previous lab results:**	Viral Load<br>Date of blood draw<br>☐☐/☐☐/☐☐☐☐<br>Value: ☐☐☐☐☐☐<br>If not done Tick "NOT APPLICABLE" ☐<br>State reasons:<br>Blood not collected ☐<br>Sample clotted ☐<br>Test not yet due ☐<br>Other ☐ (specify)…………	CD4<br>Date of blood draw<br>☐☐/☐☐/☐☐☐☐<br>Value: ☐☐☐☐☐☐<br>If not done Tick "NOT APPLICABLE" ☐<br>State reasons:<br>Blood not collected ☐<br>Sample clotted ☐<br>Test not yet due ☐<br>Other ☐<br>(specify)…………

MAKERERE UNIVERSITY
SCHOOL OF MEDICINE
APPROVED
VALID UNTIL
10 FEB 2021
RESEARCH & ETHICS COMMITTEE
P.O.BOX 7072, KAMPALA

Section I: Clinical Plan

1.Is the patient changing/Starting ART regime today?	1a. if yes to #1
☐ Yes ☐ No	Current Regimen ☐☐☐☐☐☐<br>New regimen ☐☐☐☐☐☐<br>Give reason for changing ART regimen(PRINT)<br>Reason ____________________

**Study Personnel** Name	Signature	Date
**Return appointment**<br>study visit Date: ___/___/_____<br>CFL Youth Study (adapted from CFL RCT)	**Return Appointment**<br>(Non-Study Visit: ___/___/______<br>DD/MM/YYYY	

MAKERERE UNIVERSITY
SCHOOL OF MEDICINE
APPROVED
VALID UNTIL
10 FEB 2021
RESEARCH & ETHICS COMMITTEE
P.O.BOX 7072, KAMPALA

CFL YOUTH STUDY

x. KNOWLEDGE EVALUATION FORM

Section A: Patient Information		
1. Patient clinic # ☐☐☐☐☐	2. Study Number ☐☐☐☐☐	3. Visit code: ☐☐. ☐<br>4. Visit date___/___/______<br>DD/MM/YYYY

Section B: General Knowledge about HIV prevention, transmission and treatment

1. In the past month, have you heard or read any information about HIV/ AIDS?

☐ Yes
☐ No

2. From what source did you receive this information?

☐ Internet
☐ Friend or relative
☐ Television
☐ Study project
☐ Health workers
☐ Others; Specify………………………………………………………

MAKERERE UNIVERSITY
SCHOOL OF MEDICINE
APPROVED
VALID UNTIL
10 FEB 2021
RESEARCH & ETHICS COMMITTEE
P.O.BOX 7072, KAMPALA

3. In your opinion is there a difference between HIV and AIDS?

☐ Yes
☐ No
☐ Do not Know

4. Do you think a person can get infected by sharing utensils and clothes with a person who has HIV?

☐ Yes
☐ No
☐ Do Not know

5. Can a person get HIV through mosquito bites?

☐ Yes
☐ No

☐ Do not Know

6. Can a healthy HIV positive person transmit HIV?

☐ Yes
☐ No
☐ Do not know

Section C: General Knowledge about TB prevention, transmission and treatment

1. Do you think anti-TB drugs can reduce TB transmission from one person to another?

☐ Yes
☐ No
☐ Do not know

2. In your opinion who can get infected with TB?

☐ Only people with HIV
☐ Children below 5 years
☐ Anyone

3. Which parts of the body can be affected by TB?

☐ The bones
☐ The lungs
☐ abdomen
☐ Skin
☐ Any part

4. How is TB spread?

☐ Through the air
☐ Through blood
☐ Don't know

MAKERERE UNIVERSITY
SCHOOL OF MEDICINE
APPROVED
VALID UNTIL
★ 10 FEB 2021 ★
RESEARCH & ETHICS COMMITTEE
P.O.BOX 7072, KAMPALA

5. The following are signs and symptoms of TB:

		Agree (1)	Disagree (2)	Don't know (3)
i.	Rash	☐	☐	☐
ii.	Cough	☐	☐	☐
iii.	Cough that lasts more than 2 weeks	☐	☐	☐

iv.	Coughing up blood	☐	☐	☐
v.	Severe headache	☐	☐	☐
vi.	Nausea	☐	☐	☐
vii.	Weight loss	☐	☐	☐
viii.	Chest pain	☐	☐	☐
ix.	Difficulty in breathing	☐	☐	☐

6. The following are ways in which people with TB can take good care of themselves and others?

		Agree (1)	Disagree (2)	Don't know (3)
i.	Eat a balanced diet	☐	☐	☐
ii.	Avoiding public places and social gatherings	☐	☐	☐
iii.	Take their medicine with food to avoid	☐	☐	☐
	Stomach upset.	☐	☐	☐
iv.	Use a face mask when coughing/sneezing	☐	☐	☐
v.	keep clinic appointments	☐	☐	☐
vi.	Smoke	☐	☐	☐
vii.	Not drink alcohol	☐	☐	☐
viii.	Always important to have Direct observed Treatment	☐	☐	☐

MAKERERE UNIVERSITY
SCHOOL OF MEDICINE
APPROVED
VALID UNTIL
10 FEB 2021
RESEARCH & ETHICS COMMITTEE
P.O.BOX 7072, KAMPALA

7. Anti TB medication should be taken for a period of?

- ☐ 2 months
- ☐ 2 to 6 months
- ☐ 6 to 9 months
- ☐ Don't know

8. All family members living with a TB patient should be screened for TB in a health facility.

- ☐ Agree

☐ Disagree

☐ Don't know

9. Can TB get cured?

Yes (1) ☐ No(2) ☐ Don't know(3) ☐

Section D: Knowledge about HIV prevention, transmission and treatment

1. The following is true about HIV/AIDS

		Agree (1)	Disagree (2)	Don't know (3)
i.	It is a disease which has no cure.	☐	☐	☐
ii.	It reduces the body's natural defense against illness.	☐	☐	☐
iii.	It is caused by a virus called HIV.	☐	☐	☐
iv.	Everyone with HIV has AIDS	☐	☐	☐
v.	Some may show signs of AIDS in a shorter time than others.	☐	☐	☐
vi.	You can slow progression of HIV to AIDS by getting into care and living positively	☐	☐	☐

2. The following is true about Preventing transmission of HIV to others.

		Agree (1)	Disagree (2)	Don't know (3)
i.	Taking ARV drugs is important.	☐	☐	☐
ii.	Use condoms when having sex.	☐	☐	☐
iii.	Abstinence	☐	☐	☐
iv.	Being faithful	☐	☐	☐

MAKERERE UNIVERSITY
SCHOOL OF MEDICINE
APPROVED
VALID UNTIL
10 FEB 2021
RESEARCH & ETHICS COMMITTEE
P.O.BOX 7072, KAMPALA

3. Do you think that ARV drugs are effective in treating HIV?

☐ Agree

☐ Disagree

☐ Do not know

4. The following is true about Safe Male circumcision

		Agree (1)	Disagree (2)	Don't know (3)
i.	It is the removal of fore skin	☐	☐	☐

ii.	It protects against HIV	☐	☐	☐
iii.	One can't get HIV if they are circumcised	☐	☐	☐
iv.	Use condoms whenever they have sex	☐	☐	☐
v.	It reduces sexual performance	☐	☐	☐
vi.	Babies heal faster than adult men	☐	☐	☐

5. The following is true about preventing transmission of HIV from mother to the baby:

	Agree (1)	Disagree (2)	Don't know (3)
i. It is important for the infected mother to take ARV medication regularly during Pregnancy	☐	☐	☐
ii. The baby can get HIV through breast feeding milk.	☐	☐	☐
iii. It is important for the new born baby to be given HIV preventive therapy.	☐	☐	☐
iv. All mothers should be screened for HIV at their antenatal visit.	☐	☐	☐
v. A baby can't get HIV after birth	☐	☐	☐
vi. HIV can be transmitted through breast milk	☐	☐	☐

MAKERERE UNIVERSITY
SCHOOL OF MEDICINE
APPROVED
VALID UNTIL
10 FEB 2021
RESEARCH & ETHICS COMMITTEE
P.O.BOX 7072, KAMPALA

6. The following is true about condom use:

		Agree (1)	Disagree (2)	Don't know (3)
i.	You must ALWAYS use a condom even if you are already HIV positive	☐	☐	☐
ii.	Condoms prevent you from getting infected with a resistant type of virus	☐	☐	☐
iii.	Couples can reduce risk by using condoms correctly and consistently during sex	☐	☐	☐
iv.	To protect from HIV reinfection and transmission, abstain or use condoms EVERY TIME you have sex	☐	☐	☐

7. The following is true about HIV/AIDS

		Agree (1)	Disagree (2)	Don't know (3)
i.	AIDS is a disease which has no cure	☐	☐	☐
ii.	Reduces the body's natural defense against illness	☐	☐	☐

	Agree (1)	Disagree (2)	Don't Know (3)
iii. Everyone with HIV has AIDS	☐	☐	☐
iv. Bodies show signs of AIDS in a shorter time	☐	☐	☐
v. Progression of HIV to AIDS can be slowed by getting into care and living positively	☐	☐	☐
vi. Someone on ARVs who does not take ARV well will develop resistant virus	☐	☐	☐

8. HIV Discordance is when a partner in a stable relationship tests HIV positive while the other tests HIV negative. Discordance is common. The following is true about discordance:

	Agree (1)	Disagree (2)	Don't Know (3)
i. Discordance is a sure sign that someone has been unfaithful.	☐	☐	☐
ii. A couple can remain discordant for a very long time	☐	☐	☐
iii. The negative partner is always at risk.	☐	☐	☐
iv. Couples can reduce risk by using condoms correctly and consistently during sex	☐	☐	☐
v. HIV positive partner should start ARV's as soon as possible	☐	☐	☐

9. The following is true about ART

	Agree (1)	Disagree (2)	Don't Know (3)
i. When one is tested for HIV and started on ARVs that first treatment is called first line.	☐	☐	☐
ii. Your clinician may decide to change your drugs to a new type called second line because the first line drugs have stopped working.	☐	☐	☐
iii. First line stops working If you don't adhere well	☐	☐	☐
iv. First line stops working if you get a resistant virus or have been on the drugs for a very long time	☐	☐	☐
v. Main cause of first line failure is poor/ inconsistence drug swallowing and many other reasons.	☐	☐	☐

Study Staff Name	Signature	Date ___/___/______ DD/MM/YYYY

MAKERERE UNIVERSITY
SCHOOL OF MEDICINE
APPROVED
VALID UNTIL
10 FEB 2021
RESEARCH & ETHICS COMMITTEE
P.O. BOX 7072, KAMPALA

CFL YOUTH STUDY

xi. SEXUAL BEHAVIOR QUESTIONNAIRE

Section A: Patient Information		
1. Patient clinic # ☐☐☐☐☐	2. Study Number ☐☐☐☐☐	3. Visit code: ☐☐. ☐<br>4. Visit date___/___/______<br>DD/MM/YYYY

Section B: Attitudes and sexual practices

5. Do you currently have a regular/ steady partner?

☐ Yes

☐ No (skip to 7)

6. If yes to Qn 5 above, how long have you been with this partner?

☐☐Years ☐☐Months

7. Have you ever had a partner =/ >10 years older than you?

☐ Yes

☐ No

8. At what age was your sex debut?

☐☐Years ☐☐(N/A)

9. After knowing your HIV Status, has your attitude towards having sex changed?

☐ Yes

☐ No

10. If yes to Qn 9 above, which of the following best describes your change in sexual practices?

MAKERERE UNIVERSITY
SCHOOL OF MEDICINE
APPROVED
VALID UNTIL
10 FEB 2021
RESEARCH & ETHICS COMMITTEE
P.O.BOX 7072, KAMPALA

☐ Abstinence

☐ Use of condoms

☐ Reduced number of sexual partners

☐ Reduced number of sexual encounters

☐ I lost interest in sex

☐ Increased number of sexual partners

☐ Increased number of sexual encounters

11. HIV positive persons should abstain from having sexual intercourse?

☐ Agree

☐ Disagree

☐ Do not know

12. When was the last time you had sexual intercourse?

☐ Hours ago

☐ Days ago

☐ Weeks ago

☐ Months ago

☐ Years ago

☐ Never

13. If months or years ago, what is the most common reasons which prevented you from having sexual intercourse?

☐ No partner

☐ My health status

☐ Health status of my partner

☐ Am abstaining

☐ Other (specify)--

MAKERERE UNIVERSITY
SCHOOL OF MEDICINE
APPROVED
VALID UNTIL
10 FEB 2021
RESEARCH & ETHICS COMMITTEE
P.O.BOX 7072, KAMPALA

14. The last time you had intercourse did you use a condom?

☐ Yes

☐ No

☐ Do not remember

15. The last time you had intercourse had you been drinking alcohol or marijuana in the few hours before?

☐ Yes

☐ No

☐ Do not Know

16. How many times in the last 2 months have you had sex without a condom?

☐ None

☐ 1-3

☐ 3-10

☐ >10

17. Do you regularly use condoms?

☐ Yes

☐ No

18. In the last 3 months, have you had any abnormal discharge/ ulcer/wound in your genito-urinary part?

☐ Yes ☐ No (skip to 20)

19. If Yes, did you receive treatment

☐ Yes, went to health worker

☐ Yes, I self-medicated

☐ No, I did not

20. Have you ever had forced or coerced sex?

☐ Yes

☐ No (if no end here)

MAKERERE UNIVERSITY
SCHOOL OF MEDICINE
APPROVED
VALID UNTIL
10 FEB 2021
RESEARCH & ETHICS COMMITTEE
P.O.BOX 7072, KAMPALA

21. If yes to Qn 20 above, did you inform any one?

☐ Yes

☐ No

22. If yes to Qn 21 above, whom did you inform?

☐ Police

☐ Friend

☐ Relative (Specify relationship eg, Mum, sister, aunt) ……………..

Study Staff Name / Initials	Signature	Date
☐☐☐		___/___/______ DD/MM/YYYY

xii.

C4L-Youth Study

COVID-19 KNOWLEDGE ASSESSMENT AMONG YOUTH (BASELINE ONLY)

Section A: Patient Information							
1. Clinic number#	2. STUDY Number :		3. Date of Visit ____/____/_______ DD/MM/YYYY				
Have you ever heard or know about Corona Virus disease 2019? Yes ☐ No ☐ (if no stop) Where do you get your knowledge about Coronavirus?	Family / friends ☐	Radio ☐	Newspaper ☐	Social media ☐	Internet ☐	Religious leader ☐	Medical personnel ☐

Questions developed using information from WHO https://www.who.int/news-room/q-a-detail/q-a-coronaviruses

Section B. Do you know any Symptoms of COVID-19							
Symptoms	Yes	No	Days	Symptoms	Yes	No	Days
Fever				Aches and pains			
Dry cough				Nasal congestion/runny nose			
Sneezing				Sore throat			
Loss of smell				Loss of taste			
Shortness of breath				Diarrhea			
Have you had any symptoms above? List symptoms and action taken							

Section C. Knowledge about PROTECTION				Section D. Practices about PROTECTION (last 7 days)		
	Agree	Disagree	Don't know	Yes	No	NA
Regularly and thoroughly clean your hands with an alcohol-based hand rub						
Regularly and thoughly wash your hands with soap and water.						

MAKERERE UNIVERSITY
SCHOOL OF MEDICINE
APPROVED
VALID UNTIL
10 FEB 2021
RESEARCH & ETHICS COMMITTEE
P.O.BOX 7072, KAMPALA

Maintain at least 1 metre (3 feet) distance between yourself and anyone who is coughing or sneezing.						
Avoid touching eyes, nose and mouth						
Covering your mouth and nose with your bent elbow when you cough or sneeze.						
Covering your mouth and nose with tissue when you cough or sneeze. Then dispose of the used tissue immediately						
Stay home if you feel unwell						
Keep up to date on the latest COVID-19 hotspots						
Wear a mask if you are ill with COVID-19 symptoms (especially coughing)						
Wear a mask if you are looking after someone who has COVID-19 symptoms						
Disposable face mask that can only be used once						

MAKERERE UNIVERSITY
SCHOOL OF MEDICINE
APPROVED
VALID UNTIL
10 FEB 2021
RESEARCH & ETHICS COMMITTEE
P.O.BOX 7072, KAMPALA

Section D. Other knowledge				
	Knowledgeable	Partially	Not knowledgeable	
What would you do if you have symptoms of Coronavirus?<br>Answer 1) Self quarantine 2) Wear a mask 3) Call help line number				
Do you have with you any help line number? Which one would you call if you or anyone near you has COVI-D symptoms?				
Should you put a mask to protect yourself?<br>Answer: Only wear a mask if you are ill with COVID-19 symptoms (especially coughing) or looking after someone who may have COVID-19.				
Do you know how to use a mask?<br>Answer. 1) Before touching the mask, clean hands with an alcohol-based hand rub or soap and water 2) Place the mask to your face. 3) Pull down the mask's bottom so it covers your mouth and your chin. 4) After use, take off the mask and and Discard the mask in a closed bin immediately after use 5) Perform hand hygiene after touching or discarding the mask – Use alcohol-based hand rub or, if visibly soiled, wash your hands with soap and water				

Questions developed using information from WHO https://www.who.int/emergencies/diseases/novel-coronavirus-2019/advice-for-public/myth-busters

Section E. Knowledge about MYTHS					Section D. Practices about MYTHS (last 7 days)		
	Agree	Disagree	Don't know		Yes	No	Not sure
Exposing yourself to the sun or to temperatures higher than 25C degrees prevents the coronavirus disease (COVID-19)							
Being able to hold your breath for 10 seconds or more without coughing or							

feeling discomfort means you are free from the coronavirus disease (COVID-19) or any other lung disease.							
Drinking alcohol protects you against COVID-19 and can be dangerous							
COVID-19 virus cannot be transmitted in areas with hot and humid climates							
Taking a hot bath prevents the new coronavirus disease							
The new coronavirus is transmitted through mosquito bites.							
Hand dryers effectively kill the new coronavirus?							
Spraying alcohol or chlorine all over your body kills the new coronavirus							
Eating garlic prevents infection with the new coronavirus							
The new coronavirus affects older people, and younger people are not susceptible							
Antibiotics are effective in preventing and treating the new coronavirus							
Chloroquine is effective in preventing and treating the new coronavirus							

Section F. HIV care

MAKERERE UNIVERSITY
SCHOOL OF MEDICINE
APPROVED
VALID UNTIL
10 FEB 2021
RESEARCH & ETHICS COMMITTEE
P.O.BOX 7072, KAMPALA

ART medications

Do you have antiretroviral drugs with you at the moment? ☐ Yes ☐ No

Are you taking your ART? ☐ Yes ☐ No

Rate your adherence in the last month ______

Analogue visual scale 0-100

HIV care

Access to HIV services ☐ Walking ☐ Public transport (Matatu) ☐ boda boda ☐ Private vehicle

Do you have any concerns about your own health? ☐ Yes ☐ No

Do you think HIV infection increases the risk of acquiring COVID-19? ☐ Yes ☐ No

Has your HIV care been affected by any of the following?

☐ Drug stock out ☐ No public transport ☐ Halt of private vehicles ☐ Fear of acquiring HIV at the clinic

☐ Sickness of health care workers ☐ Unavailability of HIV services due to the response to COVID-19

Do you think your HIV care will be affected by any of the following?

☐ Drug stock out ☐ No public transport ☐ Halt of private vehicles ☐ Fear of acquiring HIV at the clinic

☐ Sickness of health care workers ☐ Unavailability of HIV services due to the response to COVID-19

Other medication

Were you prescribed drugs for any of the following?	Are currently taking them?
☐ Diabethes	☐ Yes ☐ No
☐ Hypertension	☐ Yes ☐ No
☐ Anticonvulsants	☐ Yes ☐ No
☐ Contraceptives	☐ Yes ☐ No
☐ Anti TB drugs	☐ Yes ☐ No
Are you taking any herb to prevent CORVID-19/boost your immunity?	☐ Yes ____________ ☐ No

MAKERERE UNIVERSITY
SCHOOL OF MEDICINE
APPROVED
VALID UNTIL
10 FEB 2021
RESEARCH & ETHICS COMMITTEE
P.O.BOX 7072, KAMPALA
