# Supplementary material for: Effectiveness of interactive voice response-call for life mHealth tool on adherence to anti-retroviral therapy among young people living with HIV: A randomized trial in Uganda
Source: PLoS One. 2024 Nov 19;19(11):e0308923. doi: 10.1371/journal.pone.0308923 (PMC11575762; doi:10.1371/journal.pone.0308923)
Supplement: Attachment — Submitted filename: Review Comments -PLOS1-pdf.pdf [file pone.0308923.s006.pdf]

The authors describe A randomized trial to assess the effectiveness of Interactive Voice Response-Call for life mHealth tool on adherence to Anti-Retroviral Therapy among young people living with HIV in Uganda. The manuscript addresses an important problem and contributes to the evidence base in support of digital interventions in health and merits publication. However, the authors need to address the following comments to make it acceptable for publication.

0. The abbreviation SOC in the abstract should be written out in full. Further all abbreviations and acronyms should written out in full when first used (except where the acronym is well established and generally known by the community).

1. There are paragraphs in the introduction that are not necessary and in fact distract from the focus of the manuscript. Paragraphs in lines 45-63 have little value in the paper and simply distract. It would be more useful to provide a brief overview of digital and mHealth interventions and their promise in improving health. Indeed a definition of mHealth or similar digital interventions need to come right after the first three paragraphs.

2. The last paragraph in introduction could be improved by rewriting to start by describing the CFL-IVR together with its aims and benefits and and concluding with what the authors aimed to do. In its current form the paragraphs is not standard.

3. Colons in many places in a none standard way. The authors should ensure colons are appropriately used. For example there is no need to include colons in section headings – prominence to section headings can be given by bolding.

4. Lines 153-157, state the statistical tests used

5. Line 154, i) be clear that the metric assessed between arms is proportion of individuals with VL <1000 copies/mL

6. Line 165, is it true that such a stringent alpha, 0.005, was used? If true, it is unusual for a study of this size.

7. Improve tables to publication standards, importantly no vertical lines – check how tables in similar publications are presented.

8. Tables and Figures should be standalone, so improve legends and titles by making more descriptive.
-Abbreviations used in tables such SD, Q1, Q3 should be described in notes below the table, and -clarify what the data in the table are. For example be clear that the denominator for % in tables are N for the arm.
-Avoid very long tables, table 1 for examples runs through three pages! This needs to be shortened.
-State what statistical test the p-values are from.

9. In the first paragraph of the discussion also include the findings in the SOC so the comparison is clear to the reader.
- Also can the authors explain why retention was higher in the present study relative to previous mHealth studies.

9. References require a thorough check and improvement. References in lines 478, 477, 445, 443, 418, 359 etc are not standard.

10. Please check that the manuscript length is within the word limit for articles in this journal.

&&&&& Read through tables and discussion
