# Supplementary material for: Effectiveness of interactive voice response-call for life mHealth tool on adherence to anti-retroviral therapy among young people living with HIV: A randomized trial in Uganda
Source: PLoS One. 2024 Nov 19;19(11):e0308923. doi: 10.1371/journal.pone.0308923 (PMC11575762; doi:10.1371/journal.pone.0308923)
Supplement: Attachment — Submitted filename: Response to Reviewers.doc [file pone.0308923.s007.doc]

Dear Editor,

We thank you and the two reviewers for time taken to provide constructive comments to better the manuscript. Please find below, the point-by- point responses (***bold italics***) to comments raised and line numbers where the changes are made.

**Re: Point by Point response to comments raised by the reviewers and editors on PONE-D-23-37288: Effectiveness of Interactive Voice Response-Call for life mHealth tool on adherence to Anti-Retroviral Therapy among young people living with HIV: A randomized trial in Uganda**

ACADEMIC EDITOR’S COMMENTS

- Please follow the journal formats and be concise

***Thanks, this guidance has been followed in the resubmission for the revised manuscript***

- Be very clear with the sample size determination: You have two outcomes (viral load suppression and retention). You state the difference between arms though not showing whether on viral load or retention! How are both outcomes taken care of in the sample size estimation?
  ***Sample size determination was based on the primary outcome, which is viral suppression at 12 months, this has been stated in the revision as per lines 146-148: (*The sample size *calculation was based on primary outcome using openEpi.com sample size calculation formula for randomized clinical trials)***

***The difference between arms have been shown for both the primary outcome and the secondary outcomes in table 2 (Viral suppression at 12 months) and table 3 (Retention in care at 12 months, and 6 months visit),***

- Have a clear section on randomization concealment.
  ***The Randomization concealment section has been made clearer, under the study procedures subsection, thanks. Lines 104-115***
- Line 167/168 talks of a final model! what was in this model and how was it estimated?
  ***This has been included under the statistical analysis section we used log-binomial regression model, lines 155-159***
- Please note that the write-up should at all times compare between arms.
  ***Thanks for the guidance, this has been put into consideration, and updated the manuscript***.
- Why include p-values in the baseline table? what do they mean? please check on how to report the baseline table of randomized controlled trials.

***After the guidance from the reviewers too, we have updated the baseline characteristics table, and calculated the standardized mean difference. This error has been rectified, thanks for pointing it out. Table 1, line 199-***

REVIEWER#1

Specific comments:
1. (line 17) I didn't understand the clause "month 6 and 12".

***Sorry for incomplete clause, it has been updated to; “***T***he primary outcome was viral suppression defined as HIV RNA below 1,000 copies per mL at 12 months with retention in care and viral suppression at month 6 as a secondary outcome.”***  Line numbers 16-18
2. (Line numbers 94-98) I don't think "Health" and "Hospital" should be capitalized when they are not part of a proper noun.

***Thanks for pointing out this, it has been taken note of and removed the capitalization in the revised version.***
3. (line 108) Please describe the algorithm or program that was used to randomize the order of the envelopes.

***Block randomization at 1:1 ratio was done to either standard of care (usual care) or Call for life-IVR with standard of care. Computerized varying block of six randomization was done by an independent statistician who kept the treatment code list.***  Line numbers 103-107
4. (line 132) I don't believe "Intervention" should be capitalized.

***Apologies once again, this has been rectified***.
5. ( Line numbers 138-139) There should be an ethics section of the manuscript that includes IRB and trial registration as well as full information on how consent and assent were collected.

***Thanks for the guidance, the ethics section is before the results section (***line numbers 172-181***) and these Line numbers have been added to the ethical and approval process*** ***section***
6. ( Line numbers 143-144) Again, a strange sentence when describing the month 6 and month 12 visits.

***This has been rectified to “Viral load (VL) testing was performed at baseline (for those on ART for a duration of 6 months),*** ***repeat VL samples taken at month 6, and month12 study visits. Participants were seen at month 6, and 12 visits and all procedures were repeated on these visits.”***  Line numbers 138-140
7. ( Line numbers 147-152) These two sentences are both incomplete. Plus, the openepi.com reference should be included in the bibliography and, again, strange capitalization. I suggest writing these as complete sentences and not just dumping output in the manuscript.

***This has been revised with the phrase below: “The sample size calculation was based primary outcome using openEpi.com sample size calculation formula for randomized clinical trials (Kevin M. Sullivan, A. D., Minn M. Soe ;. (2007). "Sample Size for a Cross-Sectional, Cohort, or Clinical Trial Studies." 2019, from https://www.openepi.com/SampleSize/SSCohort.htm***). ***We used a two-sided significance level (alpha) of 0.05, and a power of 80, with a ratio of unexposed: exposed is 1.*** ***Primary end point was viral load suppression at month 12, defined as viral load count below detection (<1000 copies/ml); We estimated 79% viral suppression in the control arm based on previous results in the study area (Ministry of Health, Uganda. Uganda Population-based HIV Impact Assessment (UPHIA) 2016-2017: Final Report. Kampala, Ministry of Health, Jul 2019) , with 94% virally suppressed in the intervention arm (De Costa, A., N. Shet A Fau - Kumarasamy, P. Kumarasamy N Fau - Ashorn, B. Ashorn P Fau - Eriksson, L. Eriksson B Fau - Bogg, V. K. Bogg L Fau - Diwan and V. K. Diwan "Design of a randomized trial to evaluate the influence of mobile phone reminders on adherence to first line antiretroviral treatment in South India--the HIVIND study protocol." (1471-2288 (Electronic)). Factoring 10% loss to follow-up, the total sample size was estimated at 206 (103 for each arm).***  Line numbers 144-150

8. ( Line numbers 158-159) Run-on sentence. I think this sentence is supposed to define ITT but it does not.

***Thanks for pointing out this, the sentence has been updated since ITT was not used in the analysis. It has been updated as below, since per protocol analysis was used. This is the updated sentence “******Per protocol analysis was conducted including only the participants attending the month 12 study visit for assessment of the proportions of viral load suppression”.***  Line numbers165-166

9. ( Line numbers 165, 167) Why are you using different definitions of statistically significant?

***I have omitted the Confidence interval part and left the alpha of <0.05. Below is the statement in the revised manuscript***

***“******Viral load results at month 12 were analysed, proportions of those with detectable VL and those with suppressed VL were estimated in both arms; risk ratios and factors associated with viral load failure were calculated with a p-value of 0.05 taken as statistically significant.***” Line numbers 158-160

10. (line 186, table 1) Significance testing between intervention groups is generally frowned upon because a non-significant p-value does not indicate that groups are the same. For info on the topic in relation to baseline imbalance in randomized trials see Altman, <https://doi.org/10.2307/2987510> and Senn, <https://doi.org/10.1002/sim.4780131703>. My usual recommendation is to remove the significance testing from tables like this and use standardized mean differences (SMD) to assess imbalance (see Austin, <https://doi.org/10.1080/03610910902859574>). Most often, I see authors using an SMD of 0.1 or 0.2 as a threshold for assessing imbalance, though this could vary by field.

***Thanks for the recommendation, the significance testing has been removed*- and added calculated standardized mean difference.**

The baseline characteristics table has been revised with SMD as below:

| **Variable** | **Intervention**  **N=103** | **Standard of Care N=103** | **SMD^1^** |
| --- | --- | --- | --- |
| **Age** Mean (SD) | 22.5(1.93) | 22.3(2.25) | 0.09 |
| **Gender**  Male  Female | 22(21.4%)  81(78.6%) | 17(16.5%)  86(83.5%) | 0.13 |
| **ART status at entry**  ART naïve  On ART >6 months | 52(50.5%)  51(49.5%) | 57(55.3%)  46(44.7%) | 0.10 |
| **Baseline VL test results* (n=75*)**  ≥1000 copies/ml  <1000 copies/m | 18(17.5%)  33(64.7%) | 11(23.9%)  35(76.1%) | 0.25 |
| **Sexual Partner**  Yes  No | 94(91.3%)  9(8.7%) | 89(86.4%)  14(13.6%) | 0.15 |
| **Disclosure of status**  Yes  No | 95(92.2%)  8(7.8%) | 94(91.3%)  9(8.7%) | 0.04 |
| **Highest level of schooling**  No formal Education  Primary  Secondary  Technical | 2(1.9%)  69(67.0%)  29(28.2%)  3(2.9%) | 4(3.9%)  79(76.7%)  17(16.5%)  3(2.9%) | 0.30 |

^1^ Standardized mean difference between arms and continuous and categorical variables. SD: Standard Deviation, VL: viral load

*****75 participants were eligible for blood draws for viral load tests as per national treatment guide Line numbers

11. ( Line numbers 230-239) I believe this should be in the discussion section and not the results section

***Thanks for the recommendation, this has been moved to the end of the results section.***

REVIEWER#2

The authors describe A randomized trial to assess the effectiveness of Interactive Voice Response Call for life mHealth tool on adherence to Anti-Retroviral Therapy among young people living with HIV in Uganda. The manuscript addresses an important problem and contributes to the evidence base in support of digital interventions in health and merits publication. However, the authors need to address the following comments to make it acceptable for publication.

0. The abbreviation SOC in the abstract should be written out in full. Further all abbreviations and acronyms should write out in full when first used (except where the acronym is well established and generally known by the community).

***Thanks for this guidance, it was an oversight, it has been noted and all have been written in full at first use.***

1. There are paragraphs in the introduction that are not necessary and in fact distract from the focus of the manuscript. Paragraphs in Line numbers 45-63 have little value in the paper and simply distract. It would be more useful to provide a brief overview of digital and mHealth interventions and their promise in improving health. Indeed, a definition of mHealth or similar digital interventions need to come right after the first three paragraphs.

***Thanks for pointing out this and the advice has been followed. Line numbers 45-63 have been omitted, have moved the write up on mHealth right under the first three paragraphs.***  Line numbers 50-53

2. The last paragraph in introduction could be improved by rewriting to start by describing the CFLIVR together with its aims and benefits and concluding with what the authors aimed to do. In its current form the paragraphs is not standard.

***Thanks for the advice, although we had put the CFLIVR under methods, we have followed your advice and have added a summarized description of CFL-IVR in the introduction section.***  Line numbers 64-73

3. Colons in many places in a none standard way. The authors should ensure colons are appropriately used. For example, there is no need to include colons in section headings – prominence to section headings can be given by bolding.

***This has been taken note of, and we have removed the colons from the section headings and instead bolded the headings as per the advice.***

4. Line numbers 153-157, state the statistical tests used

***Log binomial regression analysis were done for primary, secondary analysis and factors associated with viral load failure (Tables 2,3, and 4). Pearson Chi-square test and Fischers Exact tests were used for the bivariate analysis on reasons for missing ART medication, table 5.***

5. Line 154, i) be clear that the metric assessed between arms is proportion of individuals with VL

***Thanks for pointing out, this has been updated***

6. Line 165, is it true that such a stringent alpha, 0.005, was used? If true, it is unusual for a study of this size.

***Apologies, this was a typo. The alpha has been rightly put at 0.05***

7. Improve tables to publication standards, importantly no vertical Line numbers – check how tables in similar publications are presented.

***Thanks for the guidance, the tables have been revised and all vertical Line numbers removed.***

8. Tables and Figures should be standalone, so improve legends and titles by making more descriptive. -Abbreviations used in tables such SD, Q1, Q3 should be described in notes below the table, and - clarify what the data in the table are. For example be clear that the denominator for % in tables are N for the arm. -Avoid very long tables, table 1 for examples runs through three pages! This needs to be shortened.

***The legends and titles have been improved, description to the tables added and the abbreviations described in the notes. The tables have been shortened. Table 1, baseline characteristics:***

Table 1. Baseline characteristics

| **Variable** | **Interventional N=103** | **Standard N=103** | **SMD^1^** |
| --- | --- | --- | --- |
| **Age** Mean (SD) | 22.5(1.93) | 22.3(2.25) | 0.09 |
| **Gender**  Male  Female | 22(21.4%)  81(78.6%) | 17(16.5%)  86(83.5%) | 0.13 |
| **ART status**  ART naïve  On ART >6 months | 52(50.5%)  51(49.5%) | 57(55.3%)  46(44.7%) | 0.10 |
| **Baseline VL test results* (n=75*)**  ≥1000 copies/ml  <1000 copies/m | 18(17.5%)  33(64.7%) | 11(23.9%)  35(76.1%) | 0.25 |
| **Sexual Partner**  Yes  No | 94(91.3%)  9(8.7%) | 89(86.4%)  14(13.6%) | 0.15 |
| **Disclosure of status**  Yes  No | 95(92.2%)  8(7.8%) | 94(91.3%)  9(8.7%) | 0.04 |
| **Highest level of schooling**  No formal Education  Primary  Secondary  Technical | 2(1.9%)  69(67.0%)  29(28.2%)  3(2.9%) | 4(3.9%)  79(76.7%)  17(16.5%)  3(2.9%) | 0.30 |

^1^ Standardized mean difference between arms and continuous and categorical variables. SD: Standard Deviation VL: Viral load

***** 75 participants were eligible for blood draws for viral load tests as per national treatment guide Line numbers

9. -State what statistical test the p-values are from.

***Thanks for this, which was erroneously missed, for the primary and secondary analysis, we used the log-binomial regression analysis, and the p-values are from the model (tables 2,3,4). Pearson Chi-square test and Fischer’s Exact tests for the bivariate analysis on reasons for missing ART (Table 5) were used. This has been explained on each table.***

10. In the first paragraph of the discussion also include the findings in the SOC so the comparison is clear to the reader. - Also can the authors explain why retention was higher in the present study relative to previous mHealth studies.

***The paragraph has been revised to capture info on the SOC, below is the revision “******Significant viral load suppression proportions of 73.6% in the intervention arm as compared to 51.9% in the standard of care arm at twelve months, and retention rate of 88.4% retention in care for the intervention arm as compared to 74.7% in the standard of care arm”.***  Line numbers 270-274

***Why the retention was higher in the present study relative to previous mHealth studies; “******these mHealth studies used short-text messaging service which might not be appealing to the youth compared to interactive voice response intervention. Further the IVR doesn’t depend or require the beneficiary to be literate”.***  Line numbers 279-281

11. References require a thorough check and improvement. References in Line numbers 478, 477, 445, 443, 418, 359 etc are not standard.

***Thanks, all the references have been improved and updated***

12. Please check that the manuscript length is within the word limit for articles in this journal.

***Thanks for the advice, the length has been adjusted***, ***removed, the limitations, authors’ contribution and funding sections.***

***Thanks,***

***Agnes Bwanika Naggirinya***
