# Supplementary material for: Effectiveness of interactive voice response-call for life mHealth tool on adherence to anti-retroviral therapy among young people living with HIV: A randomized trial in Uganda
Source: PLoS One. 2024 Nov 19;19(11):e0308923. doi: 10.1371/journal.pone.0308923 (PMC11575762; doi:10.1371/journal.pone.0308923)
Supplement: Attachment — Submitted filename: Point by point response 14062024.doc [file pone.0308923.s008.doc]

Dear Editor, 14-06-2024

We thank you for the time taken to provide constructive comments to better the manuscript. Below are the point-by-point responses (*bullets underlined*) to the comments raised and line numbers where the changes are made.

**Re: Point-by-Point response to additional comments raised by the editor on the manuscript “ PONE-D-23-37288R1. Effectiveness of Interactive Voice Response-Call for life mHealth tool on adherence to Anti-Retroviral Therapy among young people living with HIV: A randomized trial in Uganda”**

1. Please amend the manuscript submission data (via Edit Submission) to include author Maria Sarah Nabaggala.

Amendment has been done to include the deceased author (MSN)on the manuscript submission data

2. PLOS follows the Committee on Publication Ethics (COPE: [https://eur01.safelinks.protection.outlook.com/?url=https%3A%2F%2Fpublicationethics.org%2F&data=05%7C02%7Canaggirinya%40idi.co.ug%7C5acf615a6ef74155d11908dc7b6aafba%7C0d7c75a58002495ebb9f1f3108dcaa28%7C0%7C0%7C638520945346658723%7CUnknown%7CTWFpbGZsb3d8eyJWIjoiMC4wLjAwMDAiLCJQIjoiV2luMzIiLCJBTiI6Ik1haWwiLCJXVCI6Mn0%3D%7C0%7C%7C%7C&sdata=Q7T6mBOZMZ6Wv8J3%2BZIRJXKDg3rrFSZ4AqVNY1HHxiM%3D&reserved=0](https://eur01.safelinks.protection.outlook.com/?url=https%3A%2F%2Fpublicationethics.org%2F&data=05|02|anaggirinya@idi.co.ug|5acf615a6ef74155d11908dc7b6aafba|0d7c75a58002495ebb9f1f3108dcaa28|0|0|638520945346658723|Unknown|TWFpbGZsb3d8eyJWIjoiMC4wLjAwMDAiLCJQIjoiV2luMzIiLCJBTiI6Ik1haWwiLCJXVCI6Mn0%3D|0|||&sdata=Q7T6mBOZMZ6Wv8J3%2BZIRJXKDg3rrFSZ4AqVNY1HHxiM%3D&reserved=0)) guidelines for changes to authorship and therefore requires specific information relating to these changes. We would be grateful if you could, therefore, please provide us with the following:

a. A completed copy of the attached Request for Authorship Change(s) form

b. Written confirmation from all authors - including the authors being removed/added - stating that they agree to the changes being made

c. Specific details as to the contributions of the individual being added/removed, if applicable

d. Comments explaining why these changes are necessary and why they were not requested during review

There are no new changes, the author passed on during the review process and did not think necessary to add her on the submission portal, but she is still on the manuscript.

Thanks,

Agnes Bwanika Naggirinya
